# Chromosome-level assembly of *Lindenbergia philippensis* and comparative genomic analyses shed light on genome evolution in Lamiales

**DOI:** 10.3389/fpls.2024.1444234

**Published:** 2024-08-02

**Authors:** Bao-Zheng Chen, Da-Wei Li, Kai-Yong Luo, Song-Tao Jiu, Xiao Dong, Wei-Bin Wang, Xu-Zhen Li, Ting-Ting Hao, Ya-Hui Lei, Da-Zhong Guo, Xu-Tao Liu, Sheng-Chang Duan, Yi-Fan Zhu, Wei Chen, Yang Dong, Wen-Bin Yu

**Affiliations:** ^1^ College of Food Science and Technology, Yunnan Agricultural University, Kunming, Yunnan, China; ^2^ Yunnan Provincial Key Laboratory of Biological Big Data, Yunnan Agricultural University, Kunming, Yunnan, China; ^3^ Department of Plant Science, School of Agriculture and Biology, Shanghai Jiao Tong University, Shanghai, China; ^4^ Center for Integrative Conservation and Yunnan Key Laboratory for the Conservation of Tropical Rainforests and Asian Elephants, Xishuangbanna Tropical Botanical Garden, Chinese Academy of Sciences, Mengla, Yunnan, China; ^5^ Southeast Asia Biodiversity Research Institute, Chinese Academy of Sciences, Mengla, Yunnan, China

**Keywords:** *Lindenbergia philippensis*, polyploidization history, karyotype evolutionary trajectories, Lamiales, genome assembly

## Abstract

Lamiales, comprising over 23,755 species across 24 families, stands as a highly diverse and prolific plant group, playing a significant role in the cultivation of horticultural, ornamental, and medicinal plant varieties. Whole-genome duplication (WGD) and its subsequent post-polyploid diploidization (PPD) process represent the most drastic type of karyotype evolution, injecting significant potential for promoting the diversity of this lineage. However, polyploidization histories, as well as genome and subgenome fractionation following WGD events in Lamiales species, are still not well investigated. In this study, we constructed a chromosome-level genome assembly of *Lindenbergia philippensis* (Orobanchaceae) and conducted comparative genomic analyses with 14 other Lamiales species. *L. philippensis* is positioned closest to the parasitic lineage within Orobanchaceae and has a conserved karyotype. Through a combination of Ks analysis and syntenic depth analysis, we reconstructed and validated polyploidization histories of Lamiales species. Our results indicated that *Primulina huaijiensis* underwent three rounds of diploidization events following the γ-WGT event, rather than two rounds as reported. Besides, we reconfirmed that most Lamiales species shared a common diploidization event (L-WGD). Subsequently, we constructed the Lamiales Ancestral Karyotype (LAK), comprising 11 proto-chromosomes, and elucidated its evolutionary trajectory, highlighting the highly flexible reshuffling of the Lamiales paleogenome. We identified biased fractionation of subgenomes following the L-WGD event across eight species, and highlighted the positive impacts of non-WGD genes on gene family expansion. This study provides novel genomic resources and insights into polyploidy and karyotype remodeling of Lamiales species, essential for advancing our understanding of species diversification and genome evolution.

## Introduction

Whole-genome duplication (WGD) or polyploidization is a prevalent process in terrestrial plants, contributing to genetic diversity, particularly in ferns and angiosperms (Julca et al., 2018; Kubis et al., 1998; Jurka, 2000; Korf, 2004; Majoros et al., 2004; Li and Durbin, 2009; Katoh and Standley, 2013; Kellogg, 2016; Li et al., 2016; Kalyaanamoorthy et al., 2017; Landis et al., 2018; Luo et al., 2018; Mandáková and Lysak, 2018; Li et al., 2019; Lovell et al., 2021; Kong et al., 2023; Liao et al., 2023; Liu et al., 2023; Letunic and Bork, 2024). An increasing number of WGD events have been identified across various lineages from whole-genomic sequencing and comparative genomic analyses (Cui et al., 2006; Soltis et al., 2009; Jiao et al., 2011; Vanneste et al., 2014; Van de Peer et al., 2017). WGD events generally arise through two primary mechanisms: autopolyploidization, involving whole-genome duplication within a single species, and allopolyploidization, resulting from the hybridization of two distinct species (Stebbins, 1947; Cheng et al., 2018). WGD events can provide their ancestors with a ‘genomic playground’, enabling new mutations to arise and tend to be fixed (through gene sub-functionalization and/or neofunctionalization). Consequently, these may contribute to physiological and morphological innovations, making WGD events as a significant driving force for species diversification and environmental adaptation (Cheng et al., 2018; Ren et al., 2018).

WGD events play important roles in promoting angiosperm diversification. However, whether these events are correlated with higher diversification rates remains a subject of debate (Tank et al., 2015; Kellogg, 2016; Landis et al., 2018). The ‘lag phase’ model, positing a delay between polyploidization events and subsequent lineage diversification, offers critical insights into influences of WGD events on species diversification (Dodsworth et al., 2015; Tank et al., 2015; Clark and Donoghue, 2017; Mandáková and Lysak, 2018). In other words, the WGD event likely initiated many speciation events across angiosperm lineages and also provided the genetic basis for the post-polyploid diploidization (PPD) process (Mandáková and Lysak, 2018). PPD process is different from WGD events by involving a process of karyotype evolutionary trajectories, which primarily includes changes in genome size, chromosomal rearrangements (alterations in chromosomal number and structure), subgenome-specific fractionation (including biased gene retention/loss and gene sub-/neofunctionalization), differential expression of homologous genes, activation of transposable elements (TE), and epigenetic reprogramming (Paterson et al., 2004; Wang et al., 2005; Mandáková and Lysak, 2018; Zhuang et al., 2019). Therefore, the PPD process may also play a significant role in promoting the diversification rate of angiosperms.

The evolutionary mechanism and significance of promoting species diversity through the PPD process have been elucidated and reviewed by several studies (Mandáková et al., 2017; Mandáková and Lysak, 2018; Mayrose and Lysak, 2021). Generally, dysploid or non-dysploid changes in chromosome number and the fractionation of duplicated genes represent the primary aspects of the PPD process. Among them, chromosomal changes arising from dysploid alterations can radically increase or decrease the base number of chromosomes. Both descending and ascending dysploidyies are significant in karyotype evolution, with the latter primarily observed in a few plant groups possessing monocentric chromosomes, such as the cycad genus *Zamia* (Rastogi and Ohri, 2019; Mayrose and Lysak, 2021). The evolution of land plant chromosomes is predominantly characterized by descending dysploidy (Carta et al., 2020; Mayrose and Lysak, 2021; Wang et al., 2022c; Kong et al., 2023). Centric fission is traditionally considered the most common form of ascending dysploidy (Birchler and Han, 2018). Unlike ascending dysploidy, descending dysploidy can be initiated by two mechanisms, including end-to-end joining (EEJ) and nested chromosome fusion (NCF) (Morin et al., 2017; Ren et al., 2019; Sun et al., 2022). In general, chromosomal diploidization can also be accompanied by various non-dysploid chromosomal rearrangements (CRs), such as inversions, reciprocal translocations, deletions, and duplications (Schubert and Lysak, 2011; Sun et al., 2022). With the alterations in dysploidy and non-dysploidy, the karyotype of specific lineages will undergo significant reshuffling, leading to karyotype modifications and potentially initiating interspecific reproductive barriers. Consequently, these processes may enable some species to acquire evolutionarily advantageous genetic diversity, thus adapting to a changing environment (Soltis et al., 2009; Clark and Donoghue, 2017).

In addition to dysploid or non-dysploid changes, the prevalence of dominant subgenomes, resulting from the preferential retention of genes, is notable in many lineages that have undergone WGD events (Edger et al., 2017; Lovell et al., 2021; Wang et al., 2022b). Consequently, compared to a submissive subgenome, a dominant subgenome often retains more ancestral genes, exhibits higher levels of homologous gene expression, and undergoes stronger purifying selection (Sun et al., 2023). The biased retention (fractionation) of redundant genes resulting from WGD events may facilitate the adaptation of lineage-specific species to diverse ecological environments during speciation. Wu et al. (2020), for example, investigated gene duplicates across 25 genomes, revealing that duplicates retained following WGD events often correlate with environmental adaptability. Specifically, gene families associated with cold and dark conditions were frequently preserved in several lineages following WGD events around the Cretaceous-Paleogene boundary, a period marked by significant global cooling and darkness. Benefiting from karyotype changes, lineage-specific species evolve towards advantageous genetic diversity through the PPD process. This evolutionary advantage provides them with greater buffering capacity against mutations than their ancestors, thereby aiding speciation and enhancing adaptability in harsh environments (Comai, 2005; Ren et al., 2018; Clo, 2022). However, elucidating the complex process of PPD is challenging because, in most species, the ancestral chromosome tend to scatter and fragment within the new karyotype due to changes following the long evolutionary history (Damas et al., 2018; Ren et al., 2019; Zhao et al., 2021). Consequently, the intricate process of PPD, which involves a range of evolutionary modifications, remains a largely overlooked and understudied topic, particularly in certain specific lineages.

Representing one of the most abundant and diverse plant groups, the order Lamiales comprises over 23,755 species and 24 families (https://www.britannica.com/plant/Lamiales). These plants play a crucial role in providing a wide variety of horticultural, ornamental, and medicinal species. Besides, a variety of ecotype plants can be found in this lineage, including autotrophic and heterotrophic (parasitic and carnivorous) plants, aquatic and terrestrial plants. The high species diversity in Lamiales can be directly reflected in the abundant genetic materials. More importantly, almost all Lamiales species shared a common WGD event (the *L event*), and most retain a relatively complete ancestral karyotype, making them as ideal resources for investigating the PPD process (Feng et al., 2020).

The history of polyploidization and the PPD process have long been subjects of extensive study due to their significant roles in species adaptation and evolution. However, research across many lineages has been limited by a lack of comprehensive genomic resources. Encouragingly, the increasing availability of chromosomal-level genome assemblies is now enabling more detailed investigations into the history of polyploidization and the evolutionary trajectories of karyotypes within specific lineages. Significant advances have been made in some specific lineages such as Asteraceae (Kong et al., 2023), Cucurbitaceae (Wang et al., 2022a), and Nyssaceae (Feng et al., 2024). *L. philippensis* is part of Orobanchaceae in Lamiales with a unique taxonomic status, being closest to the parasitic lineage within Orobanchaceae (Li et al., 2019; Mutuku et al., 2021). Besides, *L. philippensis* exhibited a conserved karyotype according to our previously exploration. In this study, to provide more insightful information about the polyploidization history, karyotype evolutionary trajectories, and the subgenomes evolutionary traits in the Lamiales, we assembled a chromosome-level genome of *L. philippensis* using Oxford Nanopore Technology (ONT) sequencing, Illumina sequencing, and high-throughput chromosome conformation capture (Hi-C) technology. Furthermore, we conducted a comparative genomic analysis on *L. philippensis* and other 14 genomes from 12 families within the order Lamiales, with *Vitis vinifera* and *Ophiorrhiza pumila* as outgroup references. The polyploidization histories of most Lamiales genomes were validated and corrected through combined Ks and syntenic depth analyses. Additionally, an ancestral karyotype of Lamiales species was constructed, and its evolutionary trajectories were deciphered in eight Lamiales species. Our study provides valuable genomic resources and will facilitate further research into genome evolution and the PPD process in Lamiales.

## Materials and methods

### Plant materials and DNA extraction

The plant samples of *L. philippensis* were collected from the same adult plant cultivated at Xishuangbanna Tropical Botanical Garden, Chinese Academy of Sciences, and identified by Professor Wen-Bin Yu. Fresh leaves were stored in liquid nitrogen and sent to Novogene Co., Ltd. for sequencing (Beijing, China). The high-quality genomic DNA of *L. philippensis* was prepared by a modified CTAB method (Karoonuthaisiri et al., 2020) and purified with QIAGEN^®^ Genomic kit (QIAGEN, USA) at Novogene Co., Ltd. (Beijing, China). The quality and quantity of the extracted genomic DNA were assessed using a NanoDrop 2000 spectrophotometer (NanoDrop Technologies, Wilmington, DE, USA), Qubit dsDNA HS Assay Kit on a Qubit 3.0 fluorometer (Life Technologies, Carlsbad, CA, USA) and electrophoresis on a 0.8% agarose gel, respectively.

### Long read sequencing

For long-read sequencing, a total of 2 μg DNA was used for the ONT library construction. After the sample was qualified, long DNA fragments are selected using the BluePippin system (Sage Science, Beverly, MA, USA). Further, the ends of DNA fragments were repaired and a ligation reaction was conducted using the NEBNext^®^ Ultra™ II End Repair/dA-Tailing Module Kit. The ONT library with an insert size of 30 kb was prepared using the ligation sequencing kit 1D (SQKLSK109; Oxford Nanopore Technologies, Oxford, UK) according to the manufacturer’s instructions. The ONT sequencing was then performed on an Oxford Nanopore PromethION 48 platform at Novogene Co., Ltd. (Beijing, China).

### Illumina short read sequencing

In total, 1 μg DNA was used as the input material and sequencing library was generated using the VAHTS Universal DNA Library Prep Kit for MGI (Vazyme, Nanjing, China). Following the manufacturer’s recommendations, and index codes were added to attribute sequences to sample. The Library quantification and size were measured using Qubit 3.0 Fluorometer (Life Technologies, Carlsbad, CA, USA) and Bioanalyzer 2100 system (Agilent Technologies, CA, USA). A paired-end library was created with a 350 bp insert size using the GenElute Plant Genomic DNA Miniprep kits following the manufacturer’s instructions (Sigma-Aldrich, Corp., St. Louis, MO, USA). Subsequently, the short-read library was performed on the Illumina NovaSeq 6000 platform (Illumina Inc., San Diego, CA, USA).

### Hi-C library construction and sequencing

The Hi-C libraries were constructed following established protocols (Padmarasu et al., 2019). Initially, samples were cross-linked under vacuum infiltration using formaldehyde. Subsequently, the cross-linked samples were subsequently digested using *DpnII*. After reversing cross-links, the ligated DNA was extracted using the QIAamp DNA Mini Kit (Qiagen) according to the manufacture’s instruction. Purified DNA was then sheared to 300 bp to 500 bp fragments, which underwent blunt-end repair, A-tailing, and adaptor addition. The resulting fragments were purified through biotin-streptavidin-mediated pull-down and subjected to PCR amplification. Finally, the Hi-C libraries were quantified and sequenced on the Illumina NovaSeq 6000 platform (Illumina Inc., San Diego, CA, USA).

### Genome assembly and quality evaluation

Prior to conducting the assembly, it is imperative to conduct a comprehensive survey of the genomic features. To accomplish this, we utilized clean paired-end short reads and employed GenomeScope (v2.0) and Jellyfish (v2.2.10) with default parameters to assess the genome size, heterozygosity, and repeat content of the *L. philippensis* genome (Marçais and Kingsford, 2011; Ranallo-Benavidez et al., 2020). Furthermore, flow cytometry (BD FACSCalibur) was also used to investigate the genome size. For the genome assembly, we initially assembled the clean long reads to generate the draft assembly using NextDenovo (v2.4.0) with the following parameters “task = all; rerun = 3; read_cuoff = 1k; seed_cutoff = 8k; seed_cuoff = 8k; genome_size = 400 m;seed_cutfiles = 80; blocksize = 10g; pa_correction = 80; minimap2_options_raw = -x ava-ont -t 16; sort_options = -m 10g -t 16 -k 50; correction_options = -p 32 random_round = 100 minimap2_options_cns = -x ava-ont -t 20 -k17 -w17; nextgraph_options = -a 1”. Subsequently, the draft assembly underwent three rounds of polishing using NextPolish (v1.3.1) with the following parameters “rerun = 3; parallel_jobs = 8; multithread_jobs = 8; sgs_options = -max_depth 100 -bwa”. To obtain a preliminary genome assembly, haplotyped duplication sequences were filtered using Redundans (v1.01) with parameters “ident=0.95, ovl=0.95” (Pryszcz and Gabaldón, 2016). For scaffolding contigs, Hi-C data were mapped to the *L. philippensis* preliminary assembly using Juicer (v1.6.2) with parameters of “-s *DpnII* -t 40” (Durand et al., 2016). Subsequently, the valid reads were utilized to order and orient the contigs by employing 3D-DNA (Dudchenko et al., 2017). Any missing joins were rectified based on the Hi-C contact signals using Juicebox (v1.11.08) (https://github.com/aidenlab/Juicebox). The completeness of the genome assembly was evaluated using BUSCO (v5.1.2) with “eukaryota_odb10” dataset downloaded from the BUSCO website (https://busco-archive.ezlab.org/v3/) (Seppey et al., 2019). We utilized BWA-MEM (v0.7.12) (Li and Durbin, 2009) for mapping Illumina reads to the assembly and computed mapping statistics with SAMtools (v1.9) using the “flagstat” module (Danecek et al., 2021).

For transcriptome assembly, we downloaded the raw reads of RNA sequencing data from NCBI (ERR2040586, ERR2040587) and used Fastp (v0.20.1) to filter the low quality reads with the following parameters “-q 30 -u 40 -l 50 -w 16”. Trinity (v2.11.0) with the following parameters of “–seqType fq –JM 300G –CPU 20” was used to perform the transcriptome *de novo* assembly (Grabherr et al., 2011).

### Genome annotation

Repetitive elements (REs) across all 17 species were predicted through a combination of evidence-based and *ab initio* methods. For the evidence-based method, we predicted repeats within the target genome using RepeatMasker with the following parameters “-a -nolow -no_is -norna” and RepeatProteinMask with parameters of “-engine ncbi -noLowSimple -pvalue 0.0001” (vopen-4.0.9) (Chen, 2004) based on the Repbase (v24.06) (Jurka, 2000). For the *ab initio* method, we first constructed a *de novo* repeat library of the target genome using RepeatModeler (v2.0) with the parameter “-engine rmblast”. Long terminal retrotransposons (LTRs) were identified using both LTR_FINDER_parallel (v1.1) (Ou and Jiang, 2019) with the following parameters “-harvest_out -size 1000000 -time 300 -finder” and LTRharvest v1.0 (Ellinghaus et al., 2008) with the following parameters “-minlenltr 100 -maxlenltr 7000 -mintsd 4 -maxtsd 6 -motif TGCA -motifmis 1 -similar 85 -vic 10 -seed 20 -seqids yes”. Then, the LTRs candidates were further passed to LTR_retriever (v2.8) (Ou and Jiang, 2018) with default parameters to filter out false LTRs, and calculate the LTR Assembly Index (LAI). Finally, the repeat libraries from LTR_retriever and RepeatModeler were merged to complete *de novo* prediction of REs using RepeatMasker with the following parameters “-nolow -no_is -norna”. In addition, tandem repeats were predicted by using the Tandem Repeat Finder (TRF v4.09) package (Benson, 1999) with the following parameters “2 7 7 80 10 50 2000 -d -h”.

The prediction of protein-coding genes in the *L. philippensis* genome involved the integration of three distinct methods, including *ab initio* gene prediction, homology-based gene prediction, and RNA-Seq-assisted gene prediction. Before proceeding with protein-coding gene prediction, we soft-masked the assembled *L. philippensis* genome using Bedtools (Quinlan and Hall, 2010) according to the annotated file of TEs. For *ab initio* gene prediction, we employed GenScan (v1.0) (Aggarwal and Ramaswamy, 2002), GlimmerHMM (v3.0.3) (Majoros et al., 2004), Augustus (v3.2.2) (Stanke et al., 2008), and SNAP (v1.0) (Korf, 2004) to predict protein-coding genes. Next, homology-based gene prediction was performed using TBLASTN (Altschul et al., 1990) with a cutoff threshold of 1e^-5^, searching against protein sequences from five reference species, including *A. thaliana*, *V. vinifera*, *Solanum lycopersicum*, *S. indicum*. To execute RNA-Seq-assisted gene prediction, the transcriptome assembly was used for gene prediction by comparing it with genomes using the Program to Assemble Spliced Alignments (PASA) (Haas et al., 2003). Finally, a non-redundant gene set was integrated using EvidenceModeler (v1.1.1) (Haas et al., 2008) and updated with PASA. Based on sequence similarity and domain conservation, functional annotations of gene models were predicted by the online EggNOG (v5.0.0) database (Cantalapiedra et al., 2021).

### Phylogenetic reconstruction and comparative genomics analysis

The longest protein-coding sequences of *L. philippensis* and the other 16 species were extracted and clustered using OrthoFinder (v2.5.2) (Emms and Kelly, 2019). Subsequently, the protein-coding sequences of single-copy gene were subjected to multiple sequence homology alignment using Mafft (v7.471) (Katoh and Standley, 2013) with the following parameters “–localpair –maxiterate 1000”. Each coding sequence (CDS) was aligned separately according to the corresponding amino acid alignments using PAL2NAL (v14) (Suyama et al., 2006), and then all CDS matrixes were concatenated into a supermatrix. After filtering the poorly aligned regions of integrated CDS alignments using Gblocks (v0.91b) (Castresana, 2000), a maximum likelihood (ML) tree was constructed using IQ-TREE v2.2.0.3 (Kalyaanamoorthy et al., 2017) with the following parameters “-m MFP; -bb 1000; -nt 10” and with the best-fit model (GTR+F+I+G4). Divergence times for single-copy gene supermatrix dataset were estimated based on the ML tree using MCMCTree module from the PAML package with the following parameters “burnin = 50000; nsample = 100000” (Yang, 2007). Two fossil calibration points for divergence time estimation were searched from the TimeTree database (http://www.timetree.org/). One is *L. philippensis* versus *V. vinifera* (range: 111.4~123.9 Mya) and another is *L. philippensis* versus *B. alternifolia* (range: 31.5~56.1 Mya). The resulting phylogenetic tree was visualized using FigTree (v1.4.3) (https://github.com/rambaut/figtree). The expansion and contraction of gene family in *L. philippensis* were determined using Computational Analysis of Gene Family Evolution (CAFE v5.0) (Mendes et al., 2021) with the following parameters “-k 3 –cores 30”. This process through comparing orthologs groups of itself with other 16 species based on the cluster results of OrthoFinder (v2.5.2) (Emms and Kelly, 2019) and the ultrametric phylogeny generated from r8s (Mulcahy et al., 2012). Finally, ortholog groups with *P* < 0.05 were considered as gene families undergoing significant expansion or contraction. The correlation between genome size and repeat content was calculated using the “cor.test” function in R 4.2.1 with the Pearson method.

### Analyses of whole-genome duplication

The WGD events experienced by *L. philippensis* and the other 16 species were determined by combining the analysis of synonymous substitutions per synonymous site (Ks) and the syntenic analysis that reflects the syntenic depth of intergenomic collinear blocks.

Firstly, syntenic blocks (paralogous genes) within each species were identified using WGDI (v0.6.2) (Sun et al., 2022) with the parameters “-d, -icl, -ks, -bi, -c, -bk”, and then the Ks between collinear genes were calculated by using the Nei–Gojobori approach as implemented in the PAML (v.4.9h) package (Yang, 2007). Median Ks values were used to represent each syntenic block, and Ks peak fitting was performed using WGDI with the “-pf” option (Sun et al., 2022). Secondly, the syntenic depths of collinear genes within other species were employed to determine syntenic ratios between different species, confirming their polyploidy levels. To exactly detect the polyploidization levels, we detected the syntenic depth via two methods. One method involved using WGDI (Sun et al., 2022) with the “-bk” option, while the other one utilized JCVI (v1.3.8) with two sets of parameters: “jcvi.compara.catalog ortholog; –no_strip_names –cscore=0.99” and “jcvi.compara.synteny depth –histogram” (Tang et al., 2008).

### Inference of Lamiales ancestral karyotype and analyses of karyotype evolutionary trajectory

We used the ‘Telomere-centric genome repatterning model’ proposed in previous study (Wang et al., 2015; Sun et al., 2022) to construct the LAK and infer its evolutionary trajectory in Lamiales plants. Given the conserved karyotype of *L. philippensis*, its genome was chosen to complete the construction of LAK. The construction process was delineated into three key steps: Step 1 entailed the detection of Whole Genome Duplication (WGD); Step 2 involved the reconstruction of the ancestral karyotype; and Step 3 focused on validating the accuracy of the reconstructed ancestral karyotype. A more detailed description was provided in **Supplementary Materials** (**Note 1**).

To analyze the evolutionary history of karyotype among Lamiales species, 13 species from eight families were chosen. Similar to the process of LAK inference, we utilized WGDI (Sun et al., 2022) with the parameters “-d, -icl, -bi, -c, -km, -d” to complete karyotype mapping between different species with LAK. Additionally, the dynamic evolutionary trajectory of LAK and post-LAK following the γ-WGT event was illustrated using Adobe Animate software.

### Construction of subgenome and comparative analyses of eight Lamiales species

Eight species, each representing a distinct family and possessing a relatively complete ancestral karyotype, were selected to investigate the traits of karyotype evolutionary trajectory. To precisely build the sub-genomes, two LAK copies in *L. philippensis* (post-LAK1-22) were created to aid in constructing the sub-genome of other species. Similar to the previous reconstruction of LAK, we utilized WGDI with the parameters “-d, -icl, -bi, -c, -km, -ak, -d” to construct the sub-genomes of the eight species. The syntenic relationship between the 16 subgenomes was then visualized using JCVI (Tang et al., 2008). For further characterization of the subgenome, each subgenome was tackled as species, and their corresponding protein-coding sequences were clustered into orthogroups using OrthoFinder (v2.5.2) (Emms and Kelly, 2019). The intersection of different groups was visualized using a website tool at https://bioinformatics.psb.ugent.be/webtools/Venn/.

### The identification of different modes of gene duplication and the analysis of CYP superfamily

Various gene duplication modes were identified utilizing the “DupGen_finder-unique.pl” module of DupGen_finder (Qiao et al., 2019) with default parameters, and *O. pumila* was set as the reference. We identified CYP genes using HMMER v3.3.2 (Potter et al., 2018) with parameter ‘–cut_tc’. The Pfam HMM models, namely PF00067 was set as queries for the identification of CYPs. The previously characterized *A. thaliana* CYPs genes was downloaded from http://p450.kvl.dk/index.shtml and used as outgroups. To construct the phylogenies for CYPs, the protein sequences were aligned using MAFFT (Katoh and Standley, 2013). The poor alignments were trimmed using trimAl (Capella-Gutiérrez et al., 2009). ML phylogenetic trees were constructed with IQ-TREE (Kalyaanamoorthy et al., 2017) and visualized using iTOL (Letunic and Bork, 2024).

## Results

### Genome assembly and annotation of *Lindenbergia philippensis*

**Figure 1:**
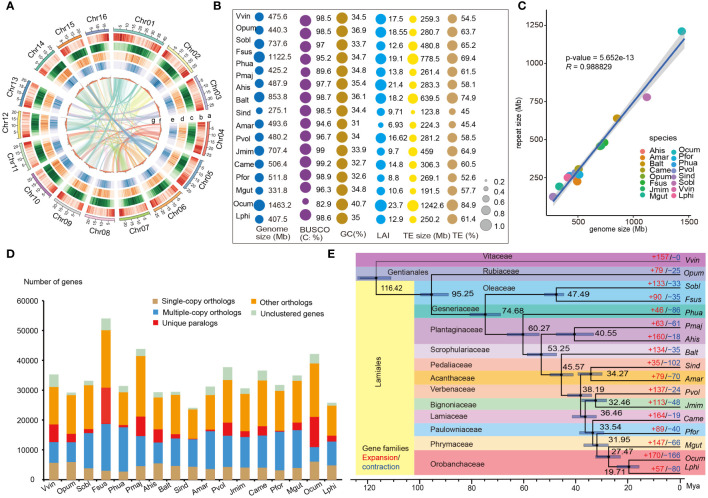
Genomic features and comparative analysis of *L. philippensis* with other 16 species. **(A)** The genomic features are arranged in the order of pseudo-chromosomes (scale is in Mb), gene density, repeat density, LTR/Gypsy, LTR/Copia, GC contents, and syntenic blocks from outside to inside in 300 kb intervals across the 16 pseudo-chromosomes. **(B)** Comparative analysis of genomic quality index in *L. philippensis* (Lphi) with other 16 species, *P. huaijiensis* (Phua), (*F*) *suspense* (Fsus), *S. oblate* (Sobl), *A hispanicum* (Ahis), *P. major* (Pmaj), *C.alternifolia* (Balt), *S. indicum* (Sind), *A. marina* (Amar), *P. volubilis* (Pvol), (*J*) *mimosifolia* (Jmim), *C. americana* (Came), *P. fortune* (Pfor), *M. guttatus* (Mgut), *O. cumana* (Ocum), *V. vinifera* (Vvin) and *O. pumila* (Opum). The size of the colored round shapes represents the number or proportions of all indexes in each species. **(C)** Analysis of the correlation between genome size and RE content among 17 species. **(D)** Distribution of single- and multiple-copy, and other orthologs, unique paralogs, and unclustered orthologs per species from orthogroup clustering by OrthoFinder (v2.5.2) (Emms and Kelly, 2019). **(E)** Phylogenetic tree inferred from single-copy orthologs among selected species. Black numbers in each node denote the divergence time of each clade (Mya), and gray bars are 95% confidence intervals for the time of divergence between different clades. The red and the blue numbers at the terminal branches show the expansion (red) and contraction (blue) of gene families for each species.

Through the analysis of 17-kmer frequencies from Illumina short-reads and flow cytometry, the genome size of *L. philippensis* was estimated at approximately 416.78 Mb and 396.66 Mb, respectively, with a heterozygosity rate of 0.706% (**Supplementary Tables S1**, **S2**; **Supplementary Figures S1**, **S2**). The consistency of genome size estimation was observed between these two methods. A total of 40.13 Gb (101×) of raw ONT long-reads were utilized for the initial assembly of contigs using NextDenovo (v2.5) (https://github.com/Nextomics/NextDenovo) (**Supplementary Table S3**). After two rounds of polish of the 80.49 Gb (202×) Illumina short-reads using Nextpolish v1.2.1 (https://github.com/Nextomics/NextPolish), we obtained 949 final contigs with a total size of 406.79 Mb and a N50 of 1.79 Mb (**Supplementary Tables S3**, **S4**). Subsequently, the polished contigs were clustered and ordered using 131.51 Gb (331×) Hi-C data through Juicer (Durand et al., 2016) and 3D-DNA (Dudchenko et al., 2017), resulting in the successful construction of 16 pseudo-chromosomes with a scaffold N50 of 23.51 Mb, covering approximately 96.55% of the final assembled sequences (393.39 Mb/407.46 Mb) (**Figure 1A**; **Supplementary Figure S3**).

The final assembled genome size was nearly close to the size estimated by the flow cytometry and the 17 kmer frequency distribution (**Supplementary Figure S1**; **Supplementary Table S4**). Furthermore, mapping 536,633,198 Illumina reads to the final assembly resulted in a mapping rate of 99.15% and a coverage rate of 95.05% (**Supplementary Table S5**). The completeness of genome assembly is 98.6% of BUSCO genes based on the embryophyta_10 dataset (Seppey et al., 2019), which was comparable with 14 genomes of Lamiales species (**Figure 1B**; **Supplementary Table S6**). *Lindenbergia philippensis* genome had a high Long-terminal repeat (LTR) Assembly Index (LAI) score of 12.9 (**Figure 1B**), meeting the “reference standard” (LAI value > 10) of genome assembly proposed by Finn et al. (2011).

Based on homologous and *de novo* prediction, 250.16 Mb of repetitive elements (REs) were identified in the *L. philippensis* genome, constituting 61.40% of the assembly genome. These elements included LTRs (39.68%), DNA transposons (6.63%), LINEs (0.86%), SINEs (0.02%), and unclassified sequences (15.78%) (**Supplementary Table S8**). After masking the REs, 25,693 protein-coding genes were identified by combining *de novo*, homology-based, and RNA-Seq-based predictions. On average, each predicted gene had an average length of 3,800 bp and contained five exons with an average length of 232 bp (**Supplementary Table S9**). Approximately 94.89% of protein-coding genes were functionally annotated by existing databases (**Supplementary Table S10**).

### Comparative and evolutionary genomics of *Lindenbergia philippensis* and its relatives

To investigate genomic characteristics of *L. philippensis* and its relatives, comparative genomic analyses were performed on 15 representative genomes from 12 families of Lamiales and two outgroups, *V. vinifera* and *O. pumila* (**Figure 1E**, **Supplementary Table S7**). The annotation and comparison of their REs revealed that the repeat size was widely distributed in these 17 genomes, varying from 88.8 Mb to 1,242.6 Mb, with *O. cumana* exhibiting the highest repeat content (**Figure 1B**; **Supplementary Table S8**). Meanwhile, correlation analysis showed that the genome size was positively correlated with the repeat contents (*R* = 0.97, *P* < 0.05), which was consistent with previous studies (**Figure 1C**) (Shao et al., 2019; de Lima and Ruiz-Ruano, 2022).

By employing OrthoFinder (v2.5.2) (Emms and Kelly, 2019) to cluster orthologs, a total of 576,537 genes from 17 genomes were classified into 540,506 orthologs groups and 36,031 unclustered genes. Among them, 7,775 groups were shared by all 17 species, including 326 single-copy orthologs groups (**Figure 1D**; **Supplementary Table S11**). *Lindenbergia philippensis* possessed 1,934 species-specific genes, including 289 orthologs genes and 628 unclustered genes (**Figure 1D**; **Supplementary Table S12**). The biological processes of species-specific genes were mainly distributed in ‘host cellular response’, ‘metabolic process’ and ‘biosynthetic process’ (**Supplementary Figure S4**), suggesting the evolution of key enzyme genes associated with metabolite synthesis and pathways for environmental adaptation in *L. philippensis*. The phylogenetic tree constructed using 326 conserved single-copy genes from 17 genomes using the maximum-likelihood method showed that *L. philippensis* was sister to parasitic species in Orobanchaceae, aligning with prior research (**Figure 1E**) (Mutuku et al., 2021). Divergence time estimation showed that the divergence between *L. philippensis* and *O. cumana* occurred at ~19.71 million years ago (Mya), and the Lamiales diverged from the Gentianales at ~95.25 Mya (**Figure 1E**). Expansion or contraction of gene families is often associated with adaptive divergence in closely related species (Cheng et al., 2017). Therefore, we investigated changes in gene families using the estimated phylogeny to capture key genomic information associated with *L. philippensis* adaptability. Compared to related species, a total of 57 gene families (including 496 genes) and 80 gene families (including 90 genes) exhibited significant expansion and contraction in the *L. philippensis* genome, respectively (*P* < 0.05) (**Figure 1E**). Interestingly, the expanded genes were primarily enriched in many secondary metabolite biosynthetic pathways (e.g., flavonoid biosynthesis and metabolic process, glucan metabolic process and cellulose biosynthetic process), suggesting that *L. philippensis* produces some active substances such as phenols (**Supplementary Figure S5**).

### Polyploidization history of Lamiales species

**Figure 2:**
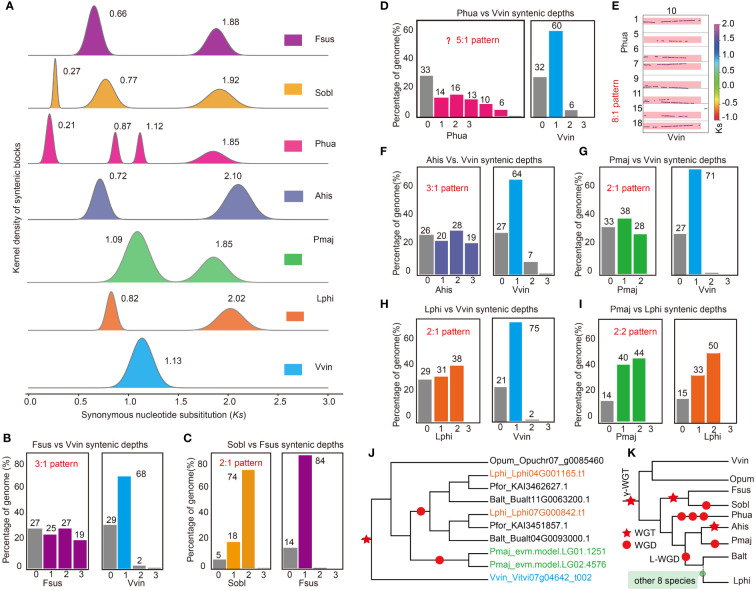
Inference of polyploidization histories in the genomes of the studied Lamiales species. **(A)** The synonymous substitution (Ks) distributions of gene pairs in syntenic blocks among compared genomes. **(B)** The ratio of orthologous genes between *F. suspense* (Fsus) and *V. vinifera* (Vvin). **(C)** The ratio of orthologous genes between *S. oblate* (Sobl) and *F. suspense* (Fsus). **(D)** The ratio of orthologous genes between *P. huaijiensis* (Phua) and Vvin. **(E)** The syntenic depth of homologues blocks between Phua and Vvin. **(F)** Ratio of orthologous genes between *A. hispanicum* (Ahis) and Vvin. **(G)** The ratio of orthologous genes between *P. major* (Pmaj) and Vvin. **(H)** The ratio of orthologous genes between *L. philippensis* (Lphi) and Vvin. **(I)** The ratio of orthologous genes between *P. major* (Pmaj) and Lphi. **(J)** The phylogenetic tree of ten orthologous genes, derived from four Lamiales species, Vvin and *O. pumila* (Opum). **(K)** Overview of WGD events in those 15 Lamiales species. Polyploidization events are indicated by red pentagram (triploidization,WGT) and red round shape (diploidization, WGD).

To unveil the ancient polyploidization history of Lamiales species, we examined the distribution of substitutions per synonymous site (Ks) of intra-genomic collinear blocks in the 15 Lamiales species. Two to four separate peaks were detected in the Ks distribution for species-specific paralogous pairings in those species (**Figure 2A**, **Supplementary Figure S6**), indicating that at least one round of WGD events occurred in this lineage following the γ-WGT event. For example, four obvious Ks peaks were observed in *P. huaijiensis*, reflecting a younger WGD event at Ks 0.21, two distinct WGD events at Ks 0.87 and 1.12 respectively, and γ-WGT event at Ks 1.85. In *S. oblata*, three Ks peaks indicated a younger WGD event at Ks 0.27, a WGD event at Ks 0.77, and the γ-WGT event at Ks 1.92 (**Figure 2A**). Other species such as *F. suspensa*, *A. hispanicum*, *P. major*, *B. alternifolia*, *S. indicum*, *P. volubilis*, *P. fortunei*, *J. mimosifolia*, *C. americana*, *M. guttatus*, *O. cumana* and *L. philippensis* exhibited two peaks (**Figure 2A**; **Supplementary Figure S6**). The first peak indicated a recent WGD event, while the second peak corresponded to the γ-WGT event. *Vitis vinifera* and *O. pumila* displayed only a single peak representing γ-WGT event (**Figure 2A**; **Supplementary Figure S6**). The distribution of Ks peaks showed differences among species (**Figure 2A**; **Supplementary Figure S6**), which was usually caused by evolutionary rate variations in habitat divergence (Sensalari et al., 2022). For example, besides *V. vinifera*, *P. fortunei* exhibited the lowest Ks value in γ-WGT event (**Supplementary Figure S6**), indicating it may have a lower evolutionary rate than other species.

To determine the polyploidization level of Lamiales species after the γ-WGT event, their ratio of orthologous genes with *V. vinifera* was examined with precision. Generally, a species experienced the WGD event will have a corresponding orthologous gene ratio with another species, which only retained their common ancestral karyotype. For instance, Hoang et al. (2023) demonstrated that *Cleome violacea* did not undergo Gg-α (diploidization) event after divergence from a shared ancestor with *Gynandropsis gynandra*, which had experienced a diploidization event. Consequently, *C. violacea* exhibited a 1:2 orthologous gene ratio with *G. gynandra*. Through comparative analysis of genome syntenic blocks, we have successfully determined the level of polyploidization in those 17 species following γ-WGT event. *Ophiorrhiza pumila* exhibited a 1:1 orthologous gene ratio with *V. vinifera* (**Supplementary Figure S7**), suggesting that it underwent only the shared γ-WGT event and did not experience additional WGD events after diverging from their common ancestor. *Forsythia suspensa* had a 3:1 orthologous gene ratio with *V. vinifera* (**Figure 2B**; **Supplementary Figure S8**), indicating it experienced a triploidization at Ks peak 0.66. *Syringa oblata* had a 6:1 orthologous gene ratio with *V. vinifera* and a 2:1 orthologous gene ratio with *F. suspensa*, respectively (**Figure 2C**; **Supplementary Figures S9**, **S10**), indicating it experienced a common triploidization with *F. suspensa* at Ks peak 0.77 and an independent diploidization event at Ks peak 0.27 (**Figure 2A**). The two rounds of WGD events were also proved by previous studies (Julca et al., 2018; Feng et al., 2020). *Primulina huaijiensis* showed an 8:1 orthologous gene ratio with *V. vinifera* (**Figures 2D, E**; **Supplementary Figure S11**). Therefore, the orthologous gene ratio between *P. huaijiensis* and *V. vinifera* could be explained as 2×2×2:1 according to the Ks distribution, corresponding to three rounds of diploidization events rather than two rounds of diploidization events reported in a previous study (Feng et al., 2020). *Antirrhinum hispanicum* had a 3:1 orthologous gene ratio with *V. vinifera* (**Figure 2F**; **Supplementary Figure S12**), indicating that it underwent a triploidization event at Ks peak 0.72 (**Figure 2A**), which was consistent with previous results (Zhu et al., 2023b). *Avicennia marina* had a 4:1 orthologous ratio with *V. vinifera* and a 2:1 orthologous gene ratio with *L. philippensis* (**Supplementary Figures S13**; **S33**), respectively, indicating it experienced a common diploidization with *L. philippensis* at Ks peak 0.77 and an independent diploidization event at Ks peak 0.27 (**Figure 2A**).

In addition, other ten species, including *P. major*, *B. alternifolia*, *S. indicum*, *P. volubilis*, *P. fortunei*, *J. mimosifolia*, *C. americana*, *M. guttatus*, *O. cumana* and *L. philippensis*, had a 2:1 orthologous gene ratio with *V. vinifera* (**Figures 2G, H**; **Supplementary Figures S14**, **S23**). This suggests that they could have experienced a common diploidization event after the γ-WGT event, corresponding to *L_event* revealed by previous results (Julca et al., 2018; Feng et al., 2020). To better determine whether the ten species underwent a common diploidization event, we examined the inter-genomic collinearity relationships among orthologous genes, using *L. philippensis* as the reference. Except for *P. major*, which exhibited a 2:2 orthologous gene ratio with *L. philippensis* (**Figure 2I**; **Supplementary Figure S24**), the remaining eight species displayed a 2:1 orthologous gene ratio with *L. philippensis* (**Supplementary Figures S25**-**S32**). This suggests that *P. major* may have undergone a diploidization event independently, while the remaining nine species shared a common diploidization event after the γ-WGT event. Furthermore, phylogenetic analyses of orthologous genes derived from four paired subgenomes and using *O. pumila* and *V. vinifera* as outgroups, further corroborating this hypothesis (**Figure 2J**).

In summary, after the γ-WGT event, Lamiales species underwent multiple WGD events based on Ks and syntenic analyses. *Syringa oblata* and *F. suspensa* underwent a shared triploidization event, and *S. oblata* subsequently underwent an independent diploidization event, in line with the previous finding (Julca et al., 2018; Feng et al., 2020). *Primulina huaijiensis* experienced three rounds of diploidization events. *Antirrhinum hispanicum* experienced a triploidization event, while *P. major* underwent a diploidization event. Integrating phylogenetic and syntenic analyses, we found that the remaining ten species from nine families, including *B. alternifolia* (Scrophulariaceae), *S. indicum* (Pedaliaceae), *A. marina* (Acanthaceae), *P. volubilis* (Verbenaceae), *J. mimosifolia* (Bignoniaceae), *C. americana* (Lamiaceae), *P. fortunei* (Paulowniaceae), *M. guttatus* (Phrymaceae), *O. cumana*, and *L. philippensis* (Orobanchaceae), underwent a shared diploidization event, known as L-WGD event (**Figure 2K**).

### Construction of Lamiales ancestral karyotype and analyses of karyotype evolutionary trajectories

After polyploidization, substantial karyotype changes frequently occur in many plant genomes. These changes can alter the basic chromosome number and trigger species diversification. In Lamiales, the chromosome numbers of 15 selected species range from 2n=12 to 2n=64 (**Supplementary Table S7**). These variations are primarily caused by karyotype changes. To uncover the karyotype evolutionary trajectories, the *L. philippensis* genome was used to reconstruct the Lamiales ancestral karyotype (LAK).

**Figure 3:**
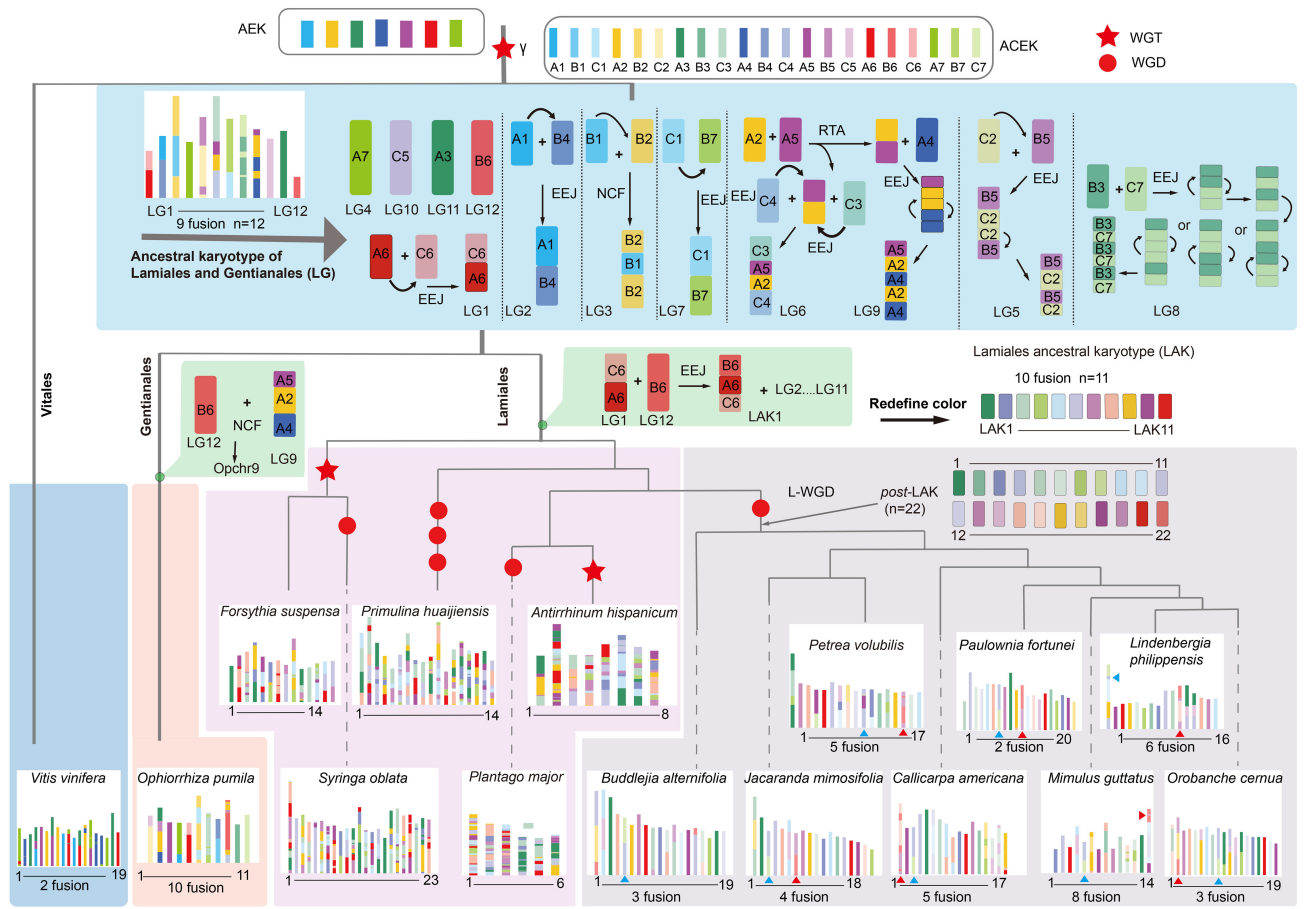
Construction of the Lamiales ancestral karyotype (LAK) and the chromosome evolution trajectories of eight Lamiales species. The topology is the same as in **Figure 1E** and the overview of WGD events was represented by using red pentagram (triploidization, WGT) and red round shape (diploidization, WGD). AEK represents the 7 ancestral eudicot karyotypes. ACEK represents 21 Ancestral core eudicots karyotypes; A, B, and C represent the three AEK produced subgenomes following γ-WGT event. The 11 inferred proto-chromosomes of LAK are represented by LAK1–11. Post-LAK represents two LAK produced subgenomes following the L-WGD event. Different background colors represent different lineages, light blue represents the evolution trajectories of LAK, blue represents Vitales, orange represents Gentianales; light pink and grey represent the experienced or not L-WGD event Lamiales species, respectively. Two distinct EEJ fusion events were marked using blue triangle and red triangle.

To comprehensively delineate the karyotype evolutionary trajectory of the LAK in Lamiales species, we defined the 21 proto-chromosomes of the Ancestral Core Eudicot Karyotypes (ACEK), derived from the triplication of seven ancestral eudicot karyotypes (AEK), as A1-7, B1-7, and C1-7. As a result, a putative LAK was constructed consisting of 11 proto-chromosomes (LAK1-LAK11), which shared the same base chromosomal number with the sister clade species such as *O. pumila* (2n=22), *Morinda officinalis* (2n=22), and *Leptodermis oblonga* (2n=22) from the Gentianales order. This suggests a possible common ancestral karyotype between Lamiales and Gentianales. To validate this hypothesis, we generated a dot plot by comparing the LAK and *O. pumila* (Rubiaceae family) with ACEK. Rubiaceae, positioned at the root of Gentianales, exhibits a higher likelihood of sharing the same karyotype with LAK among its species. The dot plot analysis indicated that nine chromosomes of the LAK and *O. pumila* exhibited a one-to-one correspondence in their collinearity relationship (**Supplementary Figures S34**-**S36**). The primary distinction between them lies in the rearrangement of proto-chromosome B6 (**Figure 3**). Following the methodology used in constructing the LAK, we constructed a hypothetical common ancestral karyotype for Lamiales and Gentianales orders, labeled as LG1-LG12 (**Figure 3**; **Supplementary Figure S37**). In summary, LAK evolved into 11 proto-chromosomes through a series of chromosomal rearrangements, including nine end-to-end joining (EEJ), two nested chromosome fusions (NCF), and ten reciprocal translocations of chromosome arms (RTA). For example, the formation of proto-chromosomes LAK1 was mainly explained by the fusion of A6 and C6 initially with the EEJ pattern and then further fused with B6 through the EEJ pattern (**Figure 3**). Similarly, the evolutionary trajectory of the other ten proto-chromosomes of LAK was inferred in **Figure 3**.

Based on previous results, eight Lamiales species, including *B. alternifolia*, *P. volubilis*, *P. fortunei*, *J. mimosifolia*, *C. americana*, *M. guttatus*, *O. cumana* and *L. philippensis*, have been identified as sharing the L-WGD event. This makes them ideal candidates for exploring the evolutionary characteristics of the LAK. Following the L-WGD event, the LAK underwent duplication, resulting in the formation of 22 proto-chromosomes (post-LAK). To investigate the evolutionary characteristics of the post-LAK in these species, two sets of LAK generated from *L. philippensis* were used to represent post-LAK karyotype and labeled as post-LAK1 to post-LAK22. Two distinct EEJ fusion events were identified by analyzing the dot plot comparing these eight species with the post-LAK (**Figure 3**). The first EEJ fusion event, which involved post-LAK4 and post-LAK8, occurred in all eight species. In contrast, the second EEJ fusion event, involving post-LAK20 and post-LAK22, was only present in seven of these species, with *B. alternifolia* being the sole exception (**Figure 3**). Subsequently, the eight species separated and evolved with different chromosome evolutionary trajectories.

Given the non-dysploid chromosomal changes were prevalent, we focused primarily on depicting the dysploid chromosomal rearrangements in these species. In summary, *B. alternifolia* genome experienced three chromosomal fusions, consisting of two EEJ fusions and one NCF fusion, leading to the current chromosome number n=19 (**Figure 3**; **Supplementary Figure S38**); *J. mimosifolia* genome experienced two EEJ fusions and two NCF fusions to form the current chromosome number n=18 (**Figure 3**; **Supplementary Figure S39**); the *P. volubilis* genome experienced five chromosomal fusions, composing of four EEJ and one NCF, resulting in the current chromosome number n=17 (**Figure 3**; **Supplementary Figure S40**); *C. americana* genome experienced three EEJ fusions, one NCF fusion and one EEJ or NCF fusion, leading to the current chromosome number n=17 (**Figure 3**; **Supplementary Figure S41**); *P. fortunei* genome experienced the fewest karyotype change events to form the current chromosome number n=20, with just two EEJ fusions and no further karyotype evolutionary events (**Figure 3**; **Supplementary Figure S42**); *M. guttatus* genome experienced three EEJ fusions and five NCF fusions to form the current chromosome number n=14 (**Figure 3**; **Supplementary Figure S43**); *O. cumana* genome experienced two EEJ fusions and one NCF fusion to form the current chromosome number n=19 (**Figure 3**; **Supplementary Figure S44**); *L. philippensis* genome experienced two EEJ fusions and four NCF fusions to form the current chromosome number n=16 (**Figure 3**; **Supplementary Figure S45**).

### Comparative analyses of subgenomes in eight Lamiales species

**Figure 4:**
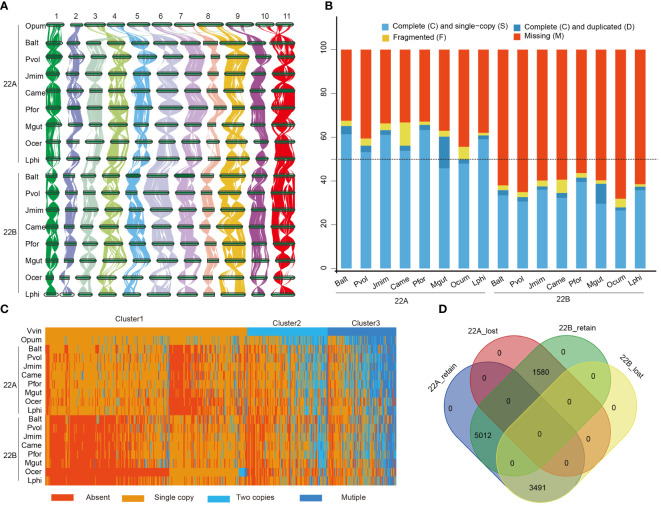
Comparative analysis of subgenomes of 8 species in the Lamiales. **(A)** The synteny plot across *O. pumila* (Opum) genome and sixteen subgenomes, *B. alternifolia* (Balt), *P. volubilis* (Pvol), *J. mimosifolia* (Jmim), *C. americana* (Came), *P. fortune* (Pfor), *M. guttatus* (Mgut), *O. cumana* (Ocum) and *L. philippensis* (Lphi); 22A represents least fractionated subgenome and 22B represents most fractionated subgenome. **(B)** Assessment of Benchmarking Universal Single-Copy Orthologs (BUSCOs) of those sixteen subgenomes with embryophyta_10 (1614) databases. **(C)** Heat map of the clustered copy-number profile matrix in Opum, *V. vinifera* (Vvin), and sixteen subgenomes. Core gene families could be partitioned into four based on the clustering of the copy-number profile data. Rows represent species and columns represent the 10,083 CSOs. Gene families are sorted according to the three different clusters of Vvin. **(D)** Venn diagram showing the distribution of the retained and lost CSO sets.

Following polyploidization, most duplicated genes would experience drastic changes due to the sensitivity of dosage balance (Li et al., 2016). To elucidate the fractionation characteristics of duplicated genes in Lamiales, two sets of post-LAK subgenomes were initially classified as least fractionated (LF, 22A) and most fractionated (MF, 22B) based on their gene counts. Subsequently, 16 subgenomes were constructed using the WGDI, and their grouping was determined based on the collinear relationship with post-LAK. The one-to-one collinear correspondence of these subgenomes with post-LAK confirmed the reliability of these subgenomes (**Figure 4A**; **Supplementary Figures S46**-**S52**), making them suitable for further research. Like the post-LAK, all subgenomes exhibited subgenome dominance. For example, 22A subgenomes (with 14,306 – 24,133 genes) had more gene counts than 22B subgenomes (with 9,364 – 16,727 genes) among these 16 subgenomes (**Supplementary Table S13**). The BUSCO analyses also showed that eight 22A subgenomes had over 50% complete BUSCO genes from the embryophyta_10 dataset, whereas the completeness level in the eight 22B subgenomes was below the threshold of 50% (**Figure 4B**). This phenomenon suggested that these eight species exhibit consistently biased preservation and display a dominance within their respective subgenomes.

To uncover the fractionation pattern of the subgenomes, we utilized OrthoFinder (v2.5.2) (Emms and Kelly, 2019) to group their protein-coding genes into orthogroups, with *V. vinifera* and *O. pumila* as the reference. Stringent criteria were applied to choose representative orthogroups, necessitating orthogroups with a minimum of eight distinct subgenomes, encompassing *V. vinifera* and *O. pumila*. In total, 10,083 orthogroups were selected to comprise the core set of orthogroups (CSOs) for our further analyses. Based on the observed number of gene copies in each CSO, those CSOs were categorized into four distinct types: ‘Absent’ (no gene copies present), ‘Single Copy’ (one gene copy), ‘Two Copies’ (exactly two gene copies), and ‘Multiple’ (more than two gene copies). Furthermore, according to the gene number in *V. vinifera*, those CSOs were organized into three clusters (cluster 1, cluster 2, and cluster 3) (**Figure 4C**). In cluster 1, CSOs mainly consisted of absent or singleton genes in 16 subgenomes (**Supplementary Figure S53**). In cluster 2, CSOs are composed of either absent or present genes in single or two-copy forms. In cluster 3, CSOs mainly consisted of orthogroups that are single, two, or multiple copies. In all three clusters, the number of absent CSOs in the 22B subgenome was significantly greater than that in the 22A subgenome (*P* < 0.05). Conversely, the remaining three types showed the opposite trend, except for the single copy gene in cluster 3 (**Supplementary Figure S53**). Therefore, we speculated that the dominance of the subgenome could primarily originate from a higher frequency of loss and a lower rate of retention. Besides, our results also indicated that the distribution of CSOs across the 22A and 22B subgenome had a nested complementary profile, particularly evident in cluster 1.

We further defined the CSOs present in over 50% of the subgenome as retained CSO sets, while those not maintained are referred to as lost CSO sets. Based on these criteria, slightly over half of the CSO sets (5,071/10,083) displayed a complementary distribution across the two subgenomes, corroborating earlier findings presented in the heat map analysis. Specifically, 3,491 CSOs were conserved exclusively in the 22A subgenome sets, 1,580 CSOs were solely retained in the 22B subgenome sets, and 5,012 CSOs were present in both 22A and 22B subgenomes (**Figure 4D**).

### Different modes of gene duplications driving the dominance of subgenome

**Figure 5:**
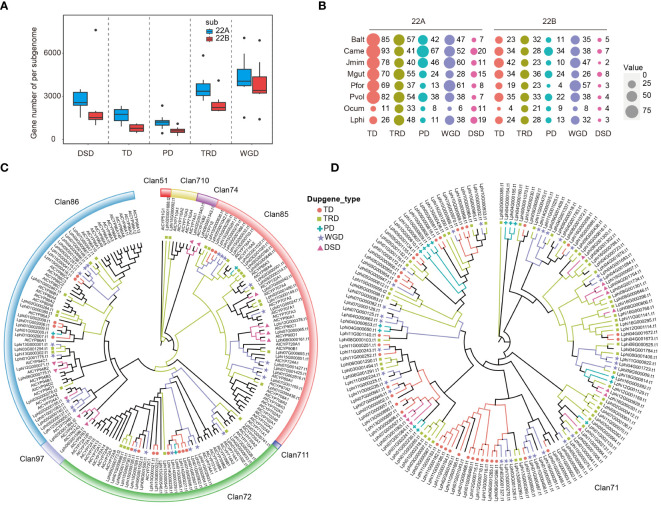
The numbers and the distributions of CYP genes from different gene duplications. **(A)** Distribution of non-WGD (TRD, DSD, TD, PD, and TRD) and WGD genes across two sets of subgenomes in 16 subgenomes. **(B)** The proportions and numbers of CYP genes from different gene duplications are estimated in 16 subgenomes. The size of the colored round shapes represents the number, or proportions of all genes in each gene duplication mode. **(C)** The phylogenetic tree of 8 Clan CYP genes from *L. philippensis* and *A. thaliana*. **(D)** The phylogenetic tree of Clan71 CYP genes from *L. philippensis*.

In addition to WGD events, gene duplication is also a crucial process in expanding the gene family (Fajardo et al., 2023). To determine if gene duplication caused the dominance of subgenome, we conducted statistical analyses on non-WGD (Dispersed duplication, DSD, Tandem duplicate, TD; Proximal duplication, PD; and Transposed duplication, TRD) and WGD genes, as well as on unduplicated genes (UD) within those subgenomes. Our results indicated that all the modes of gene duplications were higher in 22A subgenome sets than in 22B subgenome sets (**Figure 5A**). Cytochrome P450s (CYPs) form the largest enzyme family in plants, representing around 1% of protein-coding genes in various flowering plants (Liu et al., 2023). They can be ideal candidates to study different modes of gene duplications. The distribution of CYP genes in the various modes of gene duplications showed more copies in the 22A subgenome sets than in the 22B subgenome sets across the 16 subgenomes (**Figure 5B**). Interestingly, there are more CYP genes in TD genes than in WGD genes, and the number of CYP genes in TRD and PD was also similar to that in WGD genes, despite their lower total gene count compared to WGD genes (**Figure 5B**). This observation suggests that, besides WGD events, the non-WGD genes also play a crucial role in the expansion of gene families. To detail the influence of the gene duplication on gene family expansion, the phylogenetic tree of CYP genes in *L. philippensis* genome were constructed and with *Arabidopsis thaliana* as references. In total, 242 CYP genes in *L. philippensis* genome were cluster into 9 subfamilies according to the result of Williams et al. (2000) (http://p450.kvl.dk/p450.shtml) (**Figures 5C, D**). Within the phylogenetic tree, the gene duplication modes are distinct among major subfamilies like Clan71, Clan72, Clan85, and Clan86. The remaining subfamilies only exhibit one type of gene duplication mode. Clan71, as the largest subfamily in the CYP superfamily, contains more gene duplication copies of various modes than other subfamilies (**Figure 5D**).

## Discussion

### Genome assembly of *Lindenbergia philippensis* provides an important genomic resource

*Lindenbergia philippensis* belongs to the tribe Lindenbergieae, besides the tribe Rehmannieae, and it is the closest autotrophic sister clade to all parasitic plant lineages in the family Orobanchaceae (Mutuku et al., 2021; Jiang et al., 2022; Xu et al., 2022) (**Figure 1E**). Here, the *L. philippensis* genome was achieved by combining Illumina paired-end sequencing data, Oxford Nanopore data and Hi-C data. The new genome assembly size was 407.46 Mb, close to the estimated size of 396.66 Mb via flow cytometry and 17-kmer frequency estimation (**Supplementary Tables S2**, **S4**). The completeness of the genome assembly was comparable with 15 species in Lamiales (**Supplementary Table S6**). Therefore, the assembly of *L. philippensis* genome had good quality, making it suitable for further analyses. Additionally, the anchored 16 pseudo-chromosomes had good intra-genomic collinear blocks (Note 1), which makes it the high-quality reference genome to deduce the karyotype evolutionary trajectory among relative species. These results provide important genomic resources for further genome study on *L. philippensis* as well as Orobanchaceae in the future.

### Combining Ks and syntenic depth analyses reconstruct the accurate evolutionary history of polyploidization and WGD events

Polyploidization, or WGD events, have been identified as a critical mechanism in facilitating species evolution and diversification across a vast majority of plant lineages (Zhang et al., 2019; Clo, 2022). Additionally, the profound influence of WGD events goes beyond its initial occurrence, and could primarily serve as a catalyst to drive a subsequent PPD process (Soltis and Soltis, 2016; Zhang et al., 2019). However, the PPD process has negative effect on the identification of WGD events and the determination of polyploidization levels.

Currently, although an increasing number of WGD events are being reported through Ks or 4Dtv analyses, syntenic depth analyses, or a combination of these methods, some WGD events are inaccurately determined due to low-quality and limited genomic data and analytical method constraints. For instance, Feng et al. (2020) used Ks analysis to reveal a WGD (the *L even*t) present in almost all Lamiales except the lineage of Oleaceae, which conflicted with the results of Zhu et al. (2023b). Zhu and his colleagues substantiated that the Plantaginaceae underwent a distinct WGD event, diverging from the shared *L event* (Feng et al., 2020; Zhu et al., 2023b). This independent WGD event was confirmed in this study, as well as a recent research by Huang et al. (2023). By combining Ks and inter-species syntenic depth analyses, we validated that *P. huaijiensis* experienced three diploidization events following the γ-WGT event, rather than two WGD events in the previous report (Feng et al., 2020). This discrepancy primarily derived from that Feng et al. (2020) relied on the solely Ks analysis to survey the WGD event, without integrating syntenic depth comparisons across different species. Additionally, two separate Ks values (0.87 and 1.12) (**Figure 2A**) suggested that *P. huaijiensis* underwent two WGD events within a relatively close timeframe. Consequently, these two WGD events could easily be overlooked and misinterpreted as a single event.

WGDI (Sun et al., 2022) and JCVI (Tang et al., 2008) are both popular software options for analyzing WGD events through syntenic depth analysis, but WGDI has advantages over JCVI in distinguishing the level of polyploidization. For example, *O. pumila* and *V. vinifera* had been shown to share the γ-WGT event, the syntenic depths or orthologous gene ratio between them should theoretically be 1:1, ignoring the non-WGD effects, whereas their syntenic depths were determined at 2:2 in the research of Rai et al. (2021) by using JCVI, which cannot identify whether they shared this WGD or not. In our study, the 1:1 orthologous gene ratio of *O. pumila* and *V. vinifera* was validated using WGDI and confirmed that they shared the γ-WGT event, which was aligned with the previous results (Wang et al., 2022c). Besides, the orthologous gene ratio of *P. huaijiensis* compared to *V. vinifera* was showed to be 6:1, corresponding to its three diploidization events. However, their orthologous gene ratio was 5:1 using JCVI, conflicting with its polyploidization history.

Overall, it is imprudent to crudely estimate polyploidization events based solely on the Ks distribution or syntenic depth analysis. While the analysis of Ks can indicate the occurrence of WGD events, it is challenging to clearly distinguish the polyploidization histories. Essentially, Ks analysis only reveals whether the species underwent WGD events, making it hard to ascertain whether the WGD event led to diploidization, triploidization, or other forms of polyploidization. This and previous studies have revealed some misunderstandings regarding the evolutionary history of WGD events, such as the genomic researches of *C. americana* (Hamilton et al., 2020), watermelon (Guo et al., 2013), black pepper (Hu et al., 2019), Olive (Ren et al., 2018), and *Prunus mongolica* (Zhu et al., 2023a). These mistakes significantly increase the chance of misinterpreting the evolutionary history of these events, hindering our comprehensive understandings of the functional evolution of subgenomes, gene families, pathways, and genomic structures. Integrating genomic collinearity analysis with Ks information provides a more accurate and effective method for inferring polyploidization events, as supported by our findings in this study and previous studies (Kong et al., 2023; Sun et al., 2024). Based on this theoretical framework, Sun et al. (2022) have developed an integrated tool WGDI that combines functions for detecting WGD events, analyzing karyotype evolution, and constructing ancestral karyotypes, among other functions, providing an effective and more accurate method for the WGD events analyses. Using this tool, WGD events of 15 species in Lamiales were corrected and validated, providing significant insights for the analysis of WGD events. Moreover, the L-WGD shared by most Lamiales species was validated by combing the Ks and syntenic depth analyses.

### Construction and evolutionary trajectory of ancestral karyotypes in Lamiales

The identification and construction of ancestral karyotypes play a crucial role in confirming the phylogenetic positions of species and elucidating the impact of various polyploidy events on species diversity and evolution (Murat et al., 2017; Kong et al., 2023). The recursive dysploid or non-dysploid changes have reshuffled the ancestral karyotypes of Lamiales, complicating the clear interpretation of polyploidization events (Ren et al., 2018; Feng et al., 2020). In this study, *L. philippensis* was used to construct the LAK, following the theoretical framework that suggested by Sun et al. (2022), consisting of 11 proto-chromosomes. The two complete copies of the paleogenome within the *P. fortunei* genome validated its reliability (**Supplementary Figure S42**).

The evolutionary path analyses of LAK and post-LAK showed that the base number deduction of chromosomes was caused by fusions (Wang et al., 2022c; Feng et al., 2024). This suggested that descending dysploidy may play a major role in karyotype evolution after WGD events, consistent with previous studies indicating that the chromosomal evolution in land plants is mostly characterized by descending dysploidy (Carta et al., 2020; Wang et al., 2022c; Kong et al., 2023). Two distinct EEJ fusion events were detected in those eight species, the first fusion shared by all studied species, while the second fusion event was observed in seven of the eight species, with *B. alternifolia* as the notable exception. This divergence may be a significant factor for its speciation from the other species. This finding also indicates that the PPD process plays a significant role in promoting species diversification. Usually, the reduction of chromosome number critically resulted in the abnormal pairing of gametes, ultimately leading to reproductive isolation (Paliulis and Nicklas, 2000; Luo et al., 2018). Additionally, the eight species showed a lower frequency of non-dysploidy alterations, with dysploidy changes being easily identifiable (**Figure 3**). Interestingly, a higher frequency of EEJ fusion compared to NCF fusion was observed in most species, suggesting that EEJ fusion may have a competitive advantage over NCF fusion in the process of karyotype evolution. While a similar phenomenon was also reported in previous studies (Wang et al., 2022a), the reliability of this advantage is still an understudied topic. The construction of the LAK and the elucidation of its evolutionary trajectory address a significant gap in our understanding of chromosome karyotype evolution within Lamiales. Furthermore, the discovery revealed that the genomes of the eight karyotype-conserved species possess more complete ancestral chromosomal structures, which suggests their potential as model organisms for future genomic research in Lamiales.

### Genomic fractionation and the role of different modes of gene duplications in driving genome evolution

Following polyploidization events, extensive chromosome rearrangements and large-scale gene loss are prevalent due to the dosage balance, particularly in allopolyploids. In this study, following the construction of the post-LAK, we constructed two sets of subgenomes for the eight representative species, respectively. The subgenomes display biased preservation and subgenome dominance, aligning with the lineage-specific hexaploidization seen in *Lupinus* (Xu et al., 2020). This indicated that the L-WGD event may be an allopolyploid event. After observing the fractionation pattern of duplicated genes in these species, we hypothesized that plant species had undergone WGD events that tend to selectively retain these genes within subgenomes in a complementary manner. This suggests that species that underwent WGD events may optimize their genetic repertoire to achieve a more adaptable genetic system in response to changing environments. REs play important roles in driving genome evolution and regulating gene expression (Kubis et al., 1998; Novák et al., 2020; Liao et al., 2023). In this study, we confirmed that the expansion of REs is a key factor influencing genome size variations, which is consistent with some previous studies, and besides polyploidization and gene duplications, repeat expansion was the main factor in amplifying the genome size (Nishihara, 2019; Shao et al., 2019; Novák et al., 2020; de Lima and Ruiz-Ruano, 2022).

Besides, the investigation of different modes of gene duplications across 16 subgenomes revealed that the subgenome 22A exhibited a higher number of duplicate genes than subgenome 22B. This phenomenon shows that gene duplication may play important roles in driving subgenome dominance. Distribution of gene duplication modes across several larger subfamilies in the phylogenetic tree of the *L. philippensis* CYPs superfamily. This diverse distribution also indicates the duplicated gene as a significant force in expanding the gene family (Liu et al., 2023).

## Data availability statement

The raw genome sequencing data of *L. philippensis* are available at the National Genomics Data Center (https://ngdc.cncb.ac.cn/) under BioProject number PRJCA010538 (CRA013614). All data are available from the corresponding author upon request.

## Author contributions

B-ZC: Writing – original draft, Writing – review & editing, Conceptualization, Data curation, Formal analysis, Funding acquisition, Investigation. D-WL: Writing – review & editing, Formal analysis. K-YL: Writing – review & editing. S-TJ: Writing – review & editing. XD: Writing – review & editing. W-BW: Writing – review & editing. X-ZL: Writing – review & editing. T-TH: Writing – review & editing. Y-HL: Writing – review & editing. D-ZG: Writing – review & editing. X-TL: Writing – review & editing. S-CD: Writing – review & editing. Y-FZ: Writing – review & editing. WC: Writing – review & editing. YD: Conceptualization, Data curation, Formal analysis, Funding acquisition, Writing – review & editing. W-BY: Conceptualization, Writing – review & editing.
