# Supplementary material for: Chromosome-level assembly of Lindenbergia philippensis and comparative genomic analyses shed light on genome evolution in Lamiales
Source: Front Plant Sci. 2024 Aug 2;15:1444234. doi: 10.3389/fpls.2024.1444234 (PMC11327160; doi:10.3389/fpls.2024.1444234)
Supplement: Supplementary file 2 [file DataSheet_1.pdf]

Supplementary data Figure1-Figure53

Chromosome-level assembly of *Lindenbergia philippensis* and comparative genomic analyses shed light on genome evolution in Lamiales

Bao-Zheng Chen *et al.*

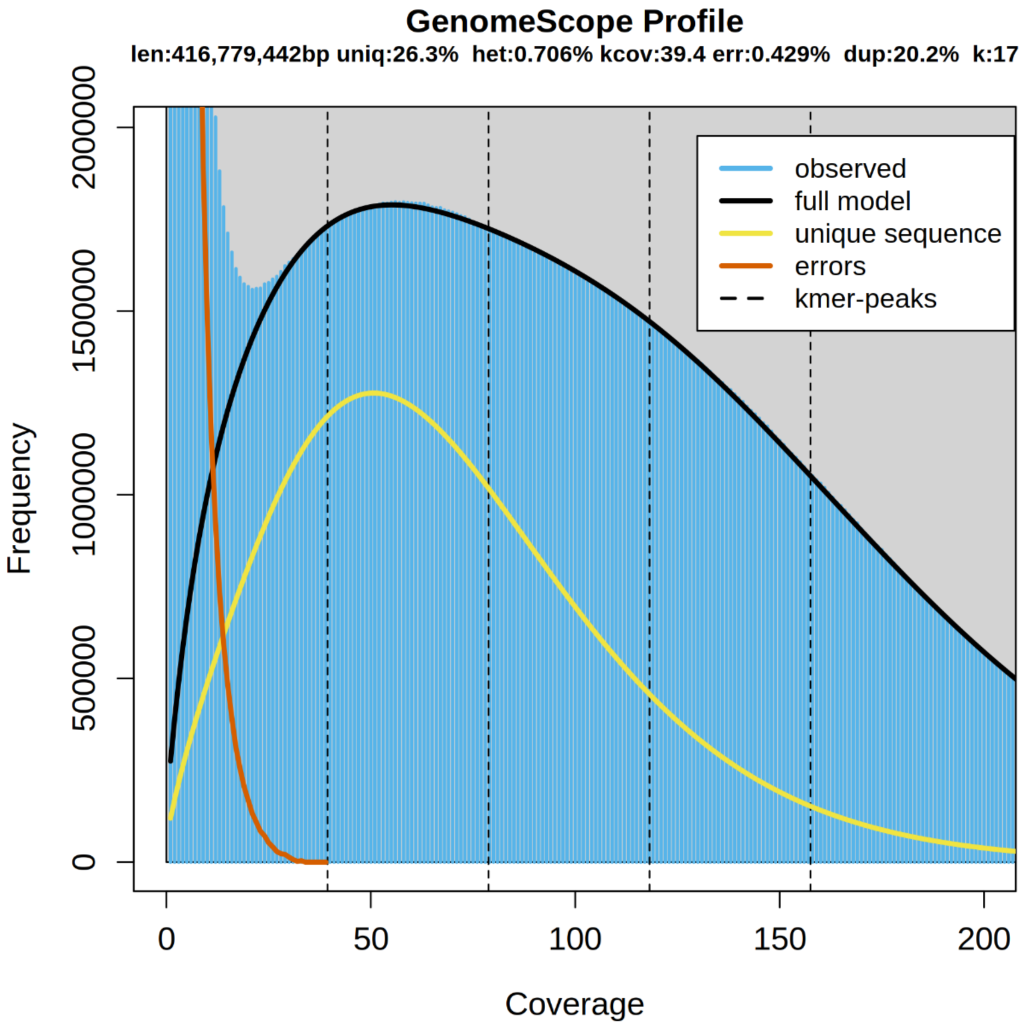

Fig. S1. Genome survey of *L. philippensis*

Sample1

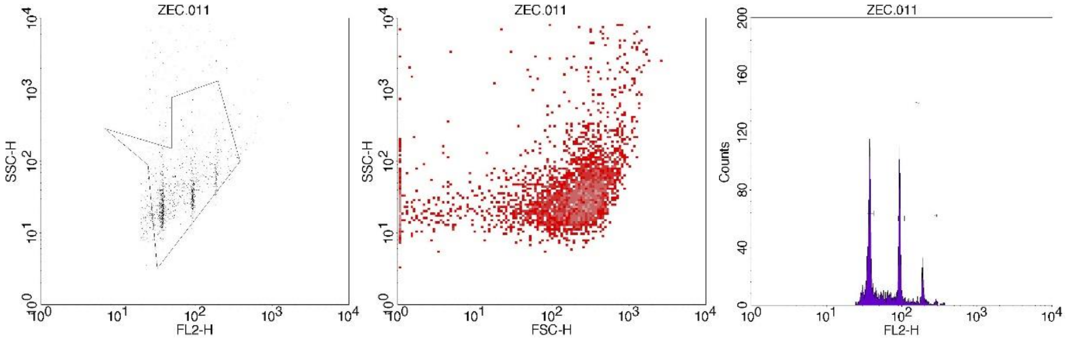

Sample2

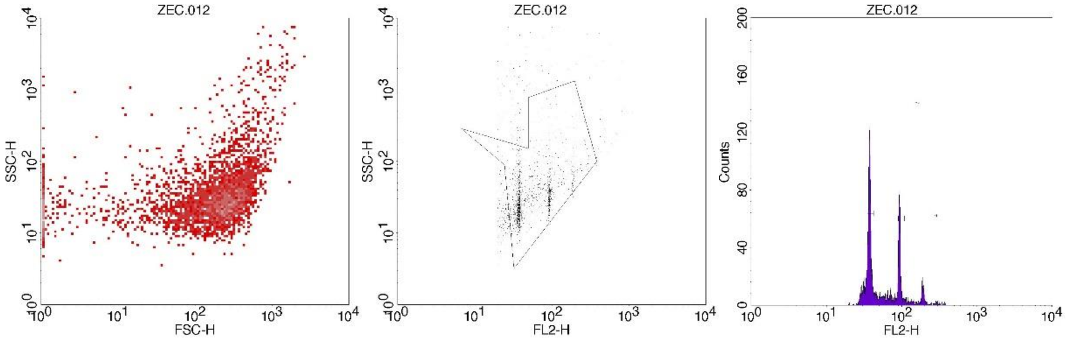

Sample3

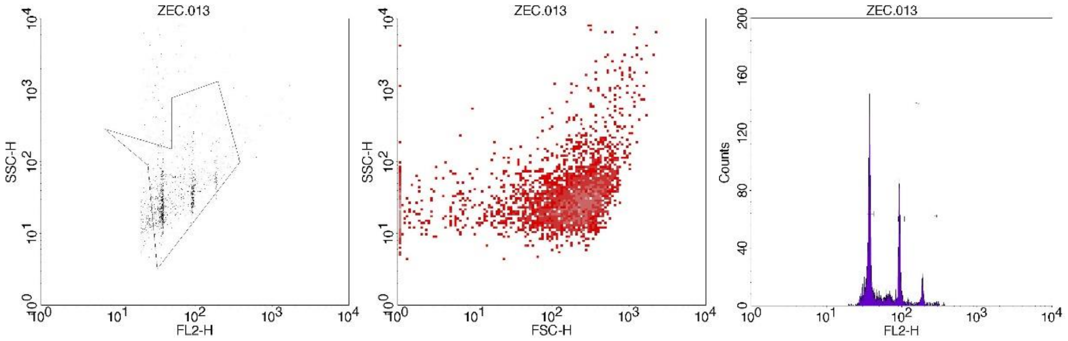

Fig. S2. Three repetitions of Flow cytometry analyses on *Lindenbergia philippensis*. The *Solanum lycopersicum* was chosen the as internal standard (1C =1 pg).

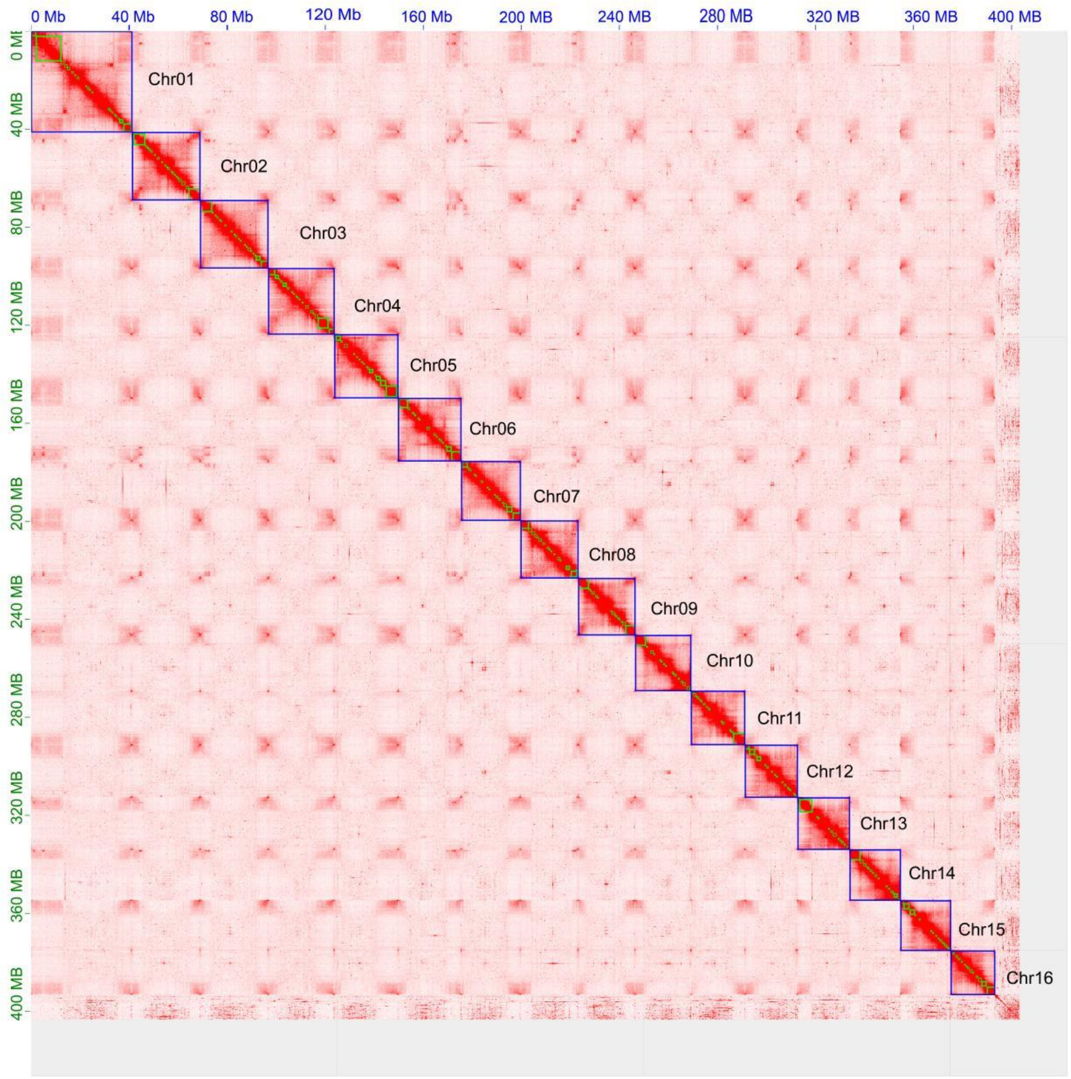

Fig. S3. Hi-C interaction heatmap and overview of the *L. philippensis* genome with a resolution of 500 kb. The green color of each square indicates contigs. The blue color of each square indicates the borders between scaffolds.

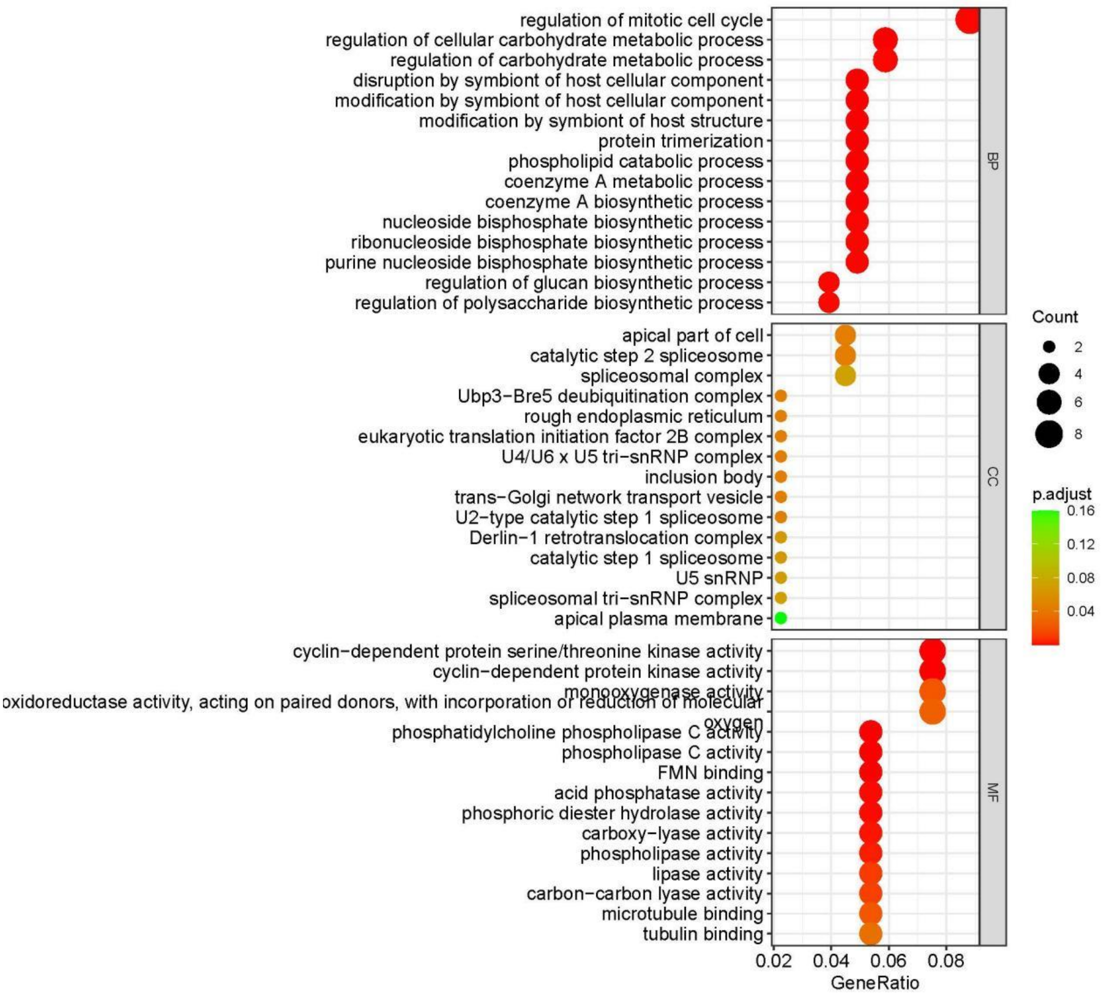

Fig. S4. GO enrichment analysis of species-specific genes for *L. philippensis*.

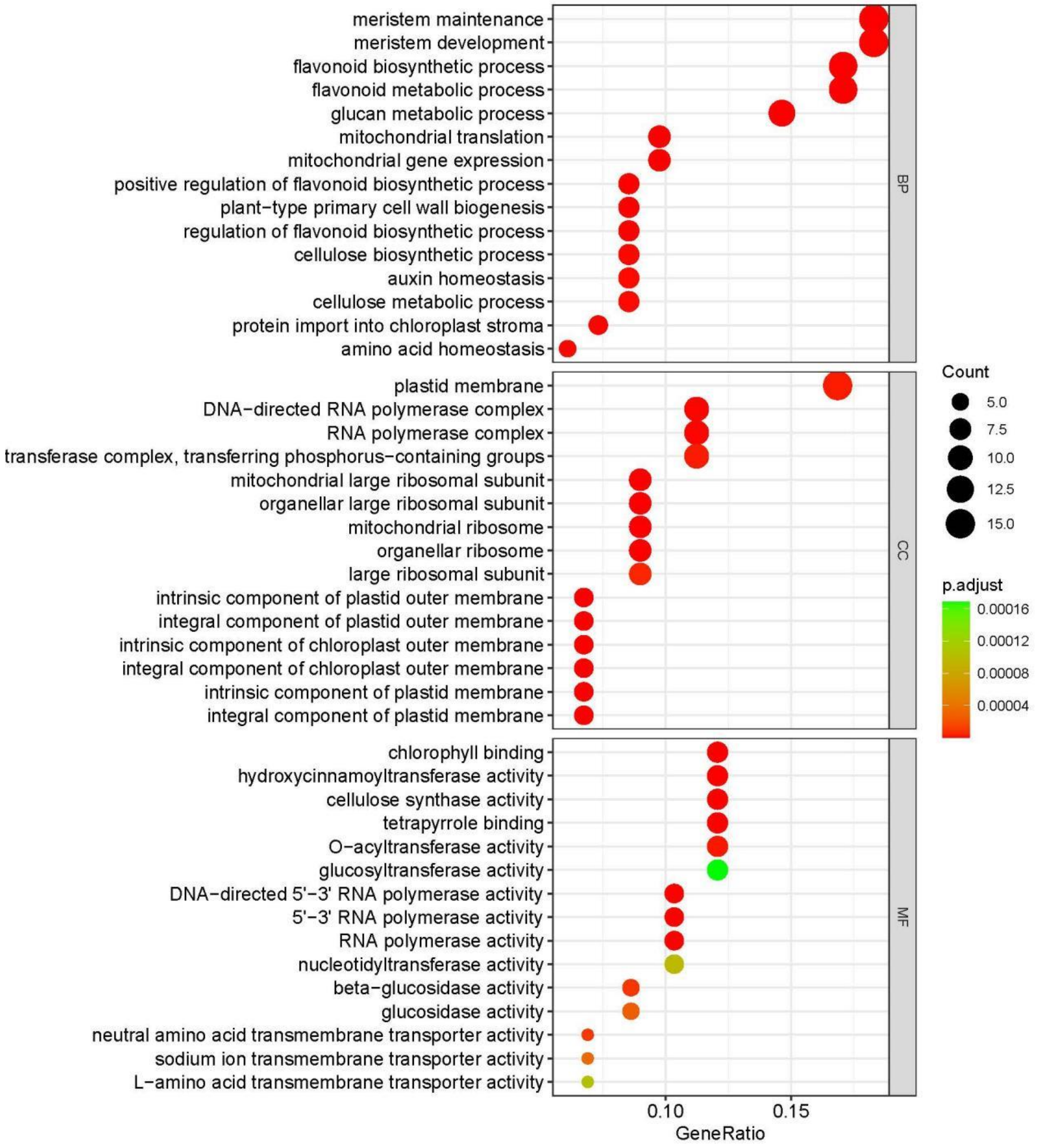

Fig. S5. GO enrichment analysis of rapidly expanded genes for *L. philippensis.*

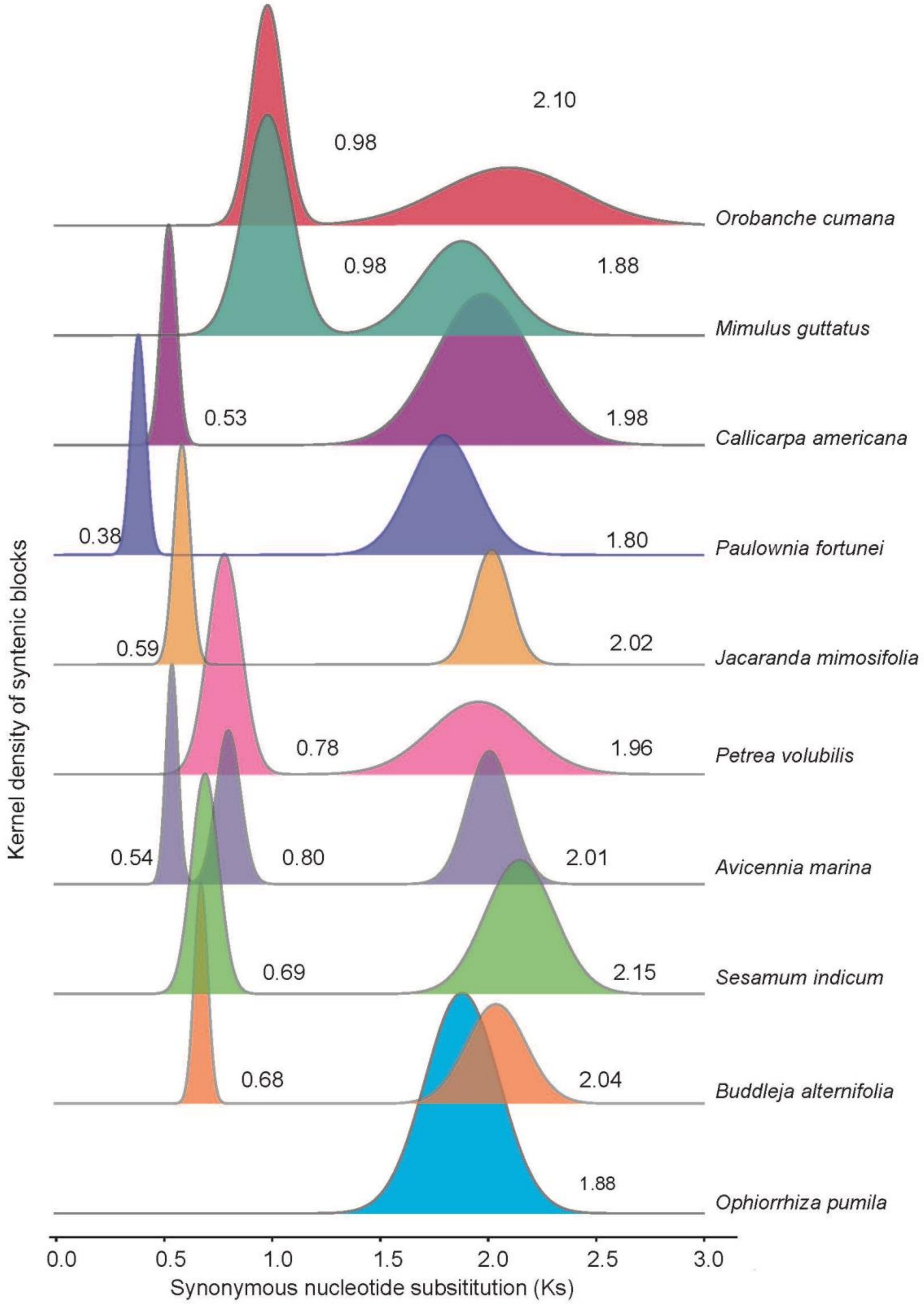

Fig. S6. Ks distributions of gene pairs in syntenic blocks among compared genomes.

A

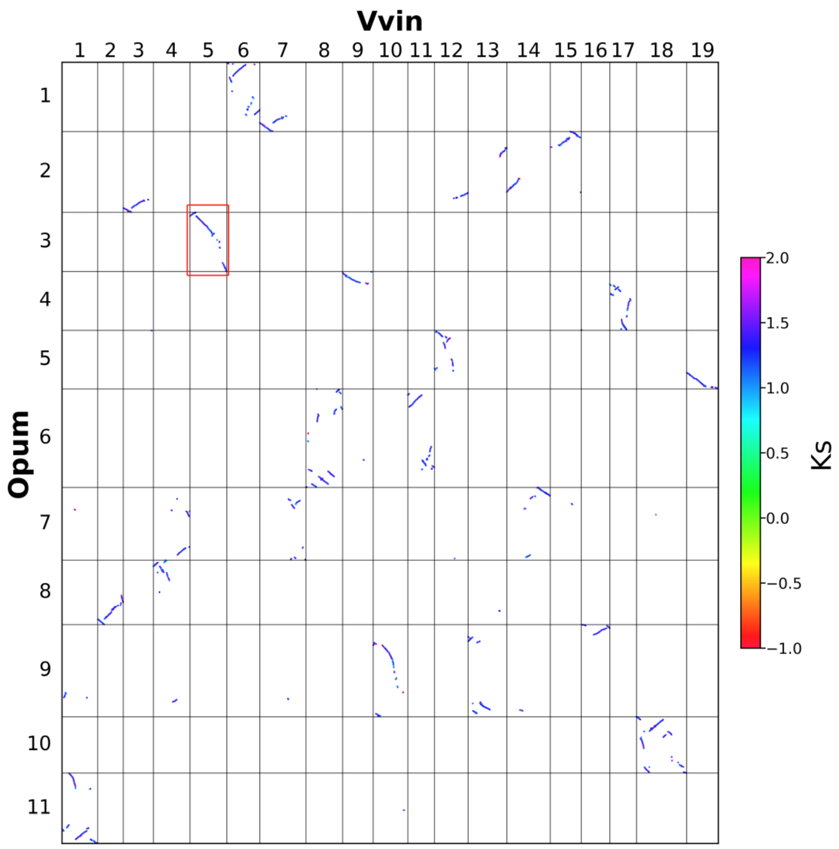

B

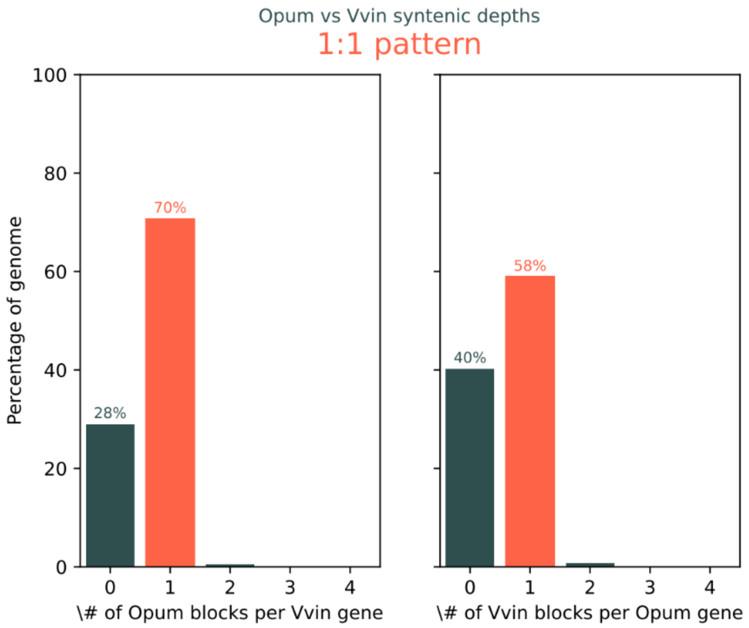

Fig. S7. Depth analysis of syntenic blocks between *O. pumila* (Opum) and *V. vinifera* (Vvin). (A) Syntenic dot plot between Opum and Vvin. The red box highlighted regions with a 1:1 orthologous gene ratio between Opum and Vvin. (B) The ratio of syntenic depth between Opum and Vvin. Syntenic blocks of Opum per Vvin gene (left) and syntenic blocks of Vvin per Opum gene (right) are shown suggesting a clear 1:1 pattern of orthologous gene ratio.

A

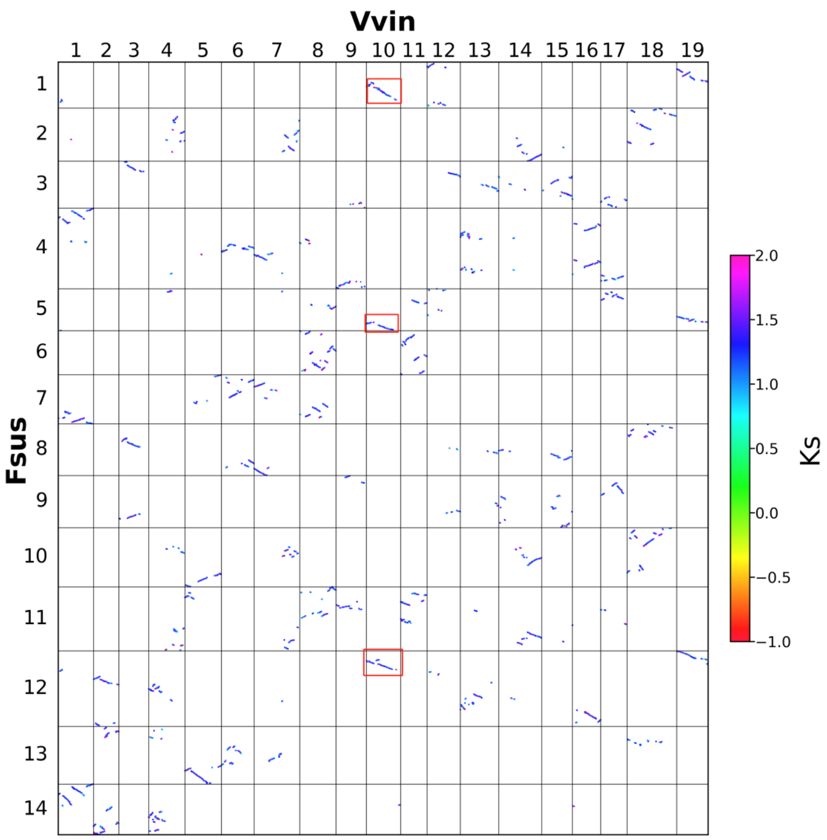

B

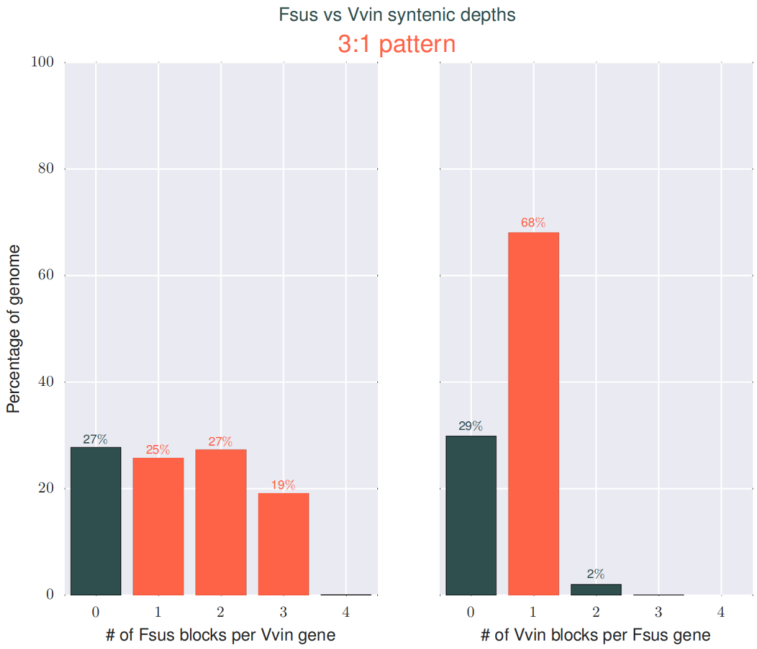

Fig. S8. Depth analysis of syntenic blocks between *F. suspensa* (Fsus) and *V. vinifera* (Vvin). (A) Syntenic dot plot between Fsus and Vvin. The red box highlighted regions with a 3:1 orthologous gene ratio between Fsus and Vvin. (B) The ratio of syntenic depth between Fsus and Vvin. Syntenic blocks of Fsus per Vvin gene (left) and syntenic blocks of Vvin per Fsus gene (right) are shown suggesting a clear 3:1 pattern of orthologous gene ratio.

A

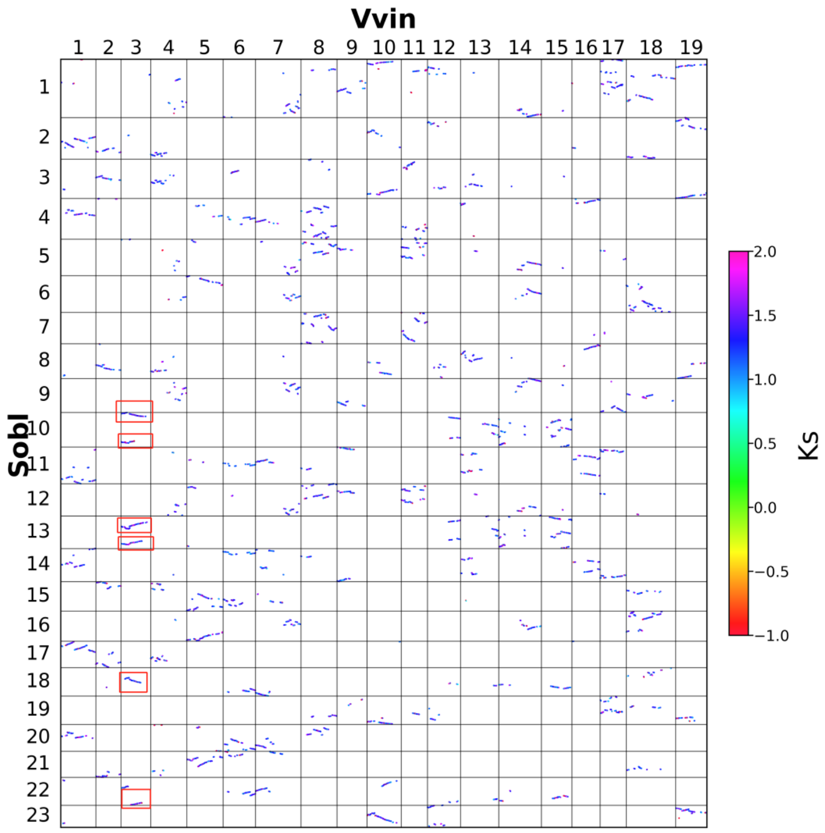

B

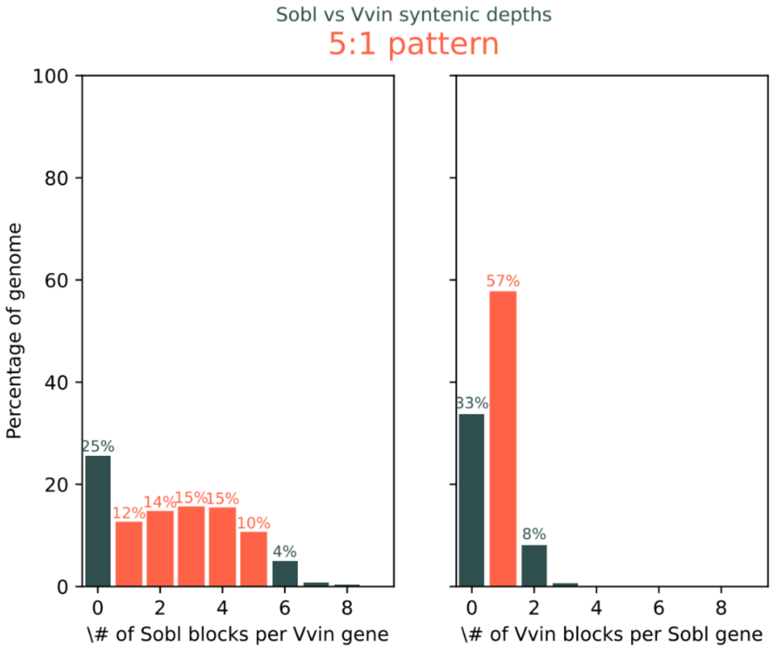

Fig. S9. Depth analysis of syntenic blocks between *S. oblate* (Sobl) and *V. vinifera* (Vvin). (A) Syntenic dot plot between Sobl and Vvin. The red box highlighted regions with a 6:1 orthologous gene ratio between Sobl and Vvin. (B) The ratio of syntenic depth between Sobl and Vvin. Syntenic blocks of Sobl per Vvin gene (left) and syntenic blocks of Vvin per Sobl gene (right) are shown suggesting a clear 5:1 pattern of orthologous gene ratio.

A

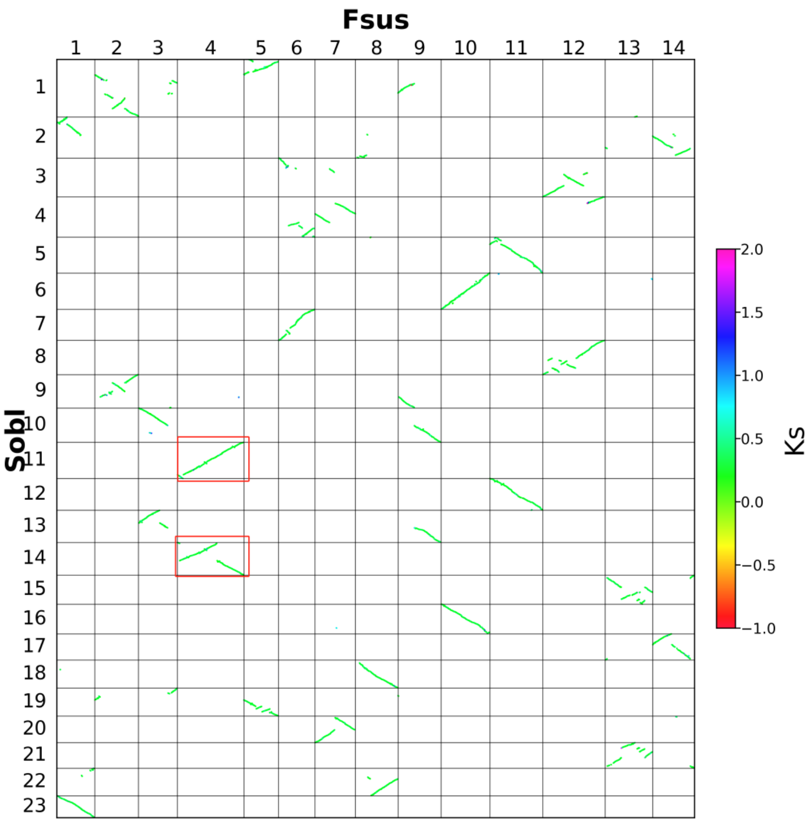

B

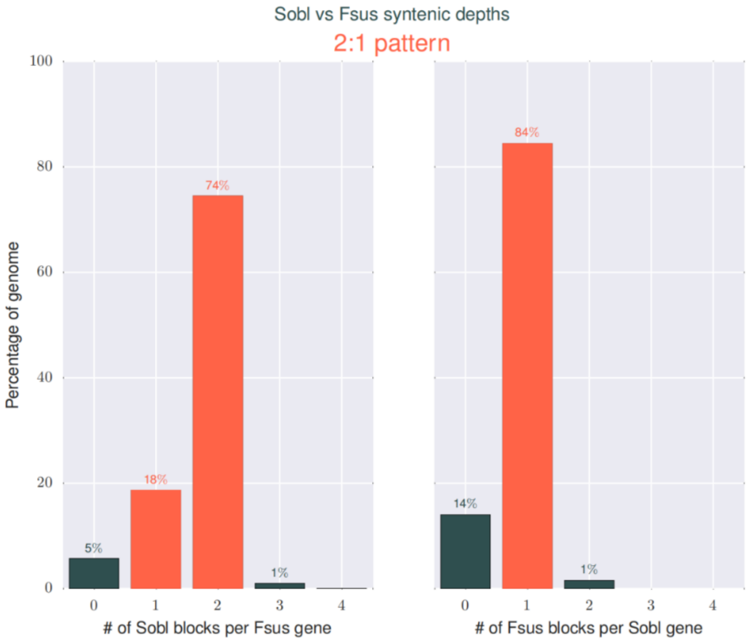

Fig. S10. Depth analysis of syntenic blocks between *S. oblate* (Sobl) and *F. suspense* (Fsus). (A) Syntenic dot plot between Sobl and Fsus. The red box highlighted regions with a 2:1 orthologous gene ratio between Sobl and Fsus. (B) The ratio of syntenic depth between Sobl and Fsus. Syntenic blocks of Sobl per Fsus gene (left) and syntenic blocks of Fsus per Sobl gene (right) are shown suggesting a clear 2:1 pattern of orthologous gene ratio.

A

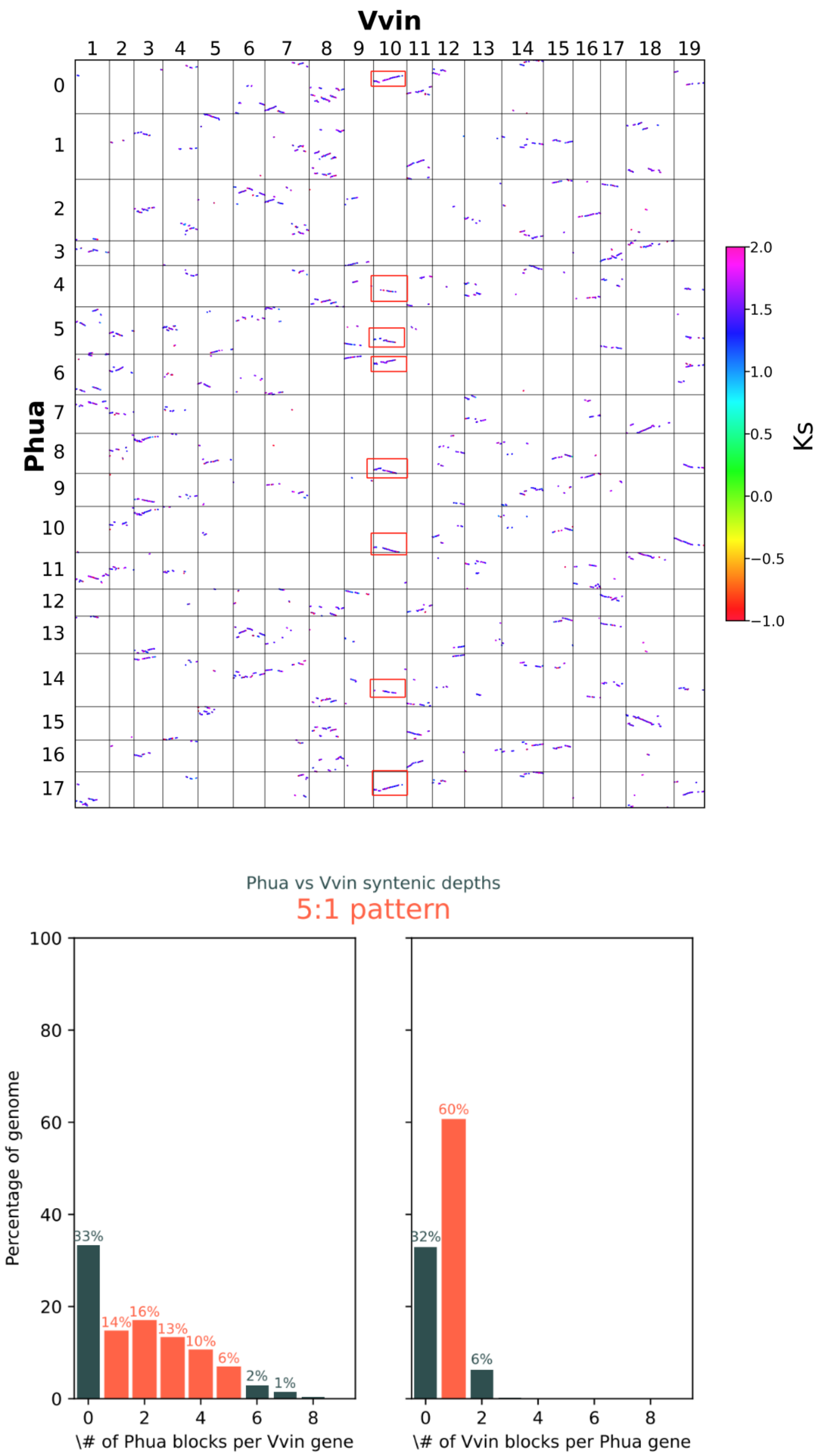

Fig. S11. Depth analysis of syntenic blocks between *P. huaijiensis* (Phua) and *V. vinifera* (Vvin). (A) Syntenic dot plot between Phua and Vvin. The red box highlighted regions with an 8:1 orthologous gene ratio between Phua and Vvin. (B) The ratio of syntenic depth between Phua and Vvin. Syntenic blocks of Phua per Vvin gene (left) and syntenic blocks of Vvin per Phua gene (right) are shown suggesting a clear 5:1 pattern of orthologous gene ratio.

A

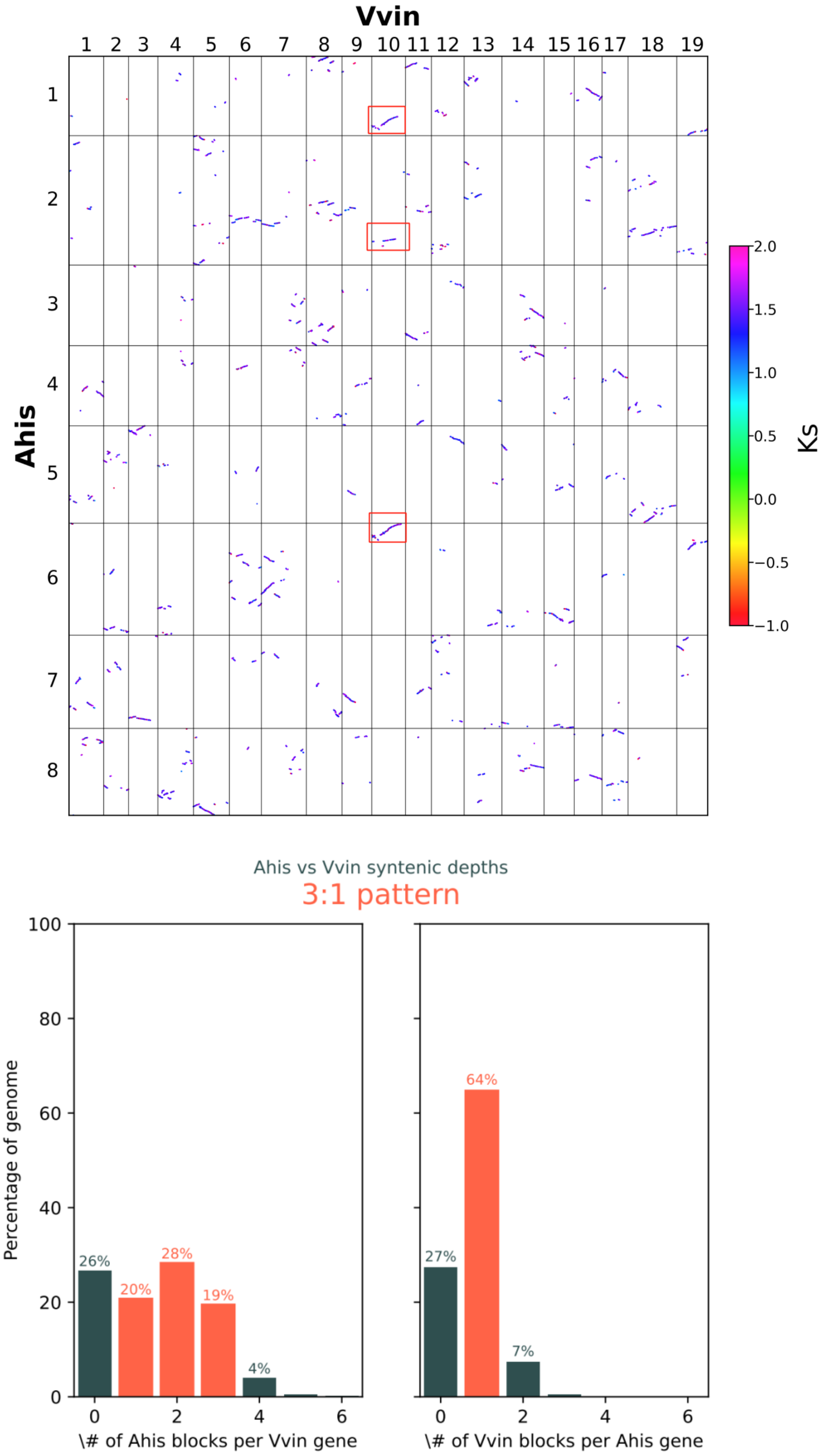

Fig. S12. Depth analysis of syntenic blocks between *A. hispanicum* (Ahis) and *V. vinifera* (Vvin). (A) Syntenic dot plot between Ahis and Vvin. The red box highlighted regions with a 3:1 orthologous gene ratio between Ahis and Vvin. (B) The atio of syntenic depth between Ahis and Vvin. Syntenic blocks of Ahis per Vvin gene (left) and syntenic blocks of Vvin per Ahis gene (right) are shown suggesting a clear 3:1 pattern of orthologous gene ratio.

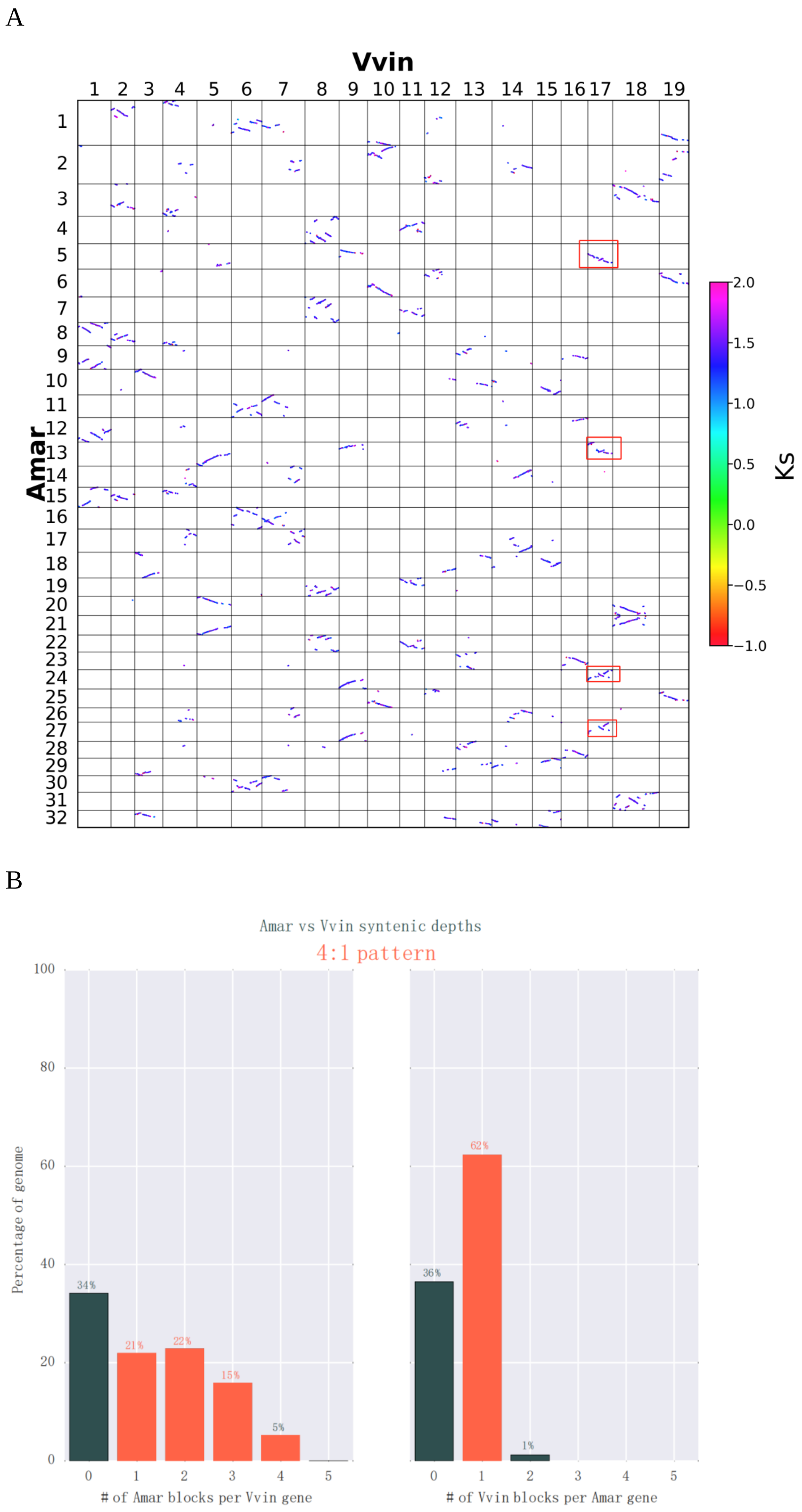

Fig. S13. Depth analysis of syntenic blocks between *A. marina* (Amar) and *V. vinifera* (Vvin). (A) Syntenic dot plot between Amar and Vvin. The red box highlighted regions with a 4:1 orthologous gene ratio between Amar and Vvin. (B) The ratio of syntenic depth between Amar and Vvin. Syntenic blocks of Amar per Vvin gene (left) and syntenic blocks of Vvin per Amar gene (right) are shown suggesting a clear 4:1 pattern of orthologous gene ratio.

A

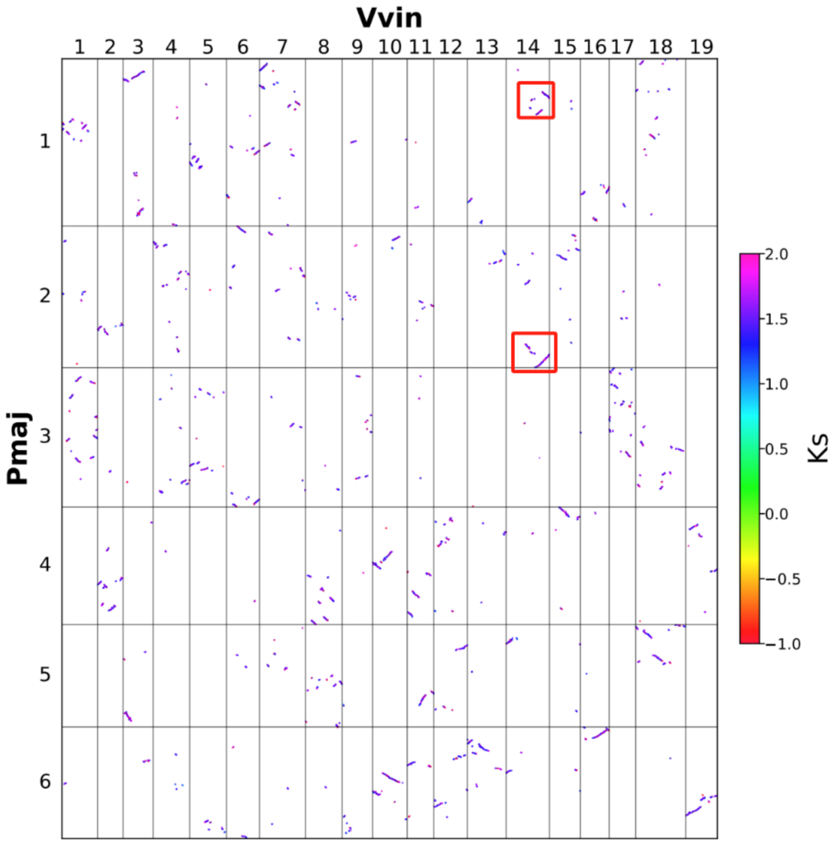

B

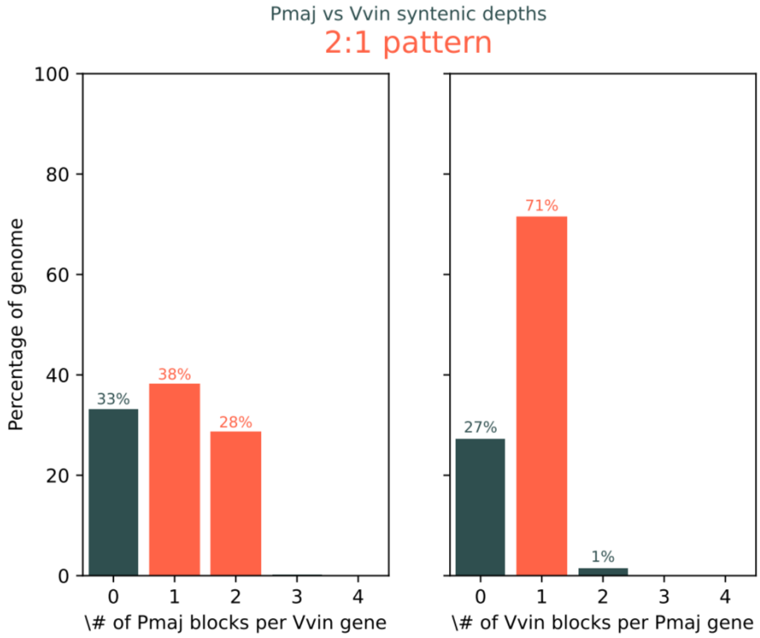

Fig. S14. Depth analysis of syntenic blocks between *P. major* (Pmaj) and *V. vinifera* (Vvin). (A) Syntenic dot plot between Pmaj and Vvin. The red box highlighted regions with a 2:1 orthologous gene ratio between Pmaj and Vvin. (B) The ratio of syntenic depth between Pmaj and Vvin. Syntenic blocks of Pmaj per Vvin gene (left) and syntenic blocks of Vvin per Pmaj gene (right) are shown suggesting a clear 2:1 pattern of orthologous gene ratio.

A

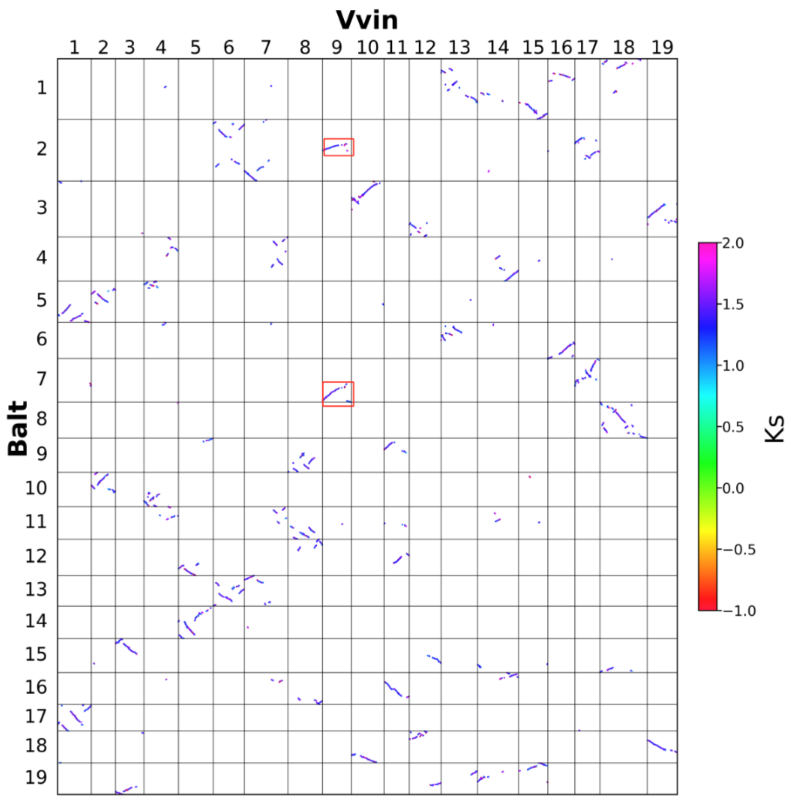

B

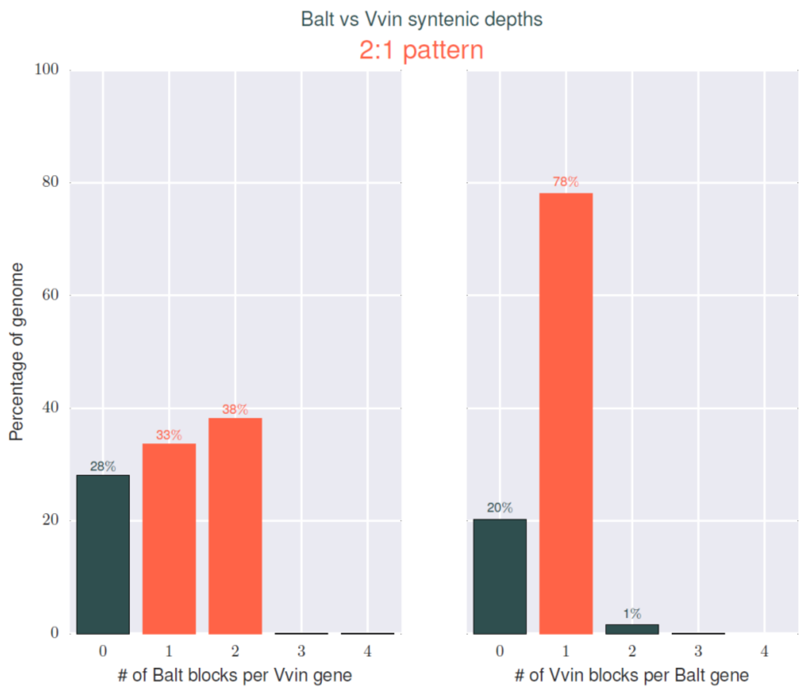

Fig. S15. Depth analysis of syntenic blocks between *B. alternifolia* (Balt) and *V. vinifera* (Vvin). (A) Syntenic dot plot between Balt and Vvin. The red box highlighted regions with a 2:1 orthologous gene ratio between Balt and Vvin. (B) The ratio of syntenic depth between Balt and Vvin. Syntenic blocks of Balt per Vvin gene (left) and syntenic blocks of Vvin per Balt gene (right) are shown suggesting a clear 2:1 pattern of orthologous gene ratio.

A

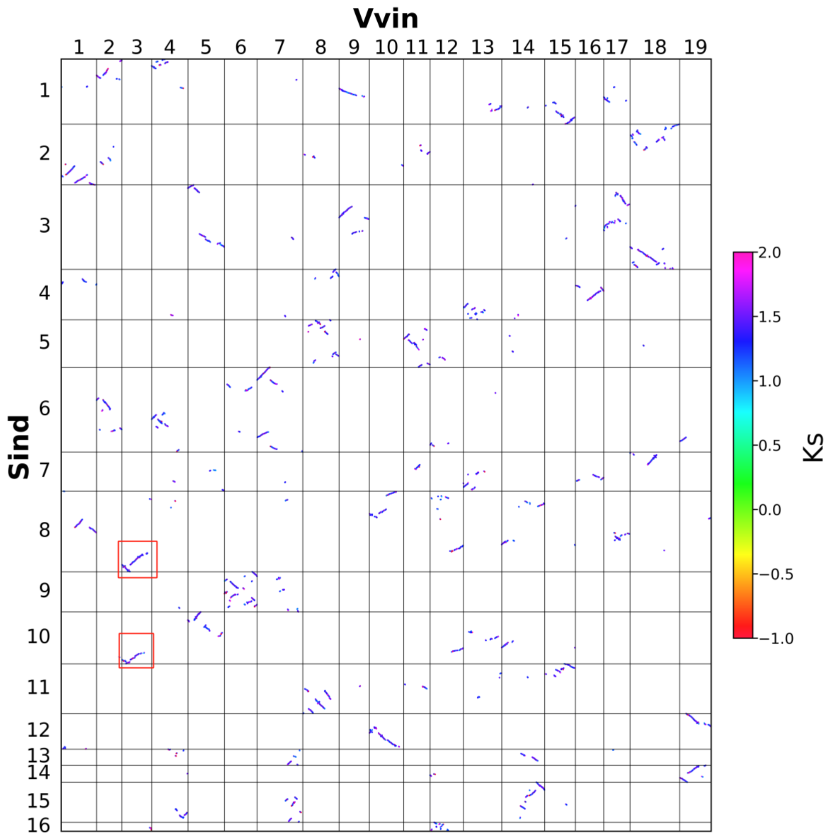

B

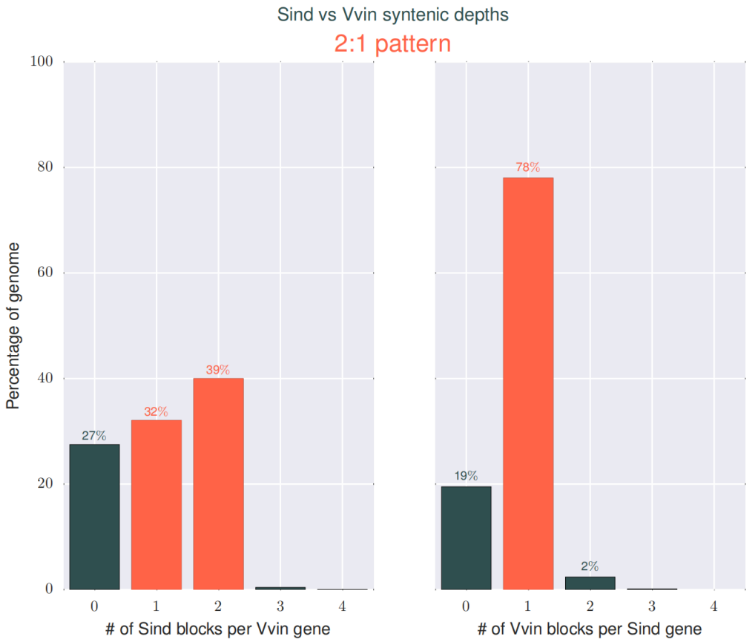

Fig. S16. Depth analysis of syntenic blocks between *S. indicum* (Sind) and *V. vinifera* (Vvin). (A) Syntenic dot plot between Sind and Vvin. The red box highlighted regions with a 2:1 orthologous gene ratio between Sind and Vvin. (B) The ratio of syntenic depth between Sind and Vvin. Syntenic blocks of Sind per Vvin gene (left) and syntenic blocks of Vvin per Sind gene (right) are shown suggesting a clear 2:1 pattern of orthologous gene ratio.

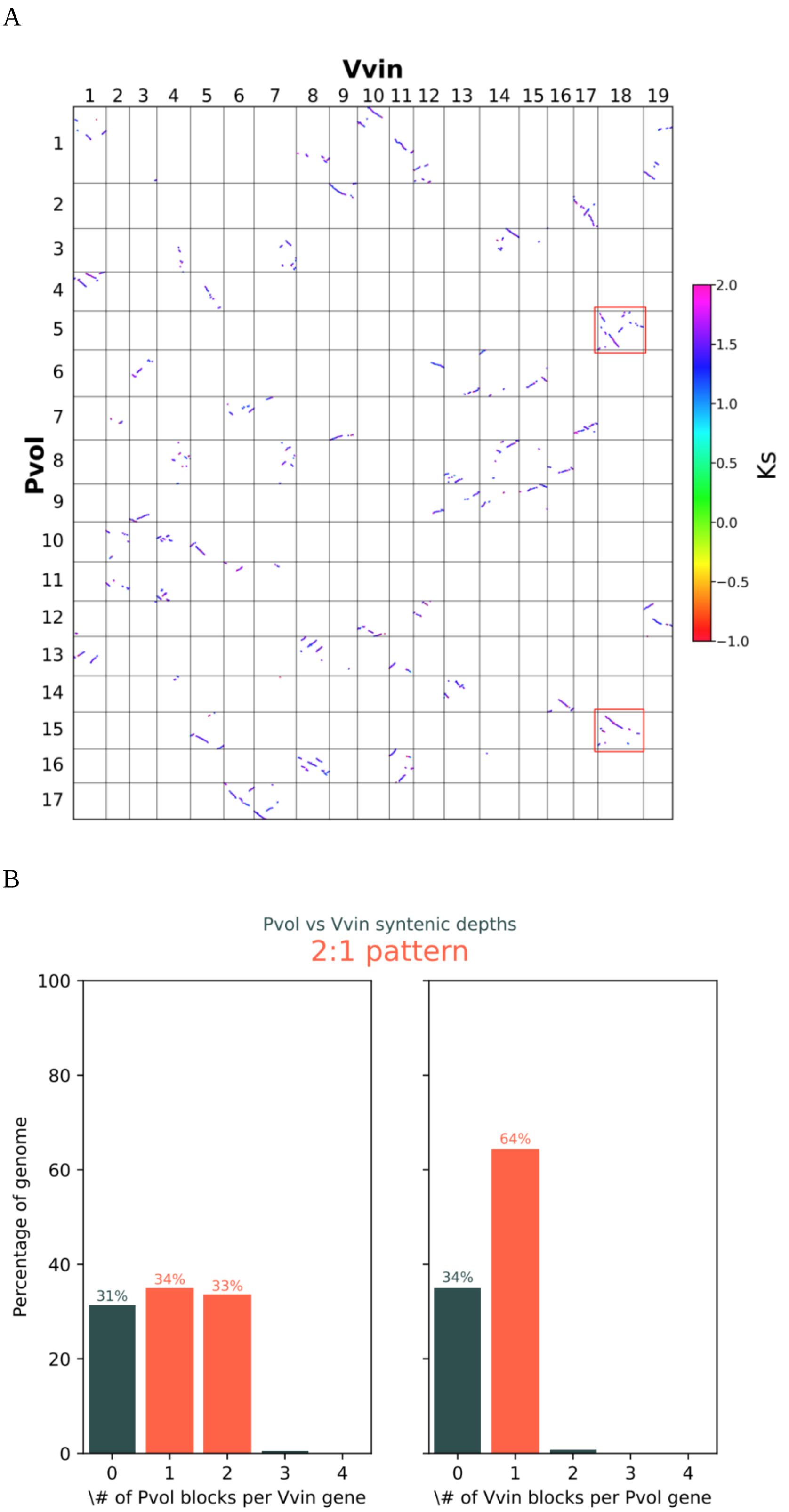

Fig. S17. Depth analysis of syntenic blocks between *P. volubilis* (Pvol) and *V. vinifera* (Vvin). (A) Syntenic dot plot between Pvol and Vvin. The red box highlighted regions with a 2:1 orthologous gene ratio between Pvol and Vvin. (B) The ratio of syntenic depth between Pvol and Vvin. Syntenic blocks of Pvol per Vvin gene (left) and syntenic blocks of Vvin per Pvol gene (right) are shown suggesting a clear 2:1 pattern of orthologous gene ratio.

A

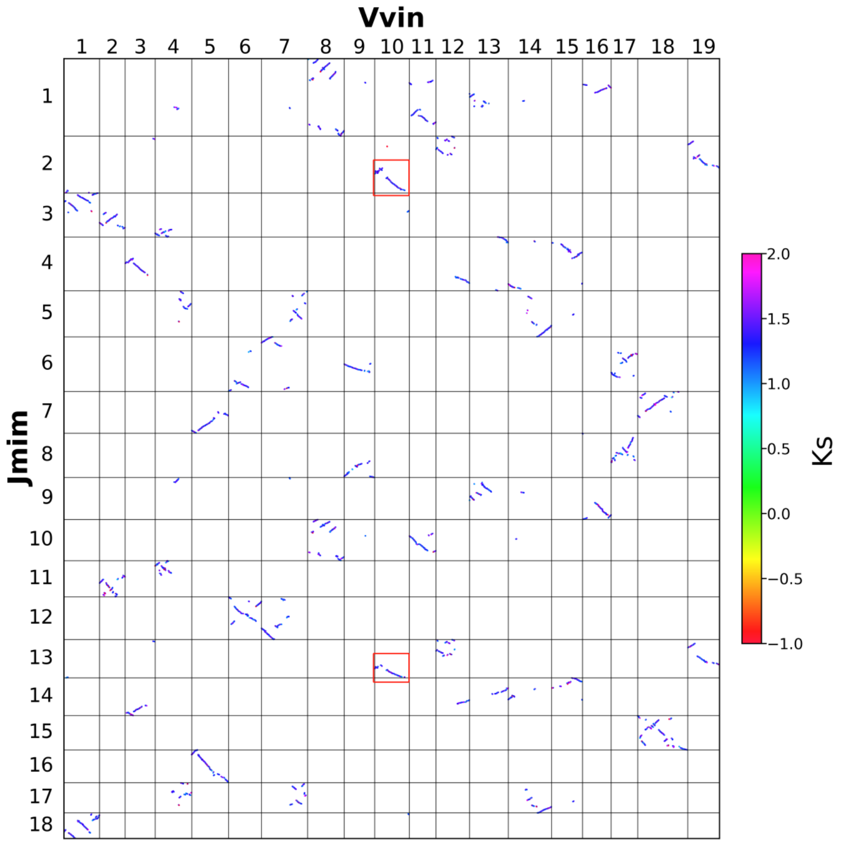

B

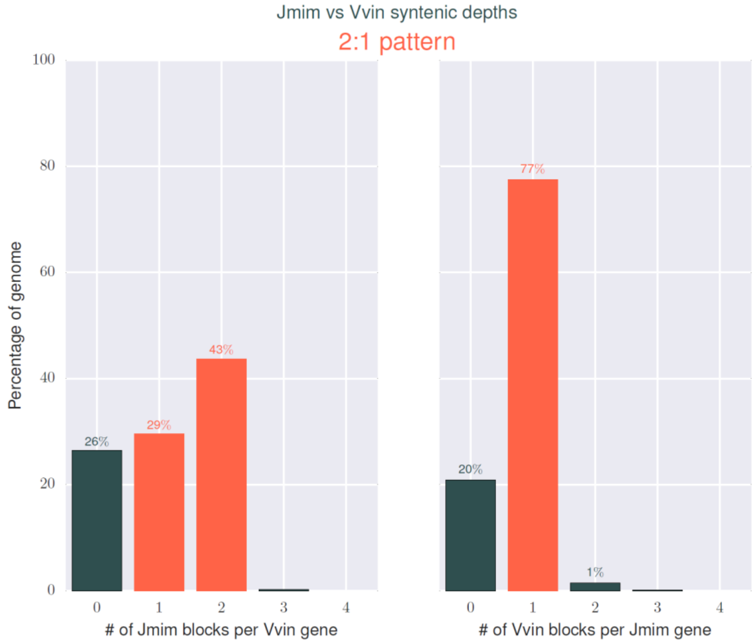

Fig. S18. Depth analysis of syntenic blocks between *J. mimosifolia* (Jmim) and *V. vinifera* (Vvin). (A) Syntenic dot plot between Jmim and Vvin. The red box highlighted regions with a 2:1 orthologous gene ratio between Jmim and Vvin. (B) The ratio of syntenic depth between Jmim and Vvin. Syntenic blocks of Jmim per Vvin gene (left) and syntenic blocks of Vvin per Jmim gene (right) are shown suggesting a clear 2:1 pattern of orthologous gene ratio.

A

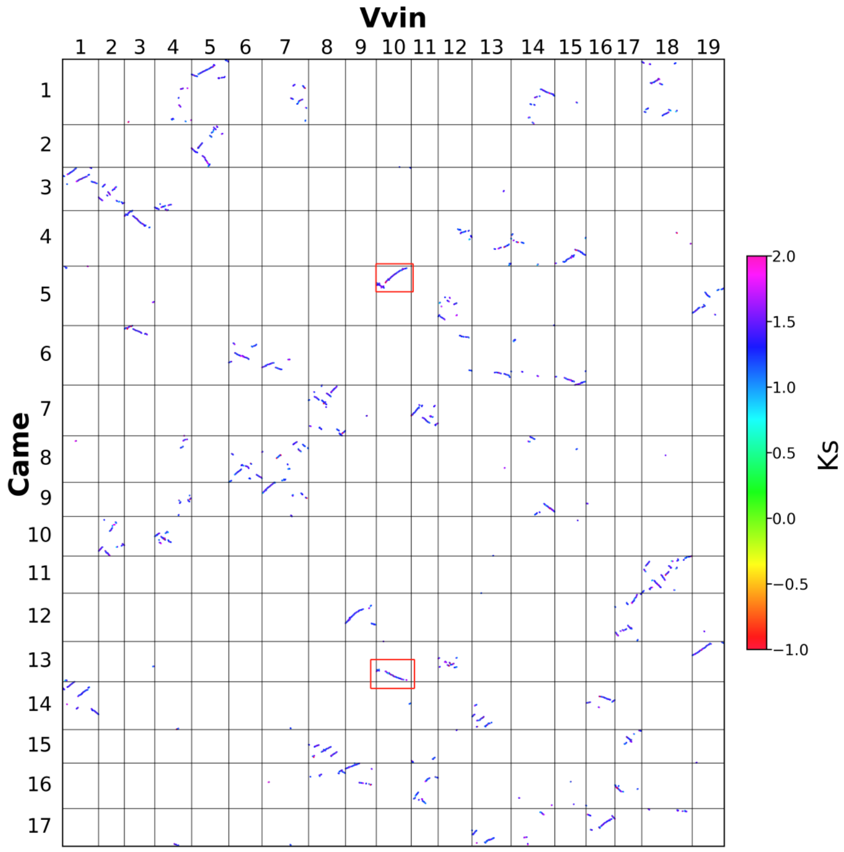

B

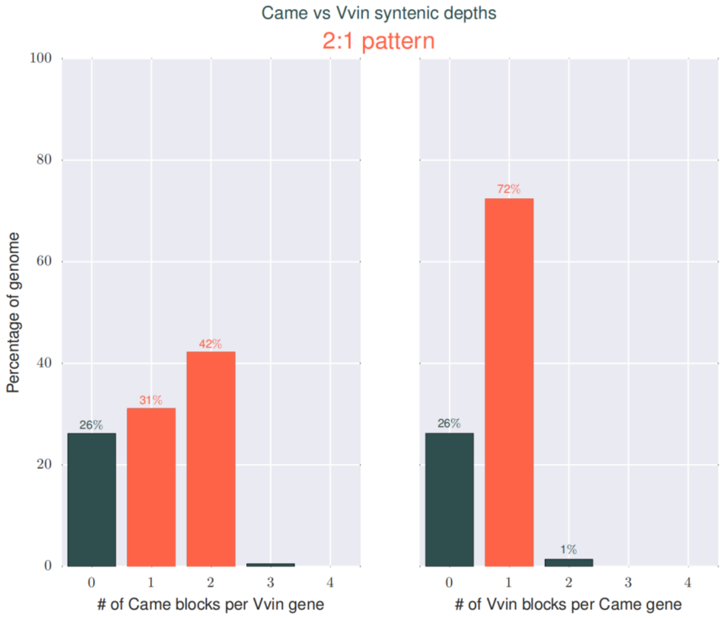

Fig. S19. Depth analysis of syntenic blocks between *C. americana* (Came) and *V. vinifera* (Vvin). (A) Syntenic dot plot between Came and Vvin. The red box highlighted regions with a 2:1 orthologous gene ratio between Came and Vvin. (B) The ratio of syntenic depth between Came and Vvin. Syntenic blocks of Came per Vvin gene (left) and syntenic blocks of Vvin per Came gene (right) are shown suggesting a clear 2:1 pattern of orthologous gene ratio.

A

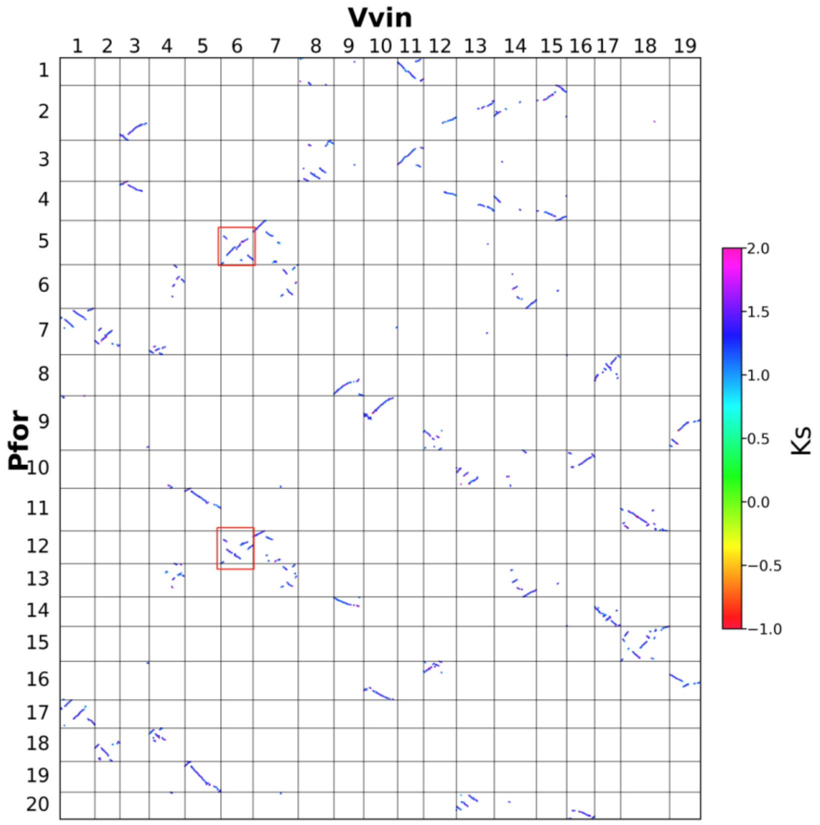

B

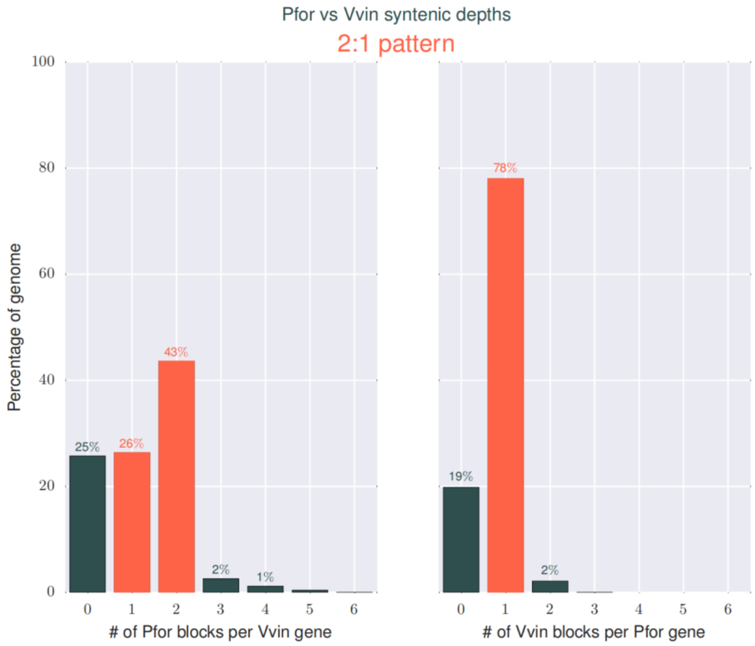

Fig. S20. Depth analysis of syntenic blocks between *P. fortunei* (Pfor) and *V. vinifera* (Vvin). (A) Syntenic dot plot between Pfor and Vvin. The red box highlighted regions with a 2:1 orthologous gene ratio between Pfor and Vvin. (B) The ratio of syntenic depth between Pfor and Vvin. Syntenic blocks of Pfor per Vvin gene (left) and syntenic blocks of Vvin per Pfor gene (right) are shown suggesting a clear 2:1 pattern of orthologous gene ratio.

A

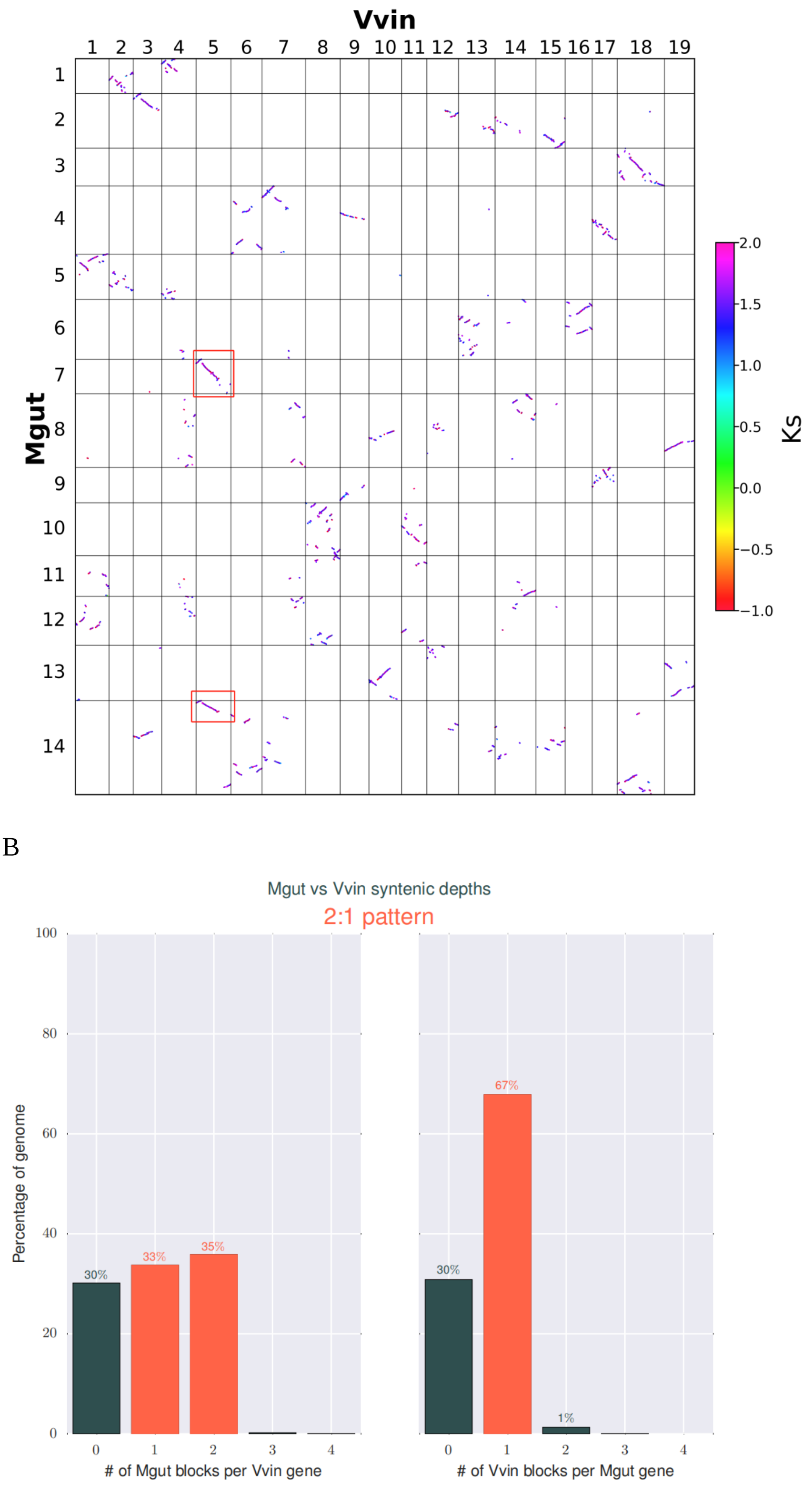

B

Fig. S21. Depth analysis of syntenic blocks between *M. guttatus* (Mgut) and *V. vinifera* (Vvin). (A) Syntenic dot plot between Mgut and Vvin. The red box highlighted regions with a 2:1 orthologous gene ratio between Mgut and Vvin. (B) The atio of syntenic depth between Mgut and Vvin. Syntenic blocks of Mgut per Vvin gene (left) and syntenic blocks of Vvin per Mgut gene (right) are shown suggesting a clear 2:1 pattern of orthologous gene ratio.

A

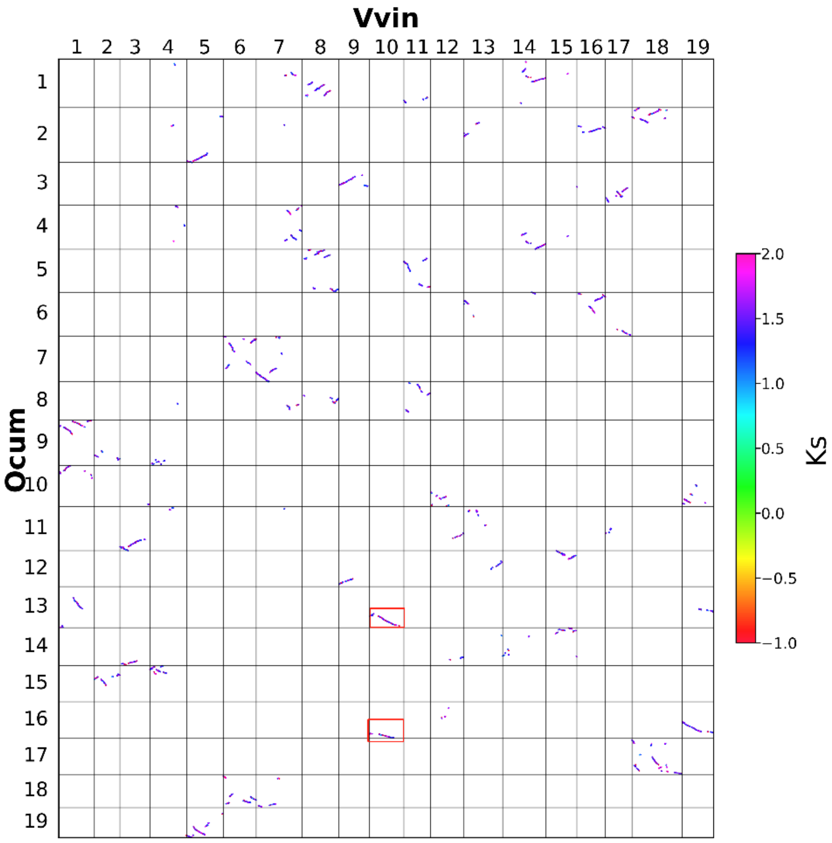

B

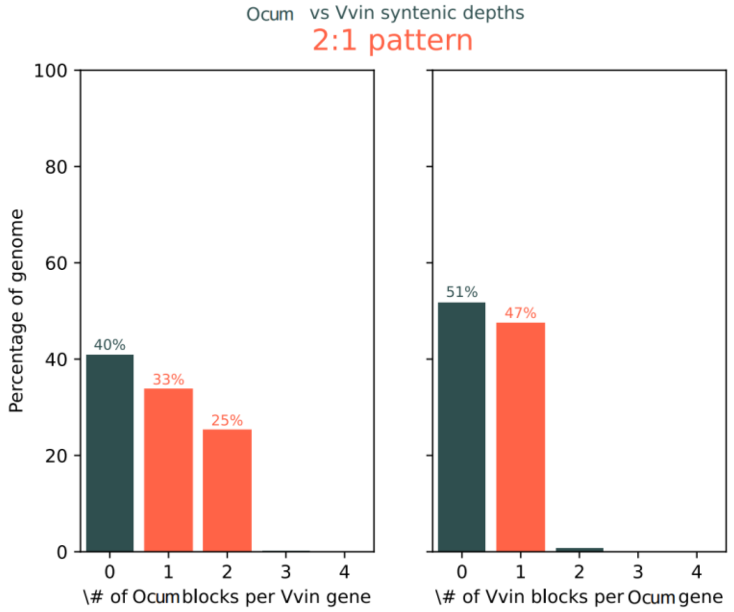

Fig. S22. Depth analysis of syntenic blocks between *O. cumana* (Ocum) and *V. vinifera* (Vvin). (A) Syntenic dot plot between Ocum and Vvin. The red box highlighted regions with a 2:1 orthologous gene ratio between Ocum and Vvin. (B) The ratio of syntenic depth between Ocum and Vvin. Syntenic blocks of Ocum per Vvin gene (left) and syntenic blocks of Vvin per Ocum gene (right) are shown suggesting a clear 2:1 pattern of orthologous gene ratio.

A

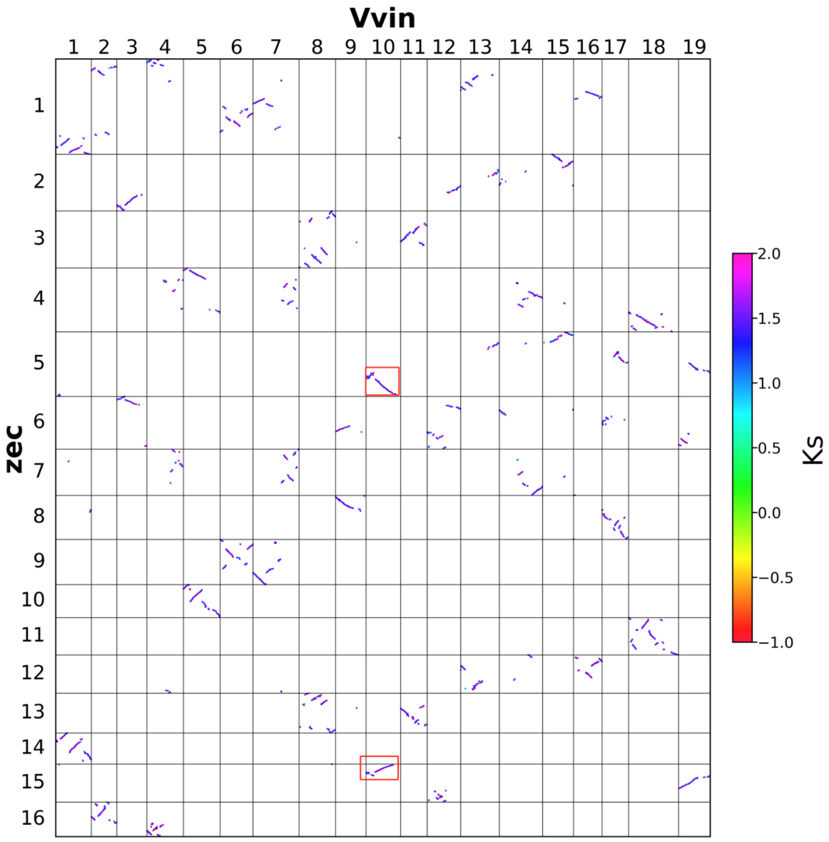

B

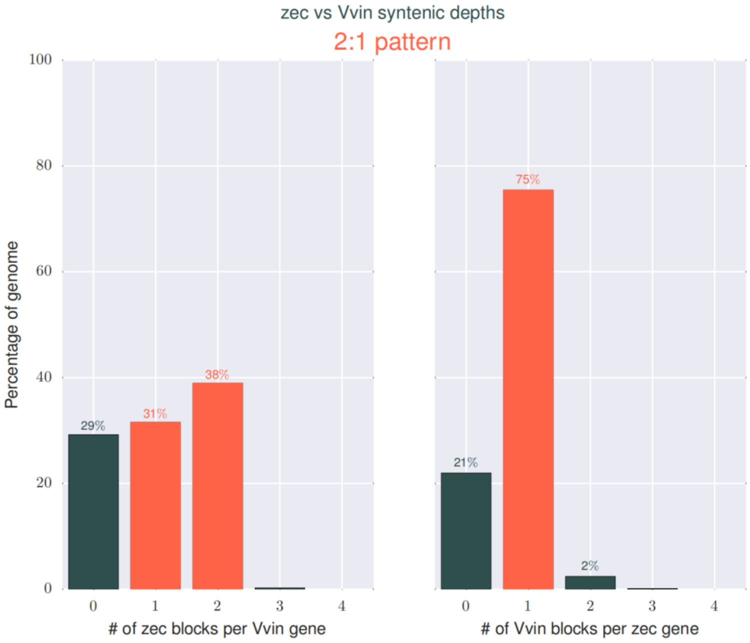

Fig. S23. Depth analysis of syntenic blocks between *L. philippensis* (zec) and *V. vinifera* (Vvin). (A) Syntenic dot plot between zec and Vvin. The red box highlighted regions with a 2:1 orthologous gene ratio between zec and Vvin. (B) The ratio of syntenic depth between zec and Vvin. Syntenic blocks of zec per Vvin gene (left) and syntenic blocks of Vvin per zec gene (right) are shown suggesting a clear 2:1 pattern of orthologous gene ratio.

A

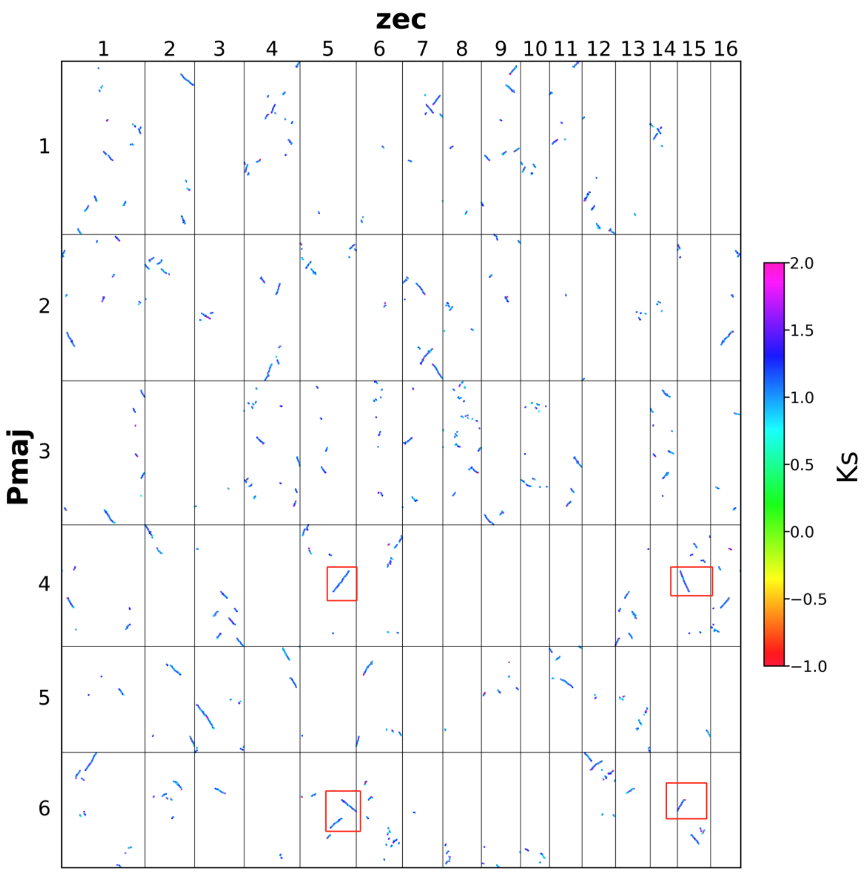

B

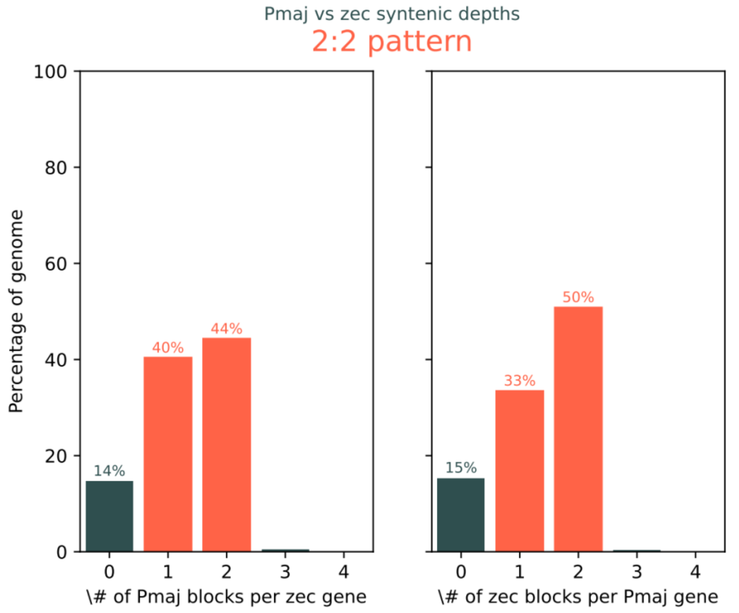

Fig. S24. Depth analysis of syntenic blocks between *P. major* (Pmaj) and *L. philippensis* (zec). (A) Syntenic dot plot between Pmaj and zec. The red box highlighted regions with a 2:2 orthologous gene ratio between Pmaj and zec. (B) The ratio of syntenic depth between Pmaj and zec. Syntenic blocks of Pmaj per zec gene (left) and syntenic blocks of zec per Pmaj gene (right) are shown suggesting a clear 2:2 pattern of orthologous gene ratio.

A

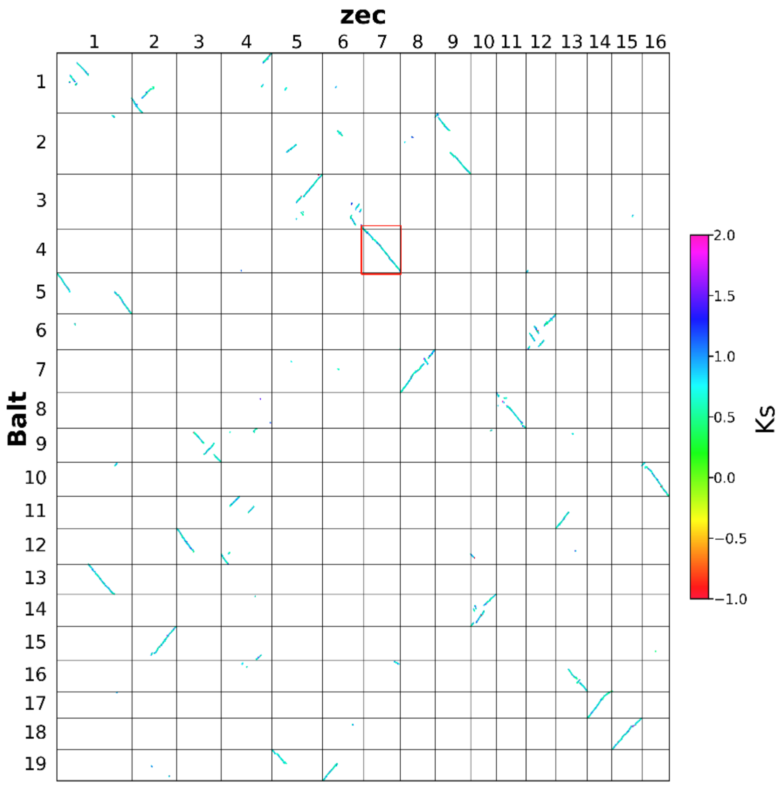

B

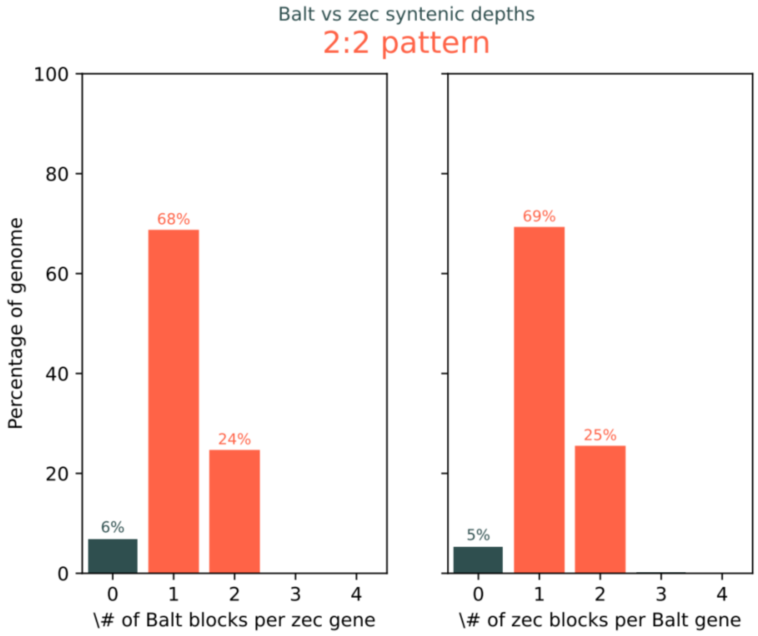

Fig. S25. Depth analysis of syntenic blocks between *B. alternifolia* (Balt) and *L. philippensis* (zec). (A) Syntenic dot plot between Balt and zec. The ed box highlighted regions with a 1:1 orthologous gene ratio between Balt and zec. (B) The ratio of syntenic depth between Balt and zec. Syntenic blocks of Balt per zec gene (left) and syntenic blocks of zec per Balt gene (right) are shown suggesting a clear 1:1 pattern of orthologous gene ratio.

A

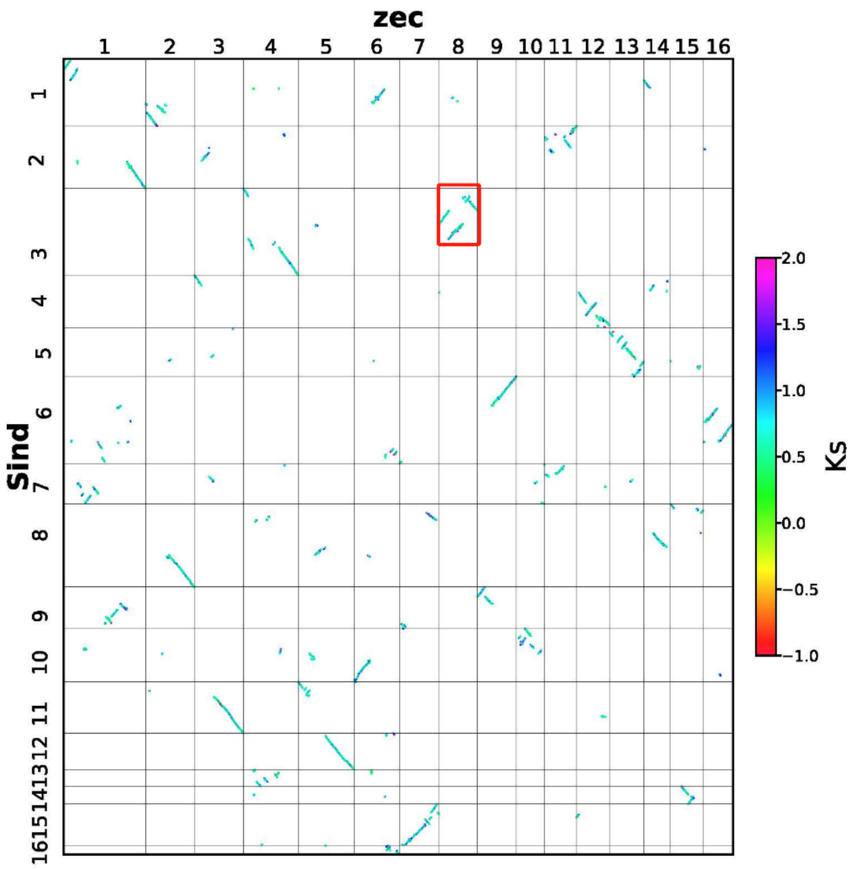

B

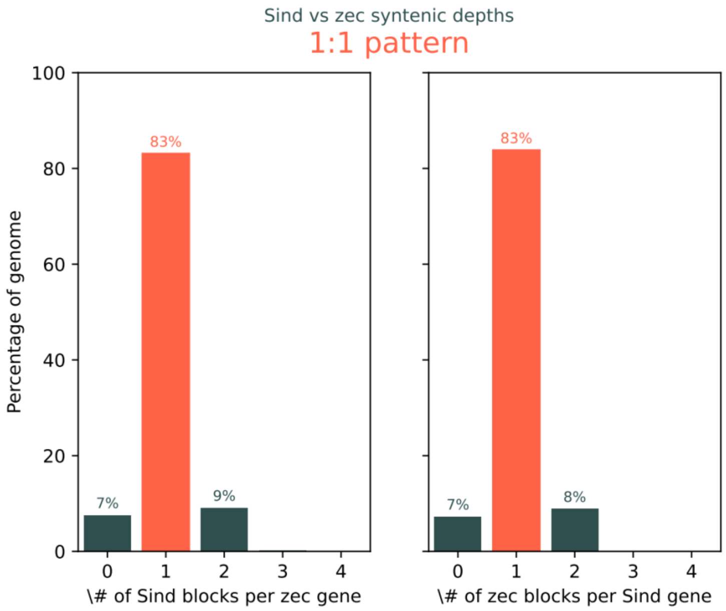

Fig. S26. Depth analysis of syntenic blocks between *S. indicum* (Sind) and *L. philippensis* (zec). (A) Syntenic dot plot between Sind and zec. The red box highlighted regions with a 1:1 orthologous gene ratio between Sind and zec. (B) The ratio of syntenic depth between Sind and zec. Syntenic blocks of Sind per zec gene (left) and syntenic blocks of zec per Sind gene (right) are shown suggesting a clear 1:1 pattern of orthologous gene ratio.

A

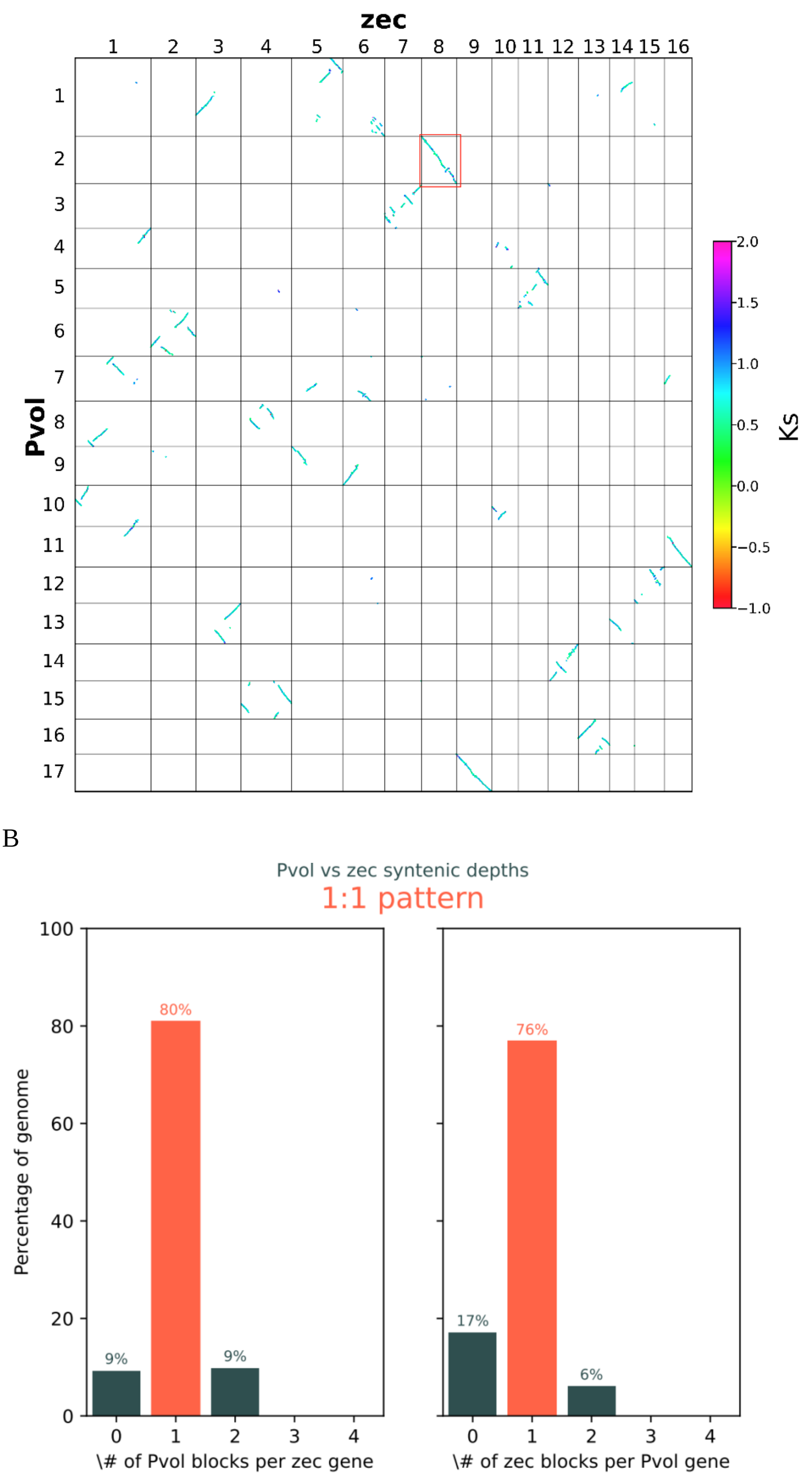

Fig. S27. Depth analysis of syntenic blocks between *P. volubilis* (Pvol) and *L. philippensis* (zec). (A) Syntenic dot plot between Pvol and zec. The red box highlighted regions with a 1:1 orthologous gene ratio between Pvol and zec. (B) The ratio of syntenic depth between Pvol and zec. Syntenic blocks of Pvol per zec gene (left) and syntenic blocks of zec per Pvol gene (right) are shown suggesting a clear 1:1 pattern of orthologous gene ratio.

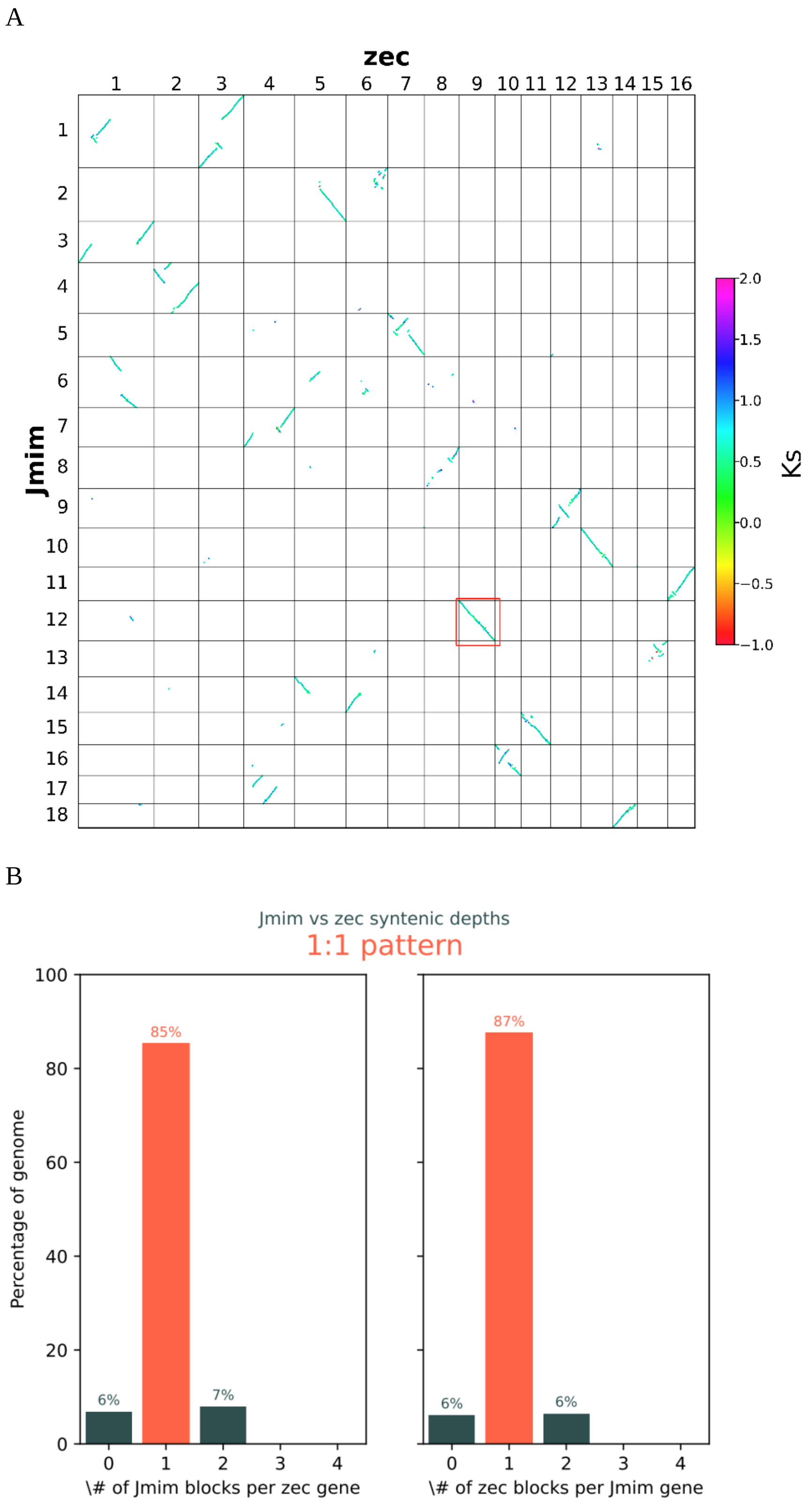

Fig. S28. Depth analysis of syntenic blocks between *J. mimosifolia* (Jmim) and *L. philippensis* (zec). (A) Syntenic dot plot between Jmim and zec. Red box highlighted regions with 1:1 orthologous gene ratio between Jmim and zec. (B) Ratio of syntenic depth between Jmim and zec. Syntenic blocks of Jmim per zec gene (left) and syntenic blocks of zec per Jmim gene (right) are shown suggesting a clear 1:1 pattern of orthologous gene ratio.

A

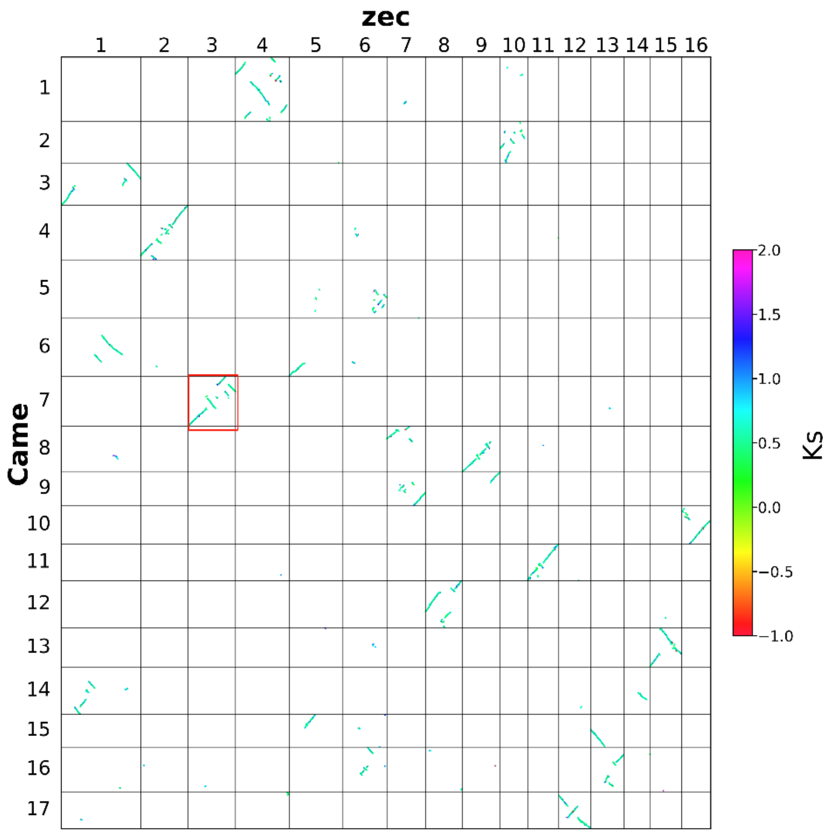

B

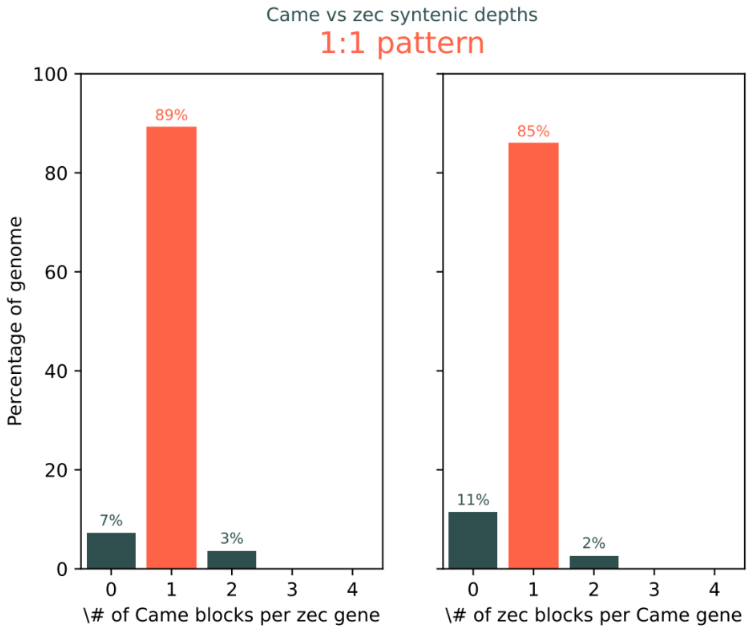

Fig. S29. Depth analysis of syntenic blocks between *C. americana* (Came) and *L. philippensis* (zec). (A) Syntenic dot plot between Came and zec. The red box highlighted regions with a 1:1 orthologous gene ratio between Came and zec. (B) The ratio of syntenic depth between Came and zec. Syntenic blocks of Came per zec gene (left) and syntenic blocks of zec per Came gene (right) are shown suggesting a clear 1:1 pattern of orthologous gene ratio.

A

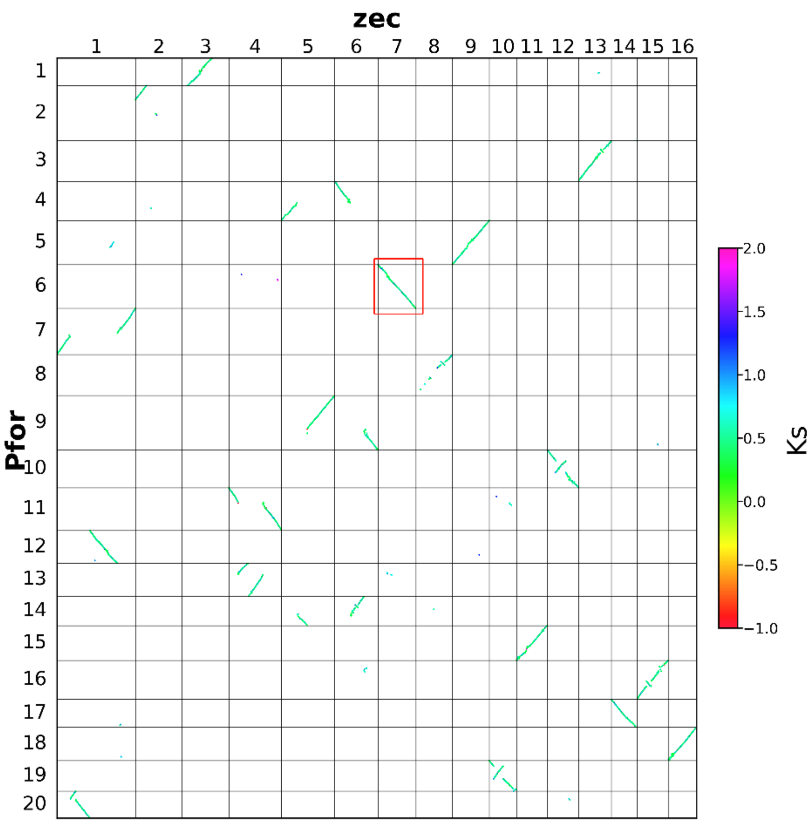

B

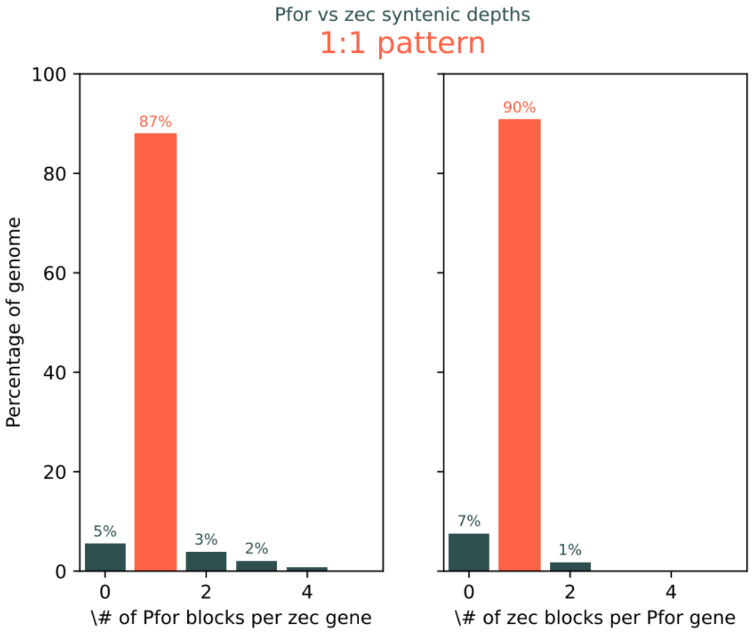

Fig. S30. Depth analysis of syntenic blocks between *P. fortunei* (Pfor) and *L. philippensis* (zec). (A) Syntenic dot plot between Pfor and zec. The red box highlighted regions with a 1:1 orthologous gene ratio between Pfor and zec. (B) The ratio of syntenic depth between Pfor and zec. Syntenic blocks of Pfor per zec gene (left) and syntenic blocks of zec per Pfor gene (right) are shown suggesting a clear 1:1 pattern of orthologous gene ratio.

A

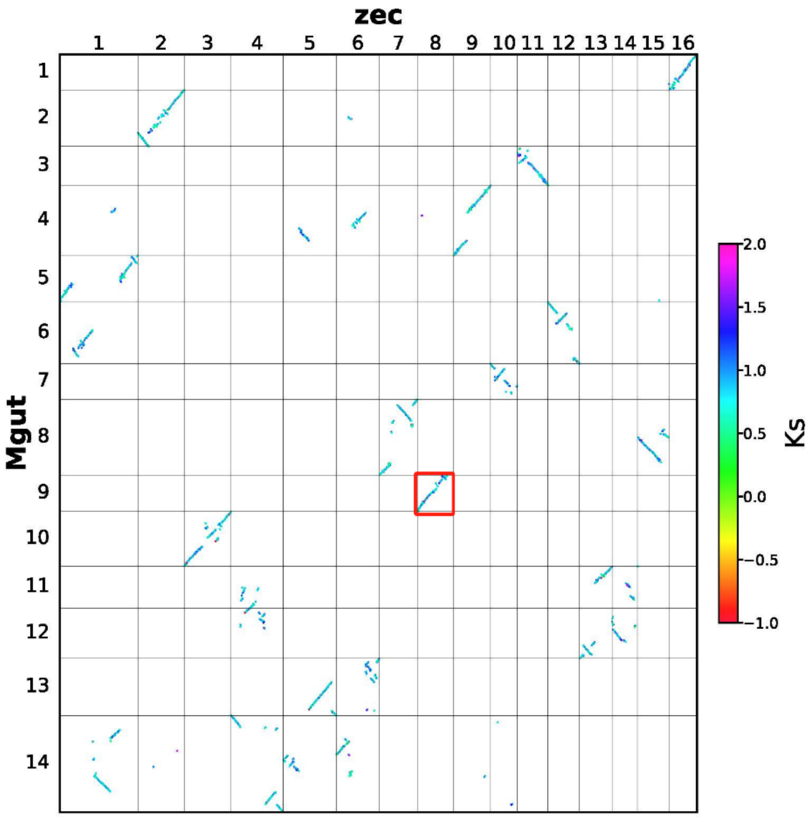

B

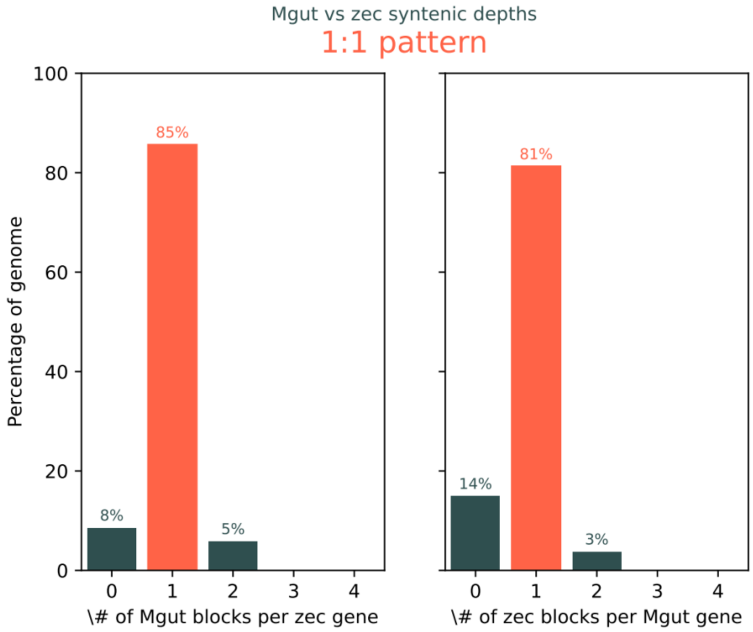

Fig. S31. Depth analysis of syntenic blocks between *M. guttatus* (Mgut) and *L. philippensis* (zec). (A) Syntenic dot plot between Mgut and zec. The red box highlighted regions with a 1:1 orthologous ratio between Mgut and zec. (B) The ratio of syntenic depth between Pfor and zec. Syntenic blocks of Mgut per zec gene (left) and syntenic blocks of zec per Mgut gene (right) are shown suggesting a clear 1:1 pattern of orthologous ratio.

A

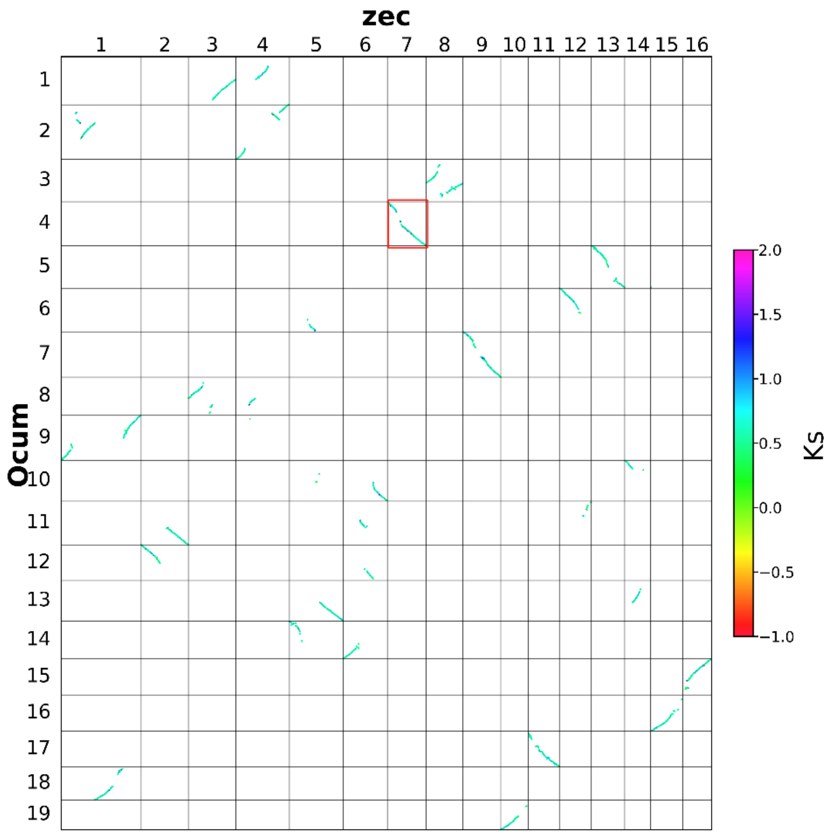

B

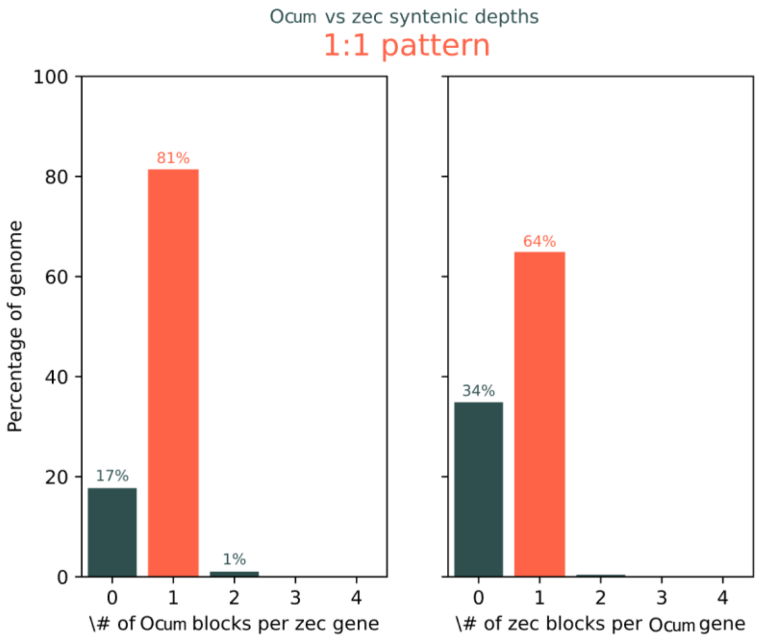

Fig. S32. Depth analysis of syntenic blocks between *O. cumana* (Ocum) and *L. philippensis* (zec). (A) Syntenic dot plot between Ocum and zec. Red box highlighted regions with 1:1 orthologous gene ratio between Ocum and zec. (B) Ratio of syntenic depth between Ocum and zec. Syntenic blocks of Ocum per zec gene (left) and syntenic blocks of zec per Ocum gene (right) are shown suggesting a clear 1:1 pattern of orthologous gene ratio.

A

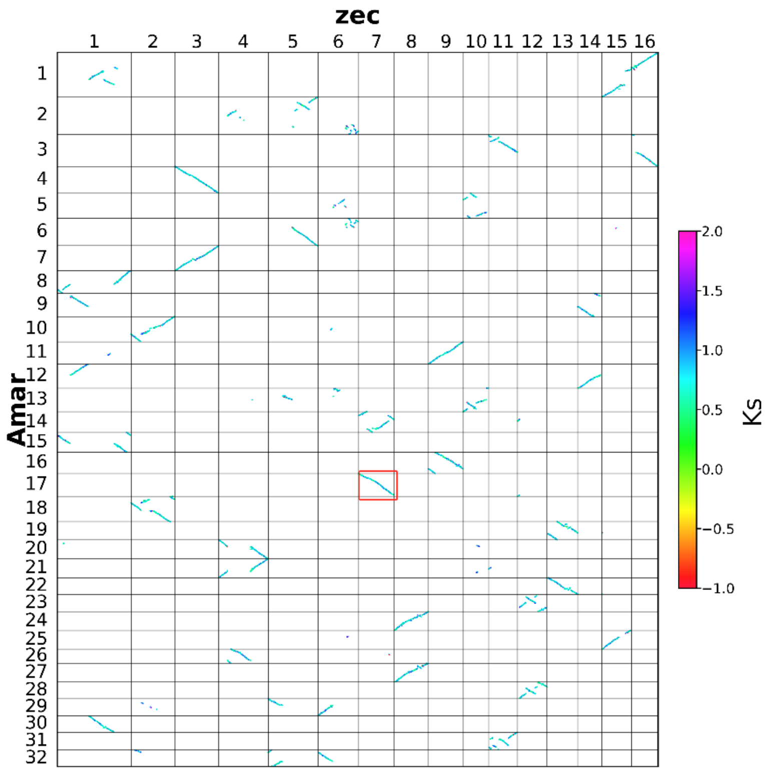

B

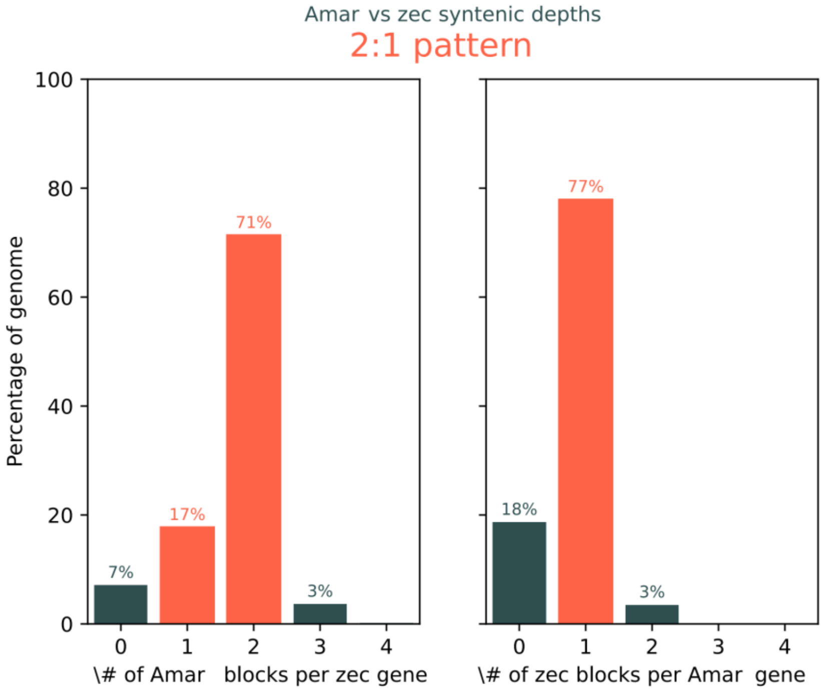

Fig. S33. Depth analysis of syntenic blocks between *A. marina* (Amar) and *L. philippensis* (zec). (A) Syntenic dot plot between Amar and zec. The red box highlighted regions with a 2:1 orthologous gene ratio between Amar and zec. (B) The ratio of syntenic depth between Amar and zec. Syntenic blocks of Amar per zec gene (left) and syntenic blocks of zec per Amar gene (right) are shown suggesting a clear 2:1 pattern of orthologous gene ratio.

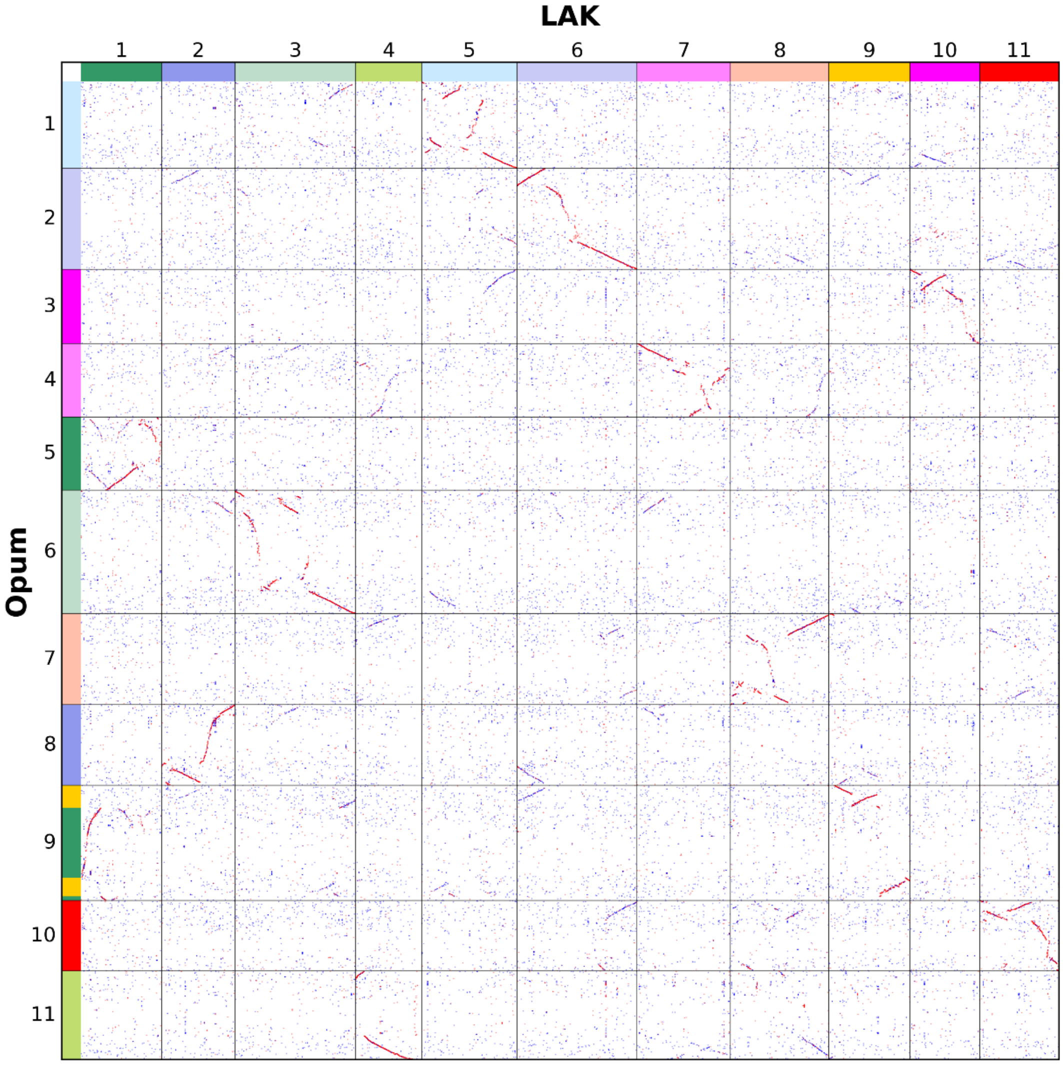

Fig. S34. Syntenic dot plot between *O. pumila* (Opum) and Lamiales ancestral karyotype (LAK). If the anchor gene pairs are the best BLAST hits among the genomes, they are plotted as red dots; otherwise, they are shown in blue dots.

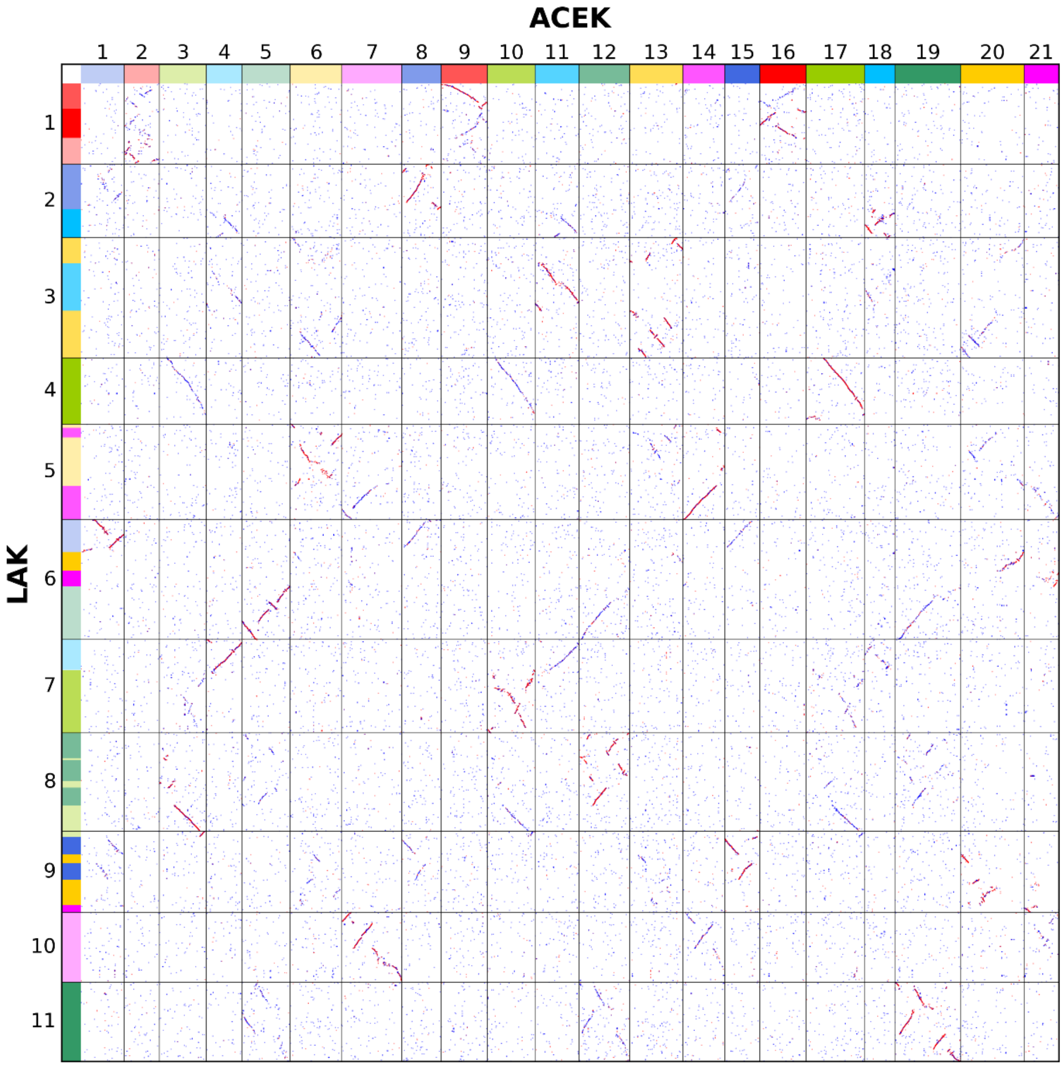

Fig. S35. Syntenic dot plot between LAK and the Ancestral Core Eudicot Karyotypes (ACEK). If the anchor gene pairs are the best BLAST hits among the genomes, they are plotted as red dots; otherwise, they are shown in blue dots.

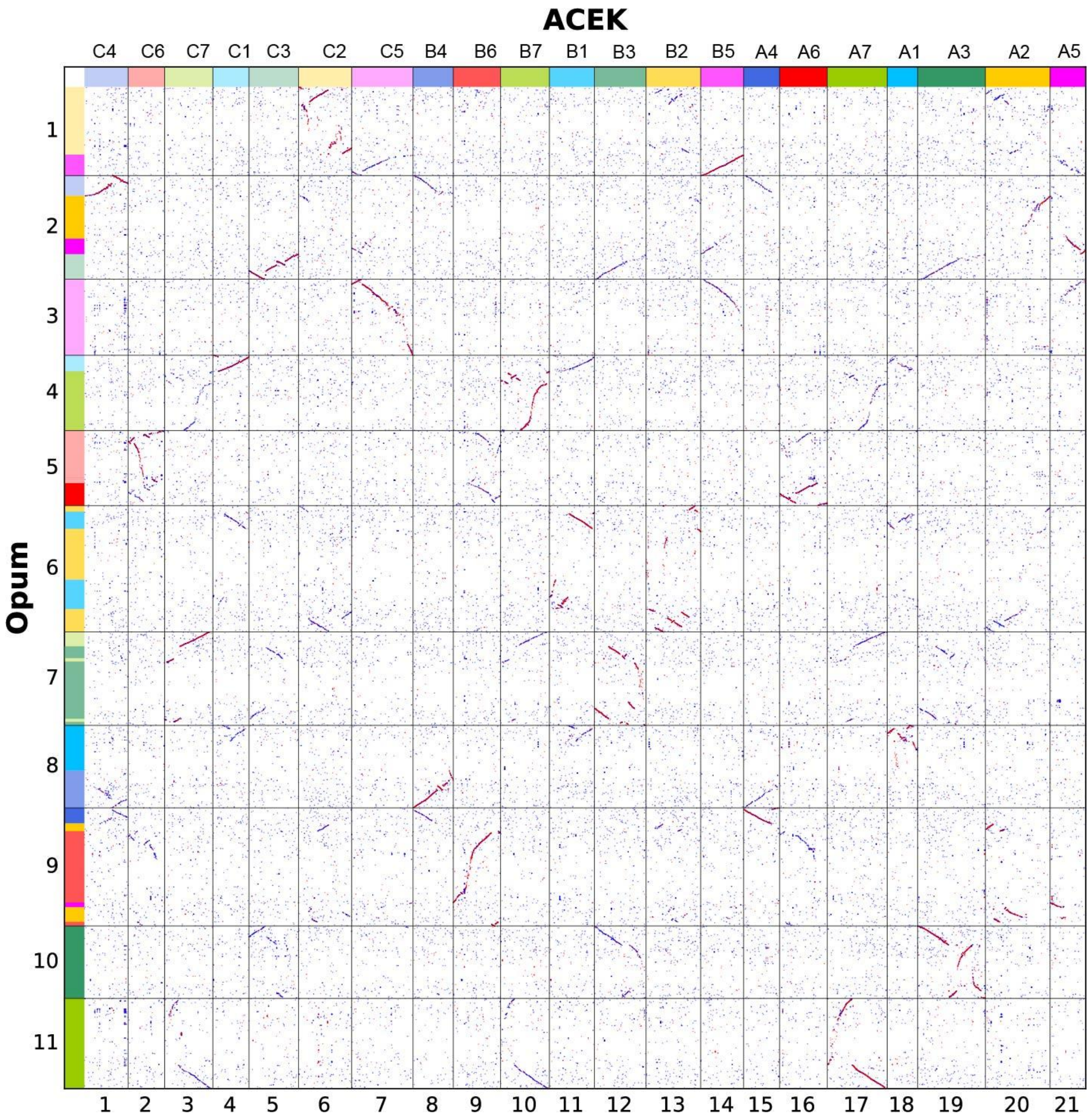

Fig. S36. Syntenic dot plot between *O. pumila* (Opum) and the ACEK. If the anchor gene pairs are the best BLAST hits among the genomes, they are plotted as red dots; otherwise, they are shown in blue dots.

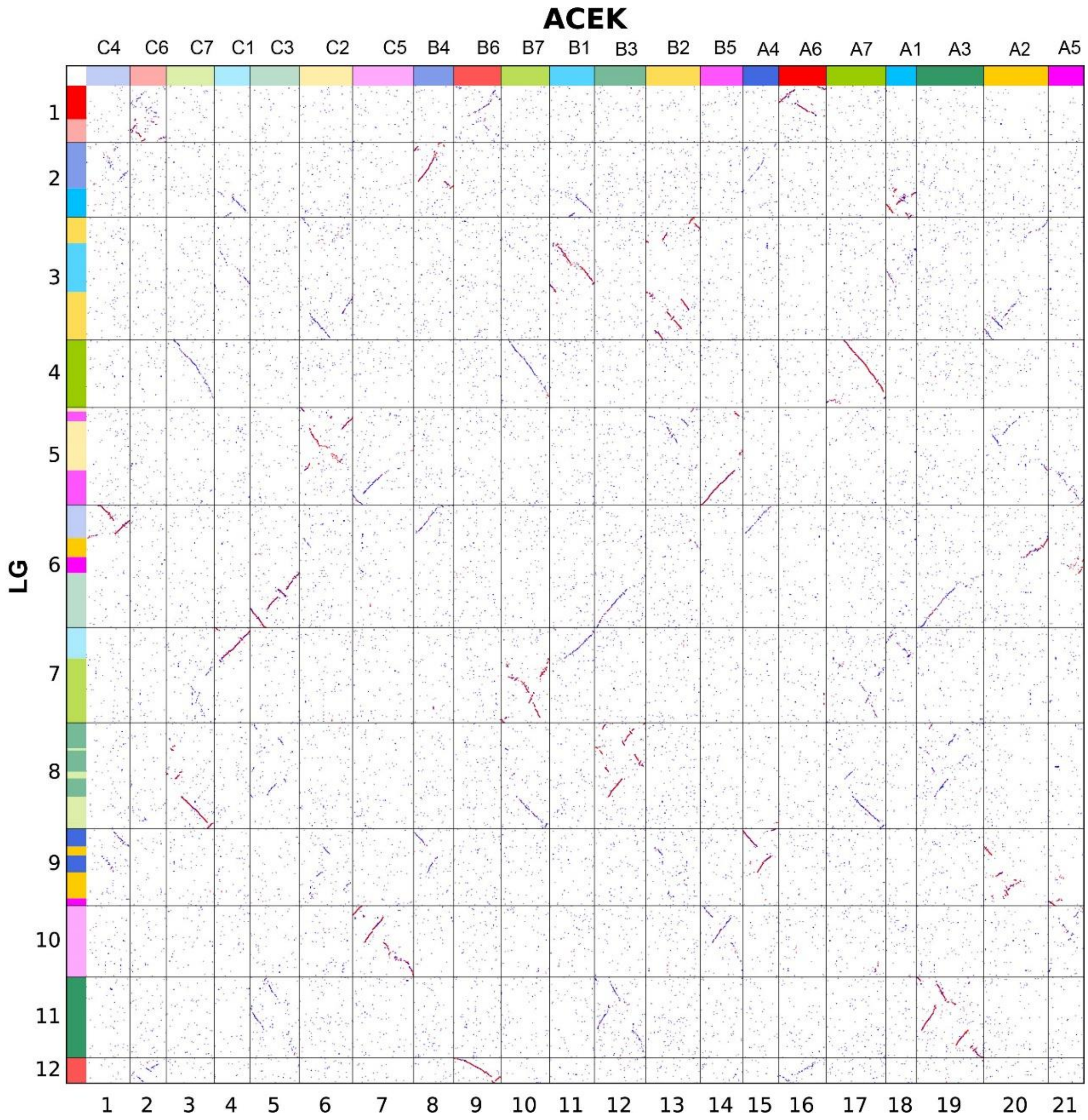

Fig. S37 Syntenic dot plot between ancestral karyotype of Lamiales and Gentianales orders (LG) and the ACEK. If the anchor gene pairs are the best BLAST hits among the genomes, they are plotted as red dots; otherwise, they are shown in blue dots.

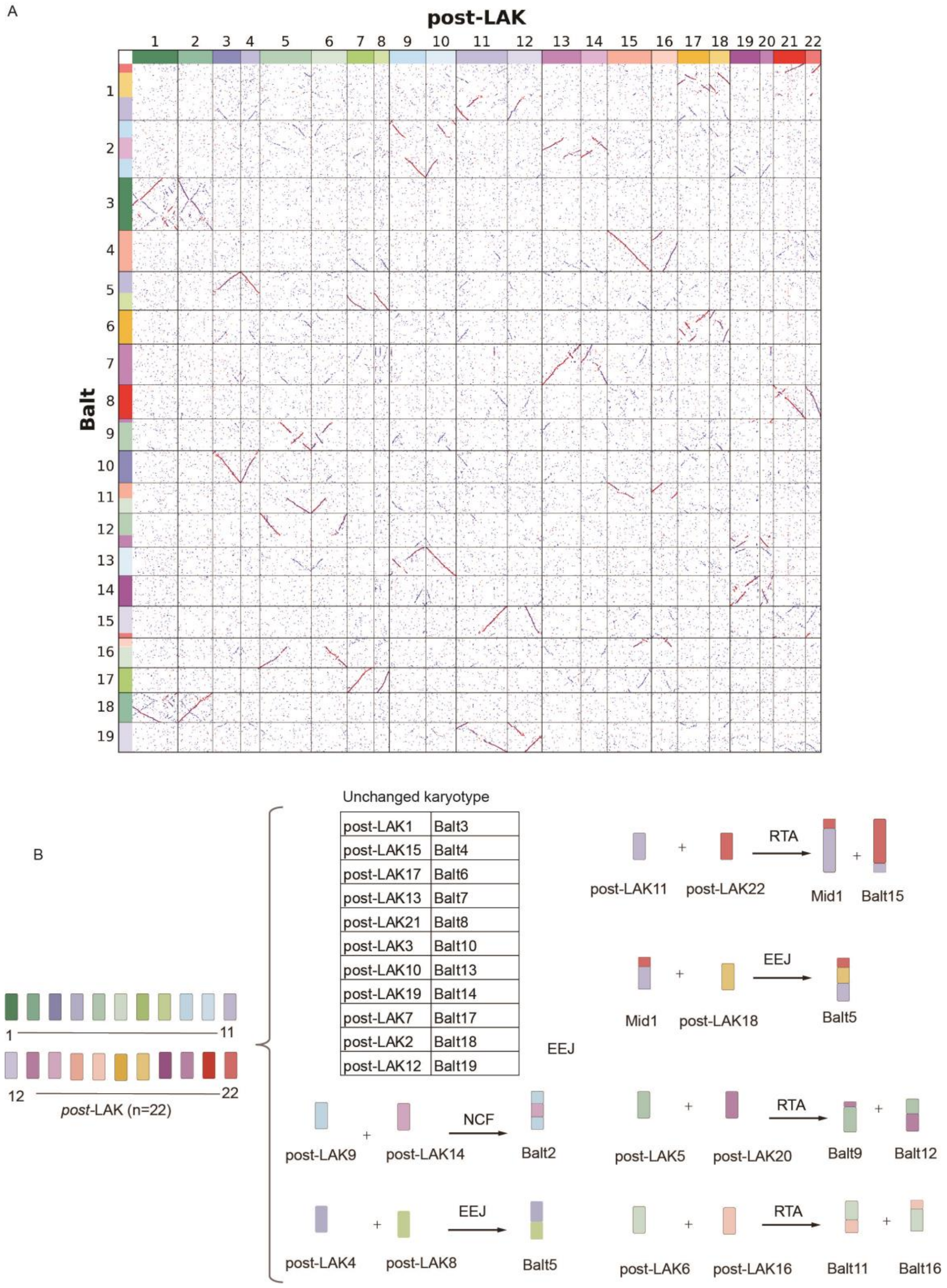

Fig. S38. Karyotype evolutionary trajectories of Post-LAK in *B. alternifolia* (Balt). (A) Syntenic dot plot between Balt and post-LAK. If the anchor gene pairs are the best BLAST hits among the genomes, they are plotted as red dots; otherwise, they are shown in blue dots. (B) Diagrammatic representation of karyotype evolution in Balt.

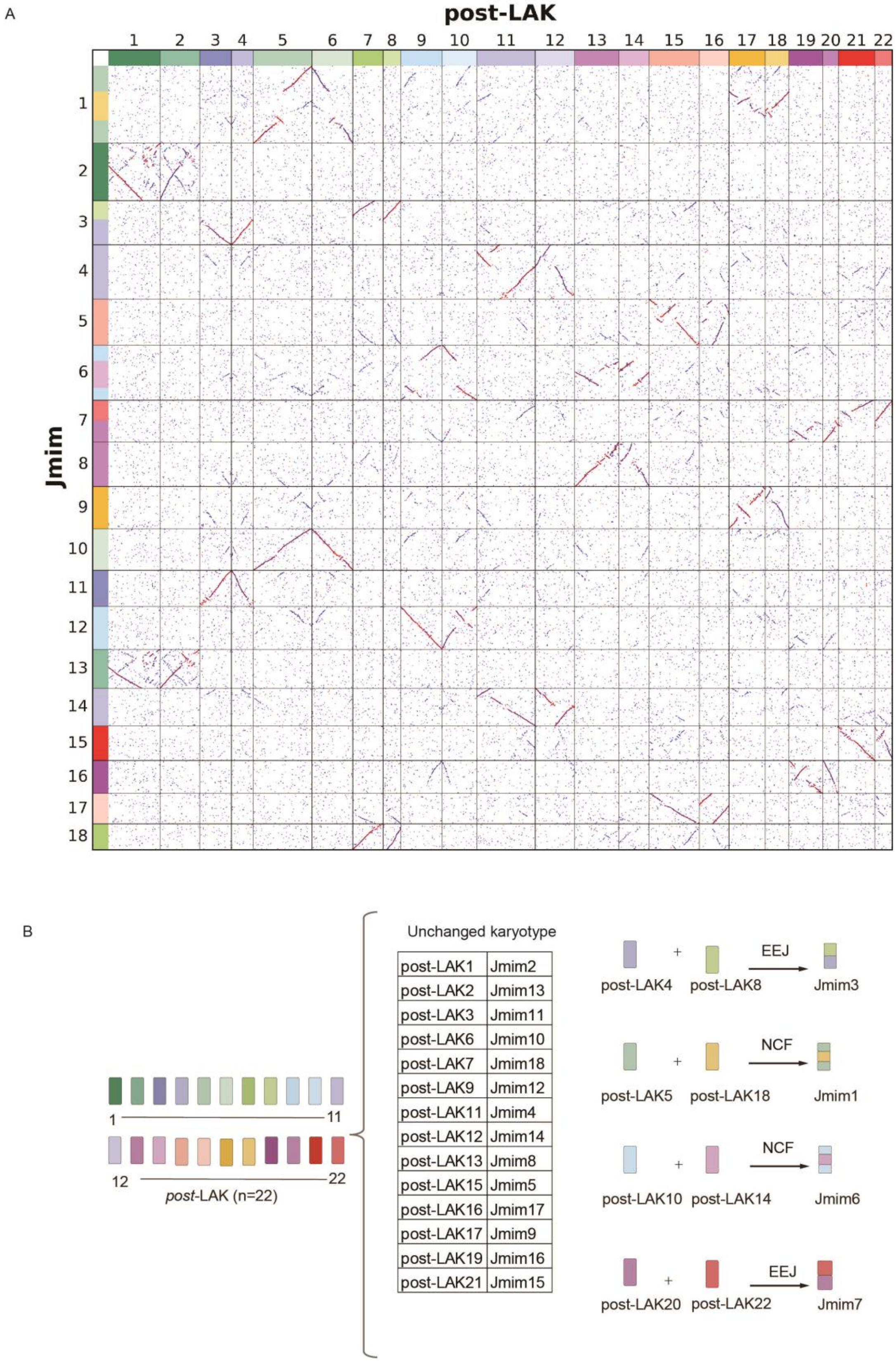

Fig. S39. Karyotype evolutionary trajectories of Post-LAK in *J. mimosifolia* (Jmim). (A) Syntenic dot plot between Jmim and post-LAK. If the anchor gene pairs are the best BLAST hits among the genomes, they are plotted as red dots; otherwise, they are shown in blue dots. B. Diagrammatic representation of karyotype evolution in Jmim.

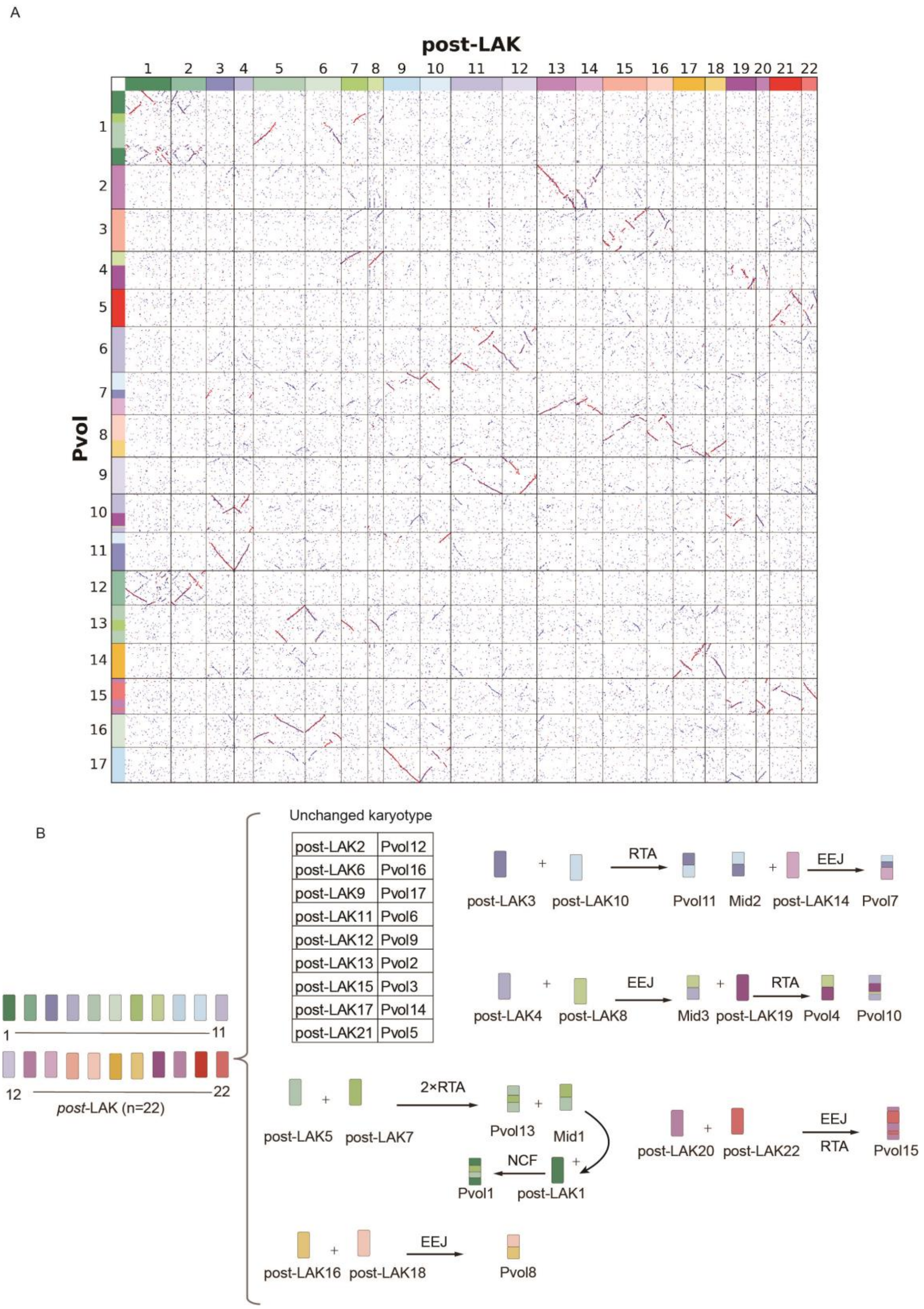

post-LAK2	Pvol12
post-LAK6	Pvol16
post-LAK9	Pvol17
post-LAK11	Pvol6
post-LAK12	Pvol9
post-LAK13	Pvol2
post-LAK15	Pvol3
post-LAK17	Pvol14
post-LAK21	Pvol5

Fig. S40. Karyotype evolutionary trajectories of Post-LAK in *P. volubilis* (Pvol). (A) Syntenic dot plot between Pvol and post-LAK. If the anchor gene pairs are the best BLAST hits among the genomes, they are plotted as red dots; otherwise, they are shown in blue dots. (B) Diagrammatic representation of karyotype evolution in Pvol.

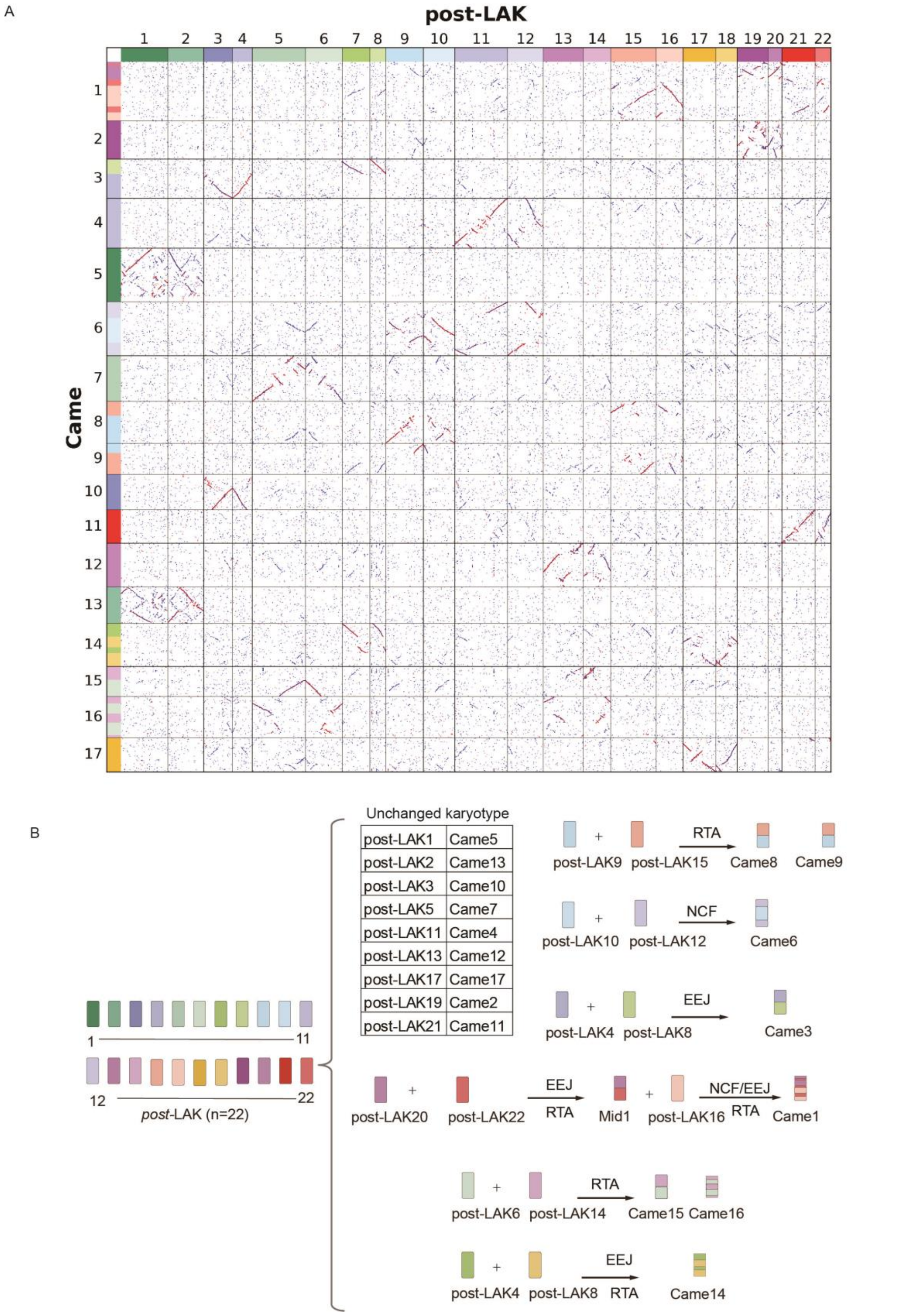

Fig. S41. Karyotype evolutionary trajectories of Post-LAK in *C. americana* (Came). (A) Syntenic dot plot between Came and post-LAK. If the anchor gene pairs are the best BLAST hits among the genomes, they are plotted as red dots; otherwise, they are shown in blue dots. (B) Diagrammatic representation of karyotype evolution in Came.

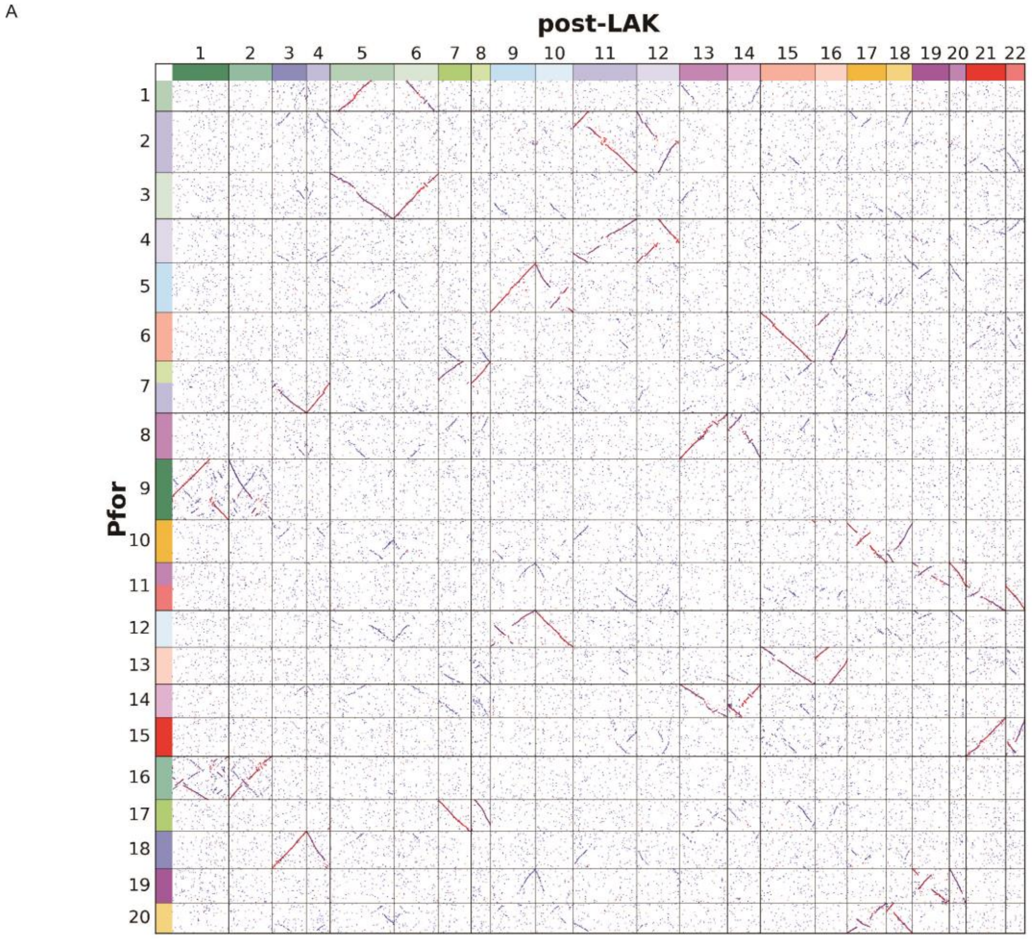

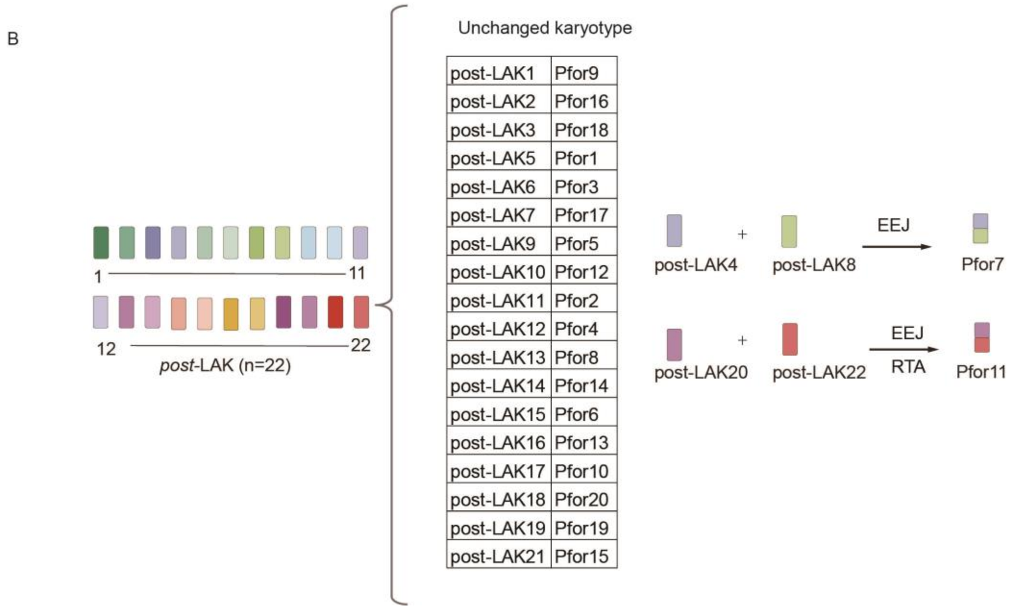

Fig. S42. Karyotype evolutionary trajectories of Post-LAK in *P. fortunei* (Pfor). (A) Syntenic dot plot between Pfor and post-LAK. If the anchor gene pairs are the best BLAST hits among the genomes, they are plotted as red dots; otherwise, they are shown in blue dots. (B) Diagrammatic representation of karyotype evolution in Pfor.

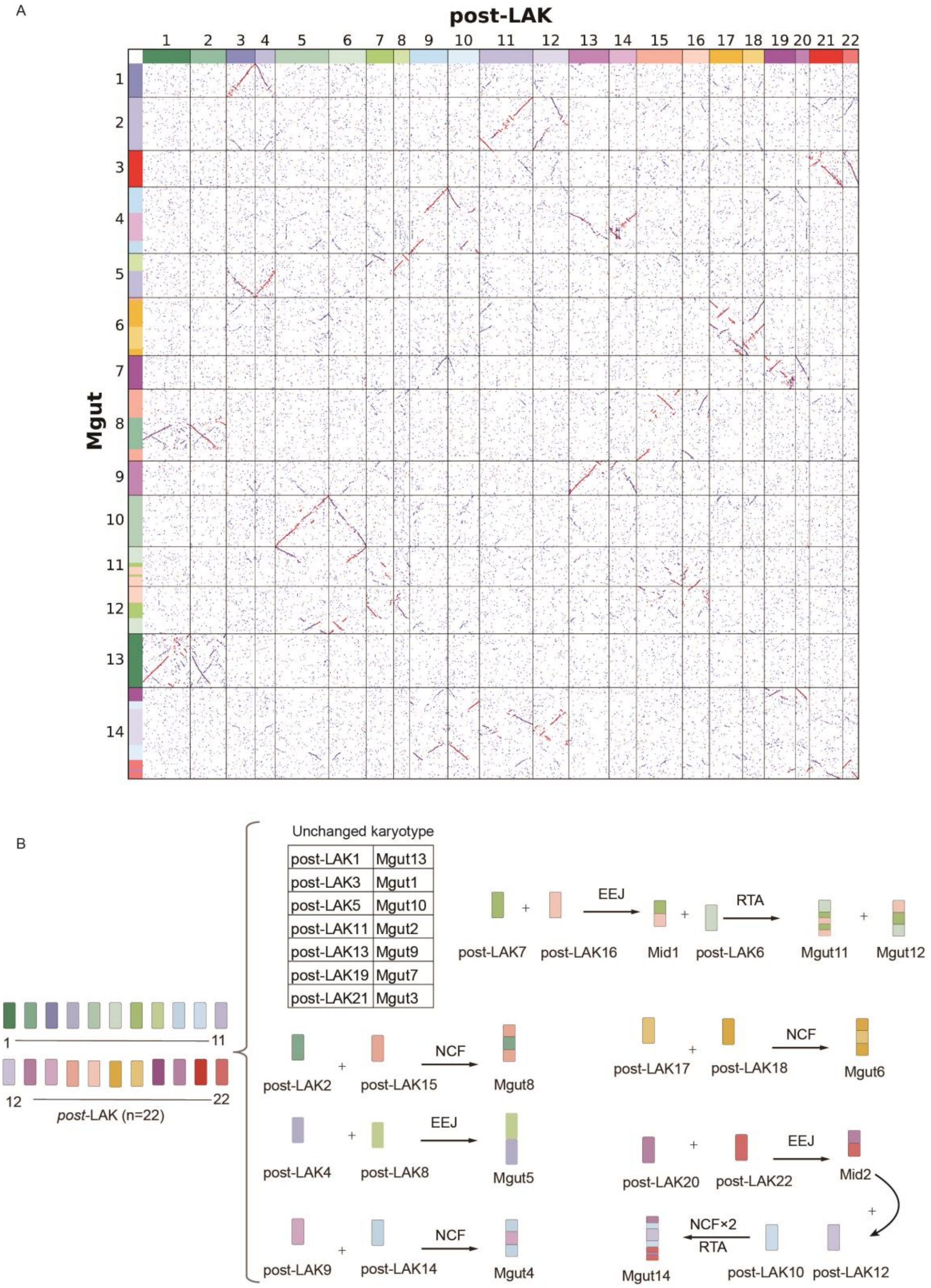

Fig. S43. Karyotype evolutionary trajectories of Post-LAK in *M. guttatus* (Mgut). (A) Syntenic dot plot between Mgut and post-LAK. If the anchor gene pairs are the best BLAST hits among the genomes, they are plotted as red dots; otherwise, they are shown in blue dots. (B) Diagrammatic representation of karyotype evolution in Mgut.

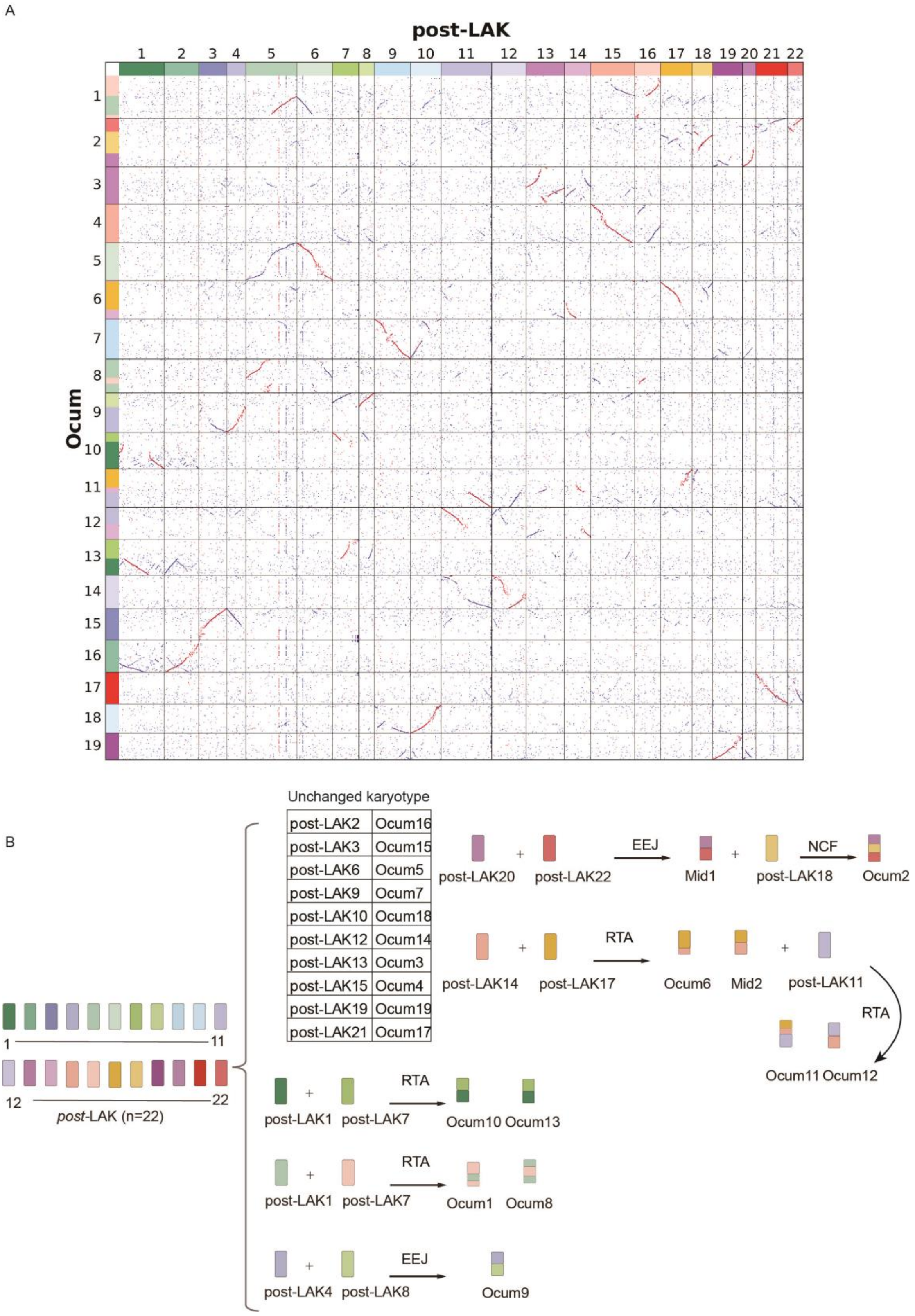

Fig. S44. Karyotype evolutionary trajectories of Post-LAK in *O. cumana* (Ocum). (A) Syntenic dot plot between Ocum and post-LAK. If the anchor gene pairs are the best BLAST hits among the genomes, they are plotted as red dots; otherwise, they are shown in blue dots. (B) Diagrammatic representation of karyotype evolution in Ocum.

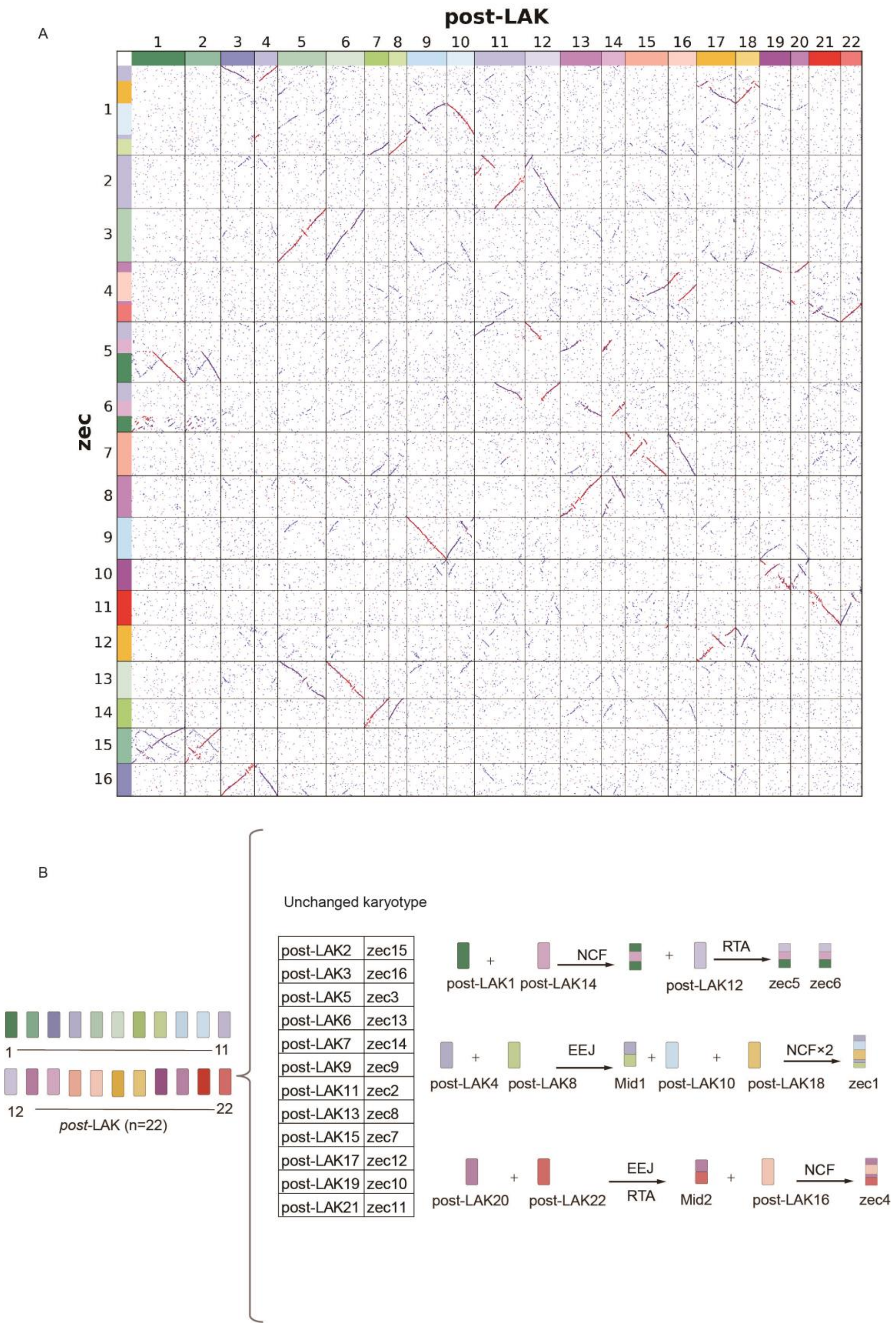

Fig. S45. Karyotype evolutionary trajectories of Post-LAK in *L. philippensis* (zec). (A) Syntenic dot plot between zec and post-LAK. If the anchor gene pairs are the best BLAST hits among the genomes, they are plotted as red dots; otherwise, they are shown in blue dots. (B) Diagrammatic representation of karyotype evolution in zec.

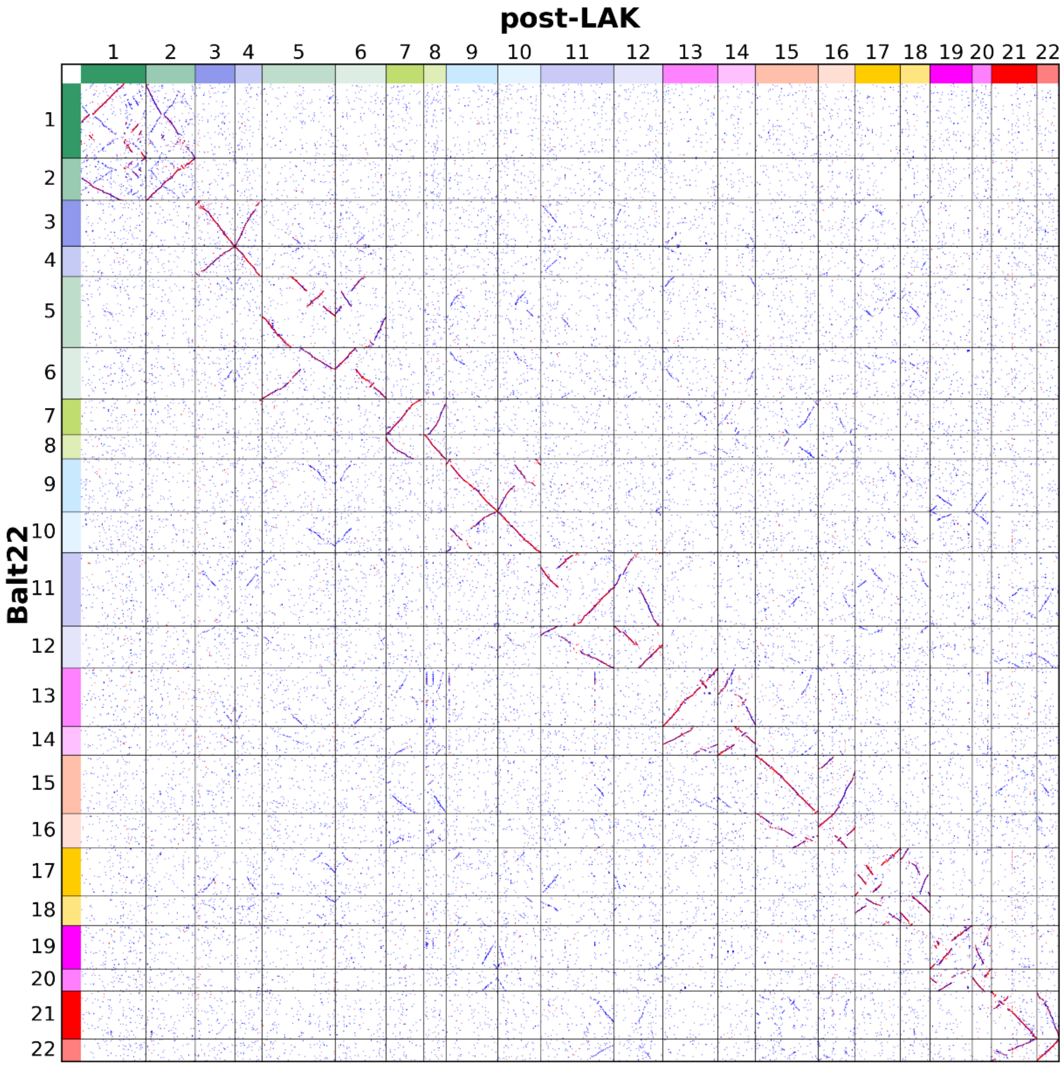

Fig. S46. Syntenic dot plot between the subgenomes of *B. alternifolia* (Balt22) and post-LAK genome. If the anchor gene pairs are the best BLAST hits among the genomes, they are plotted as red dots; otherwise, they are shown in blue dots.

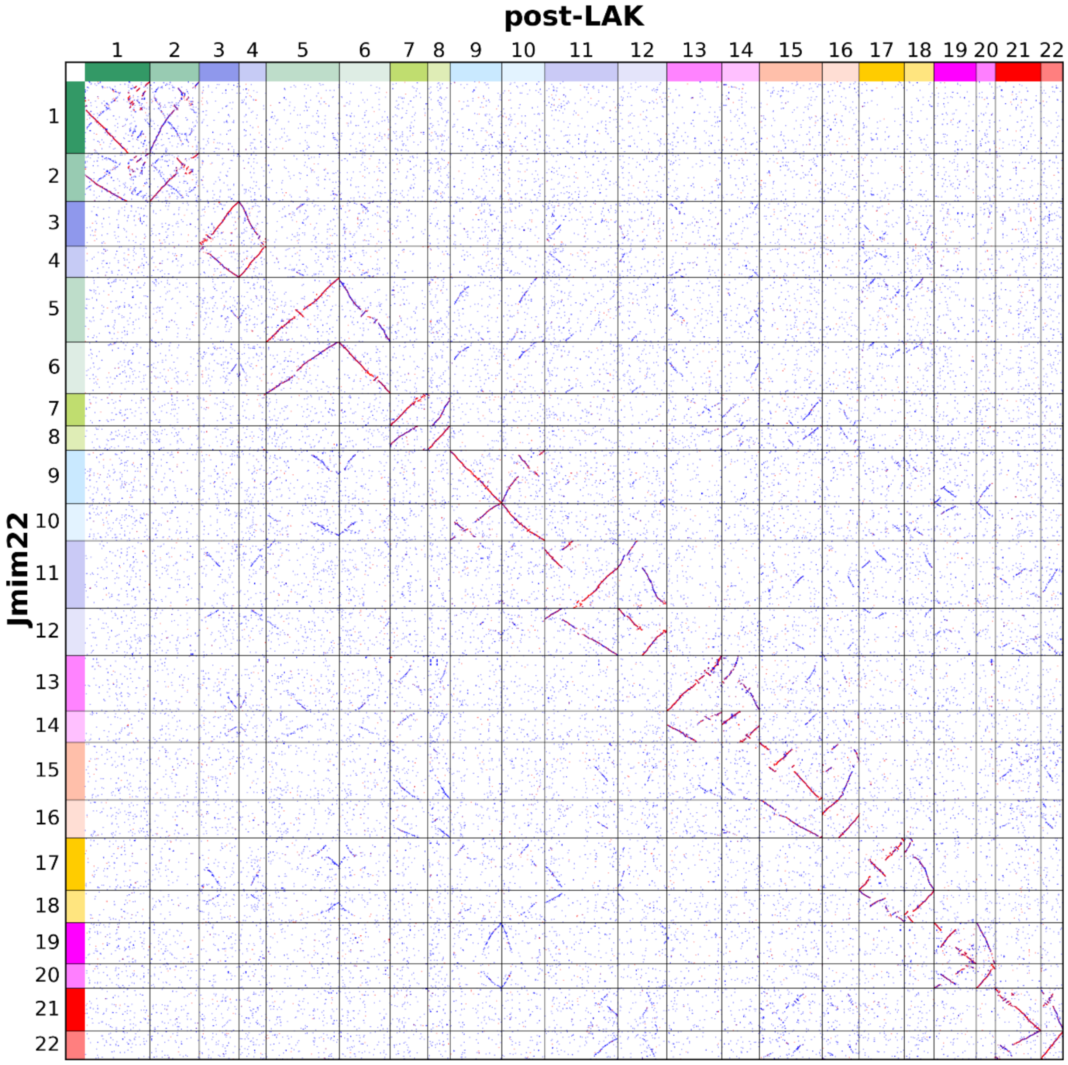

Fig. S47. Syntenic dot plot between the subgenomes of *J. mimosifolia* (Jmim) and post-LAK genome. If the anchor gene pairs are the best BLAST hits among the genomes, they are plotted as red dots; otherwise, they are shown in blue dots.

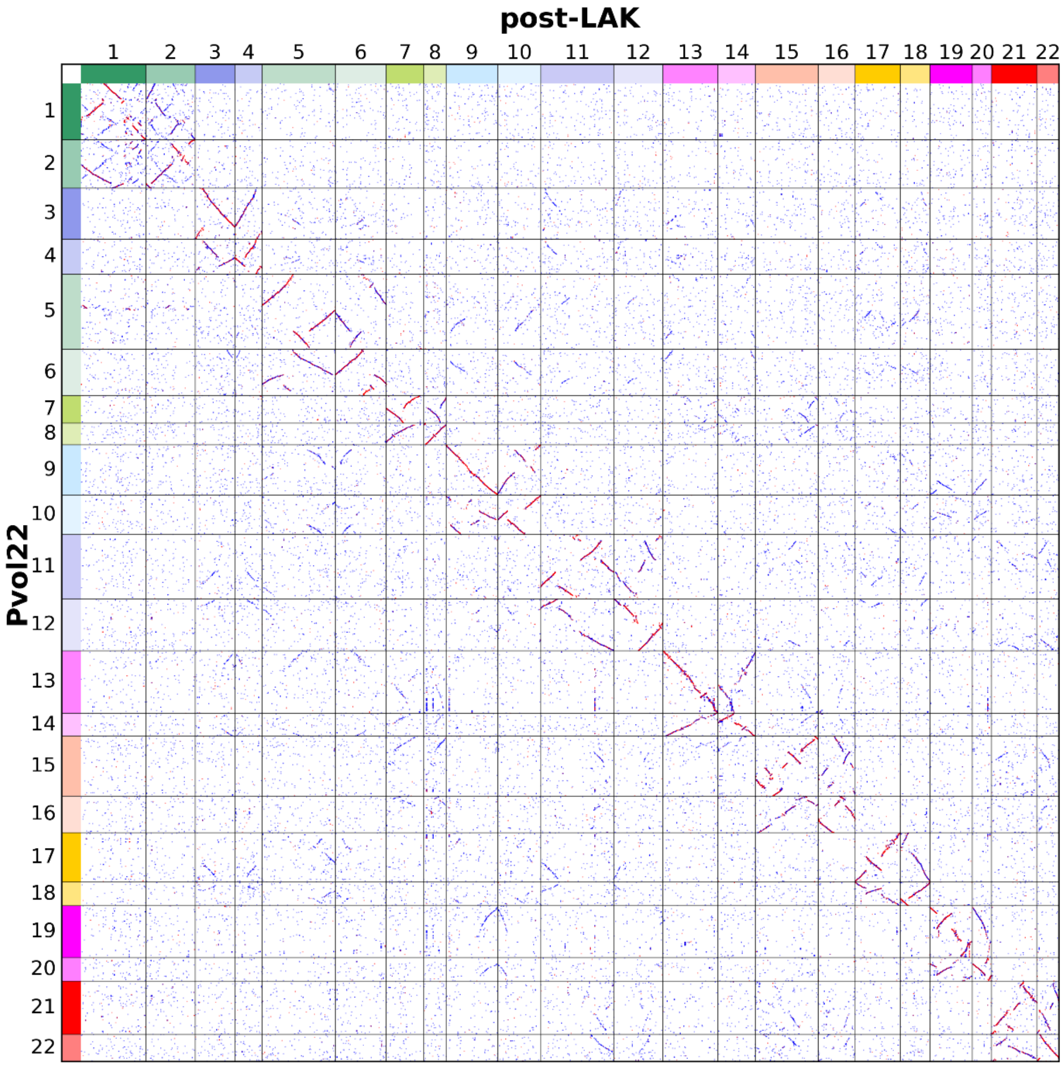

Fig. S48. Syntenic dot plot between the subgenomes of *P. volubilis* (Pvol22) and post-LAK genome. If the anchor gene pairs are the best BLAST hits among the genomes, they are plotted as red dots; otherwise, they are shown in blue dots.

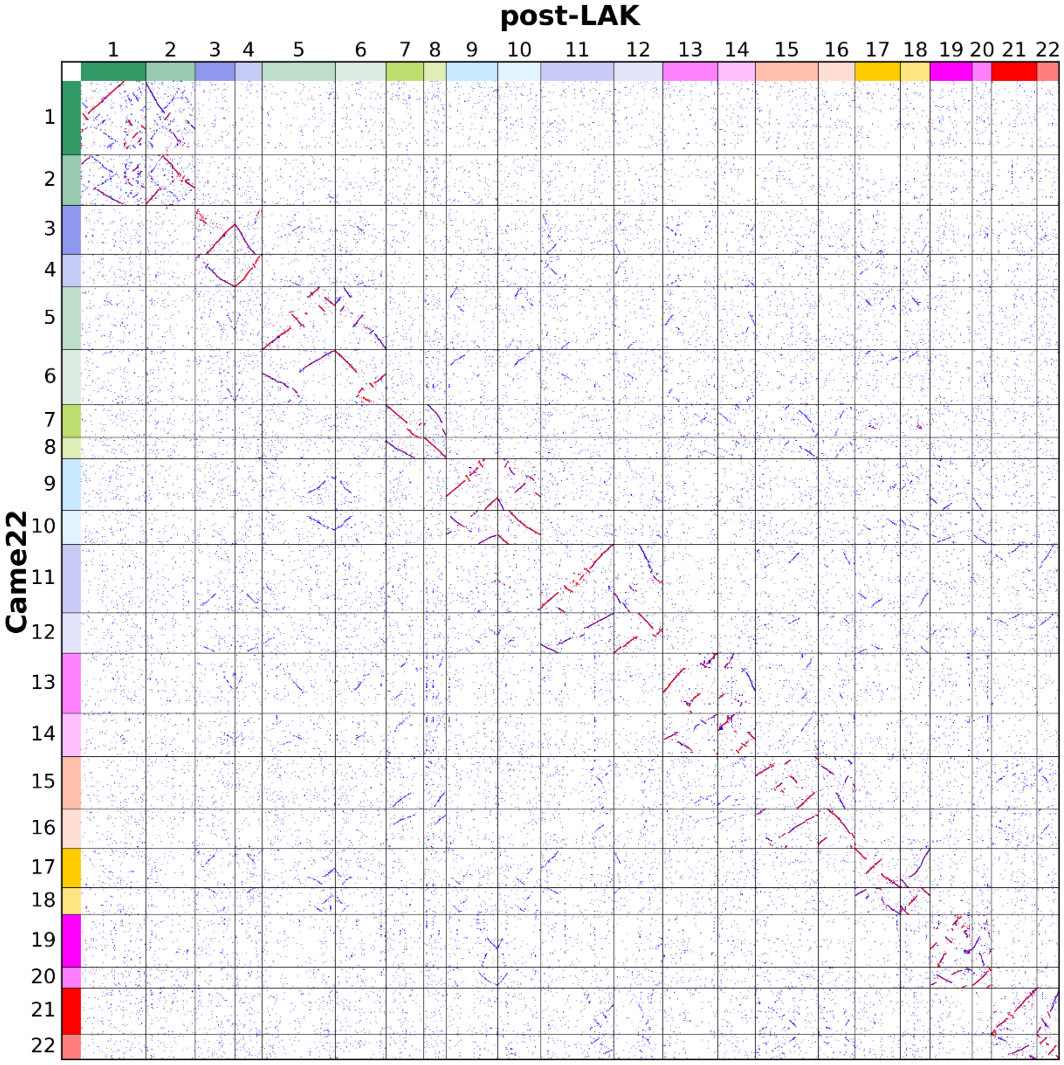

Fig. S49. Syntenic dot plot between the subgenomes of *C. americana* (Came22) and post-LAK genome. If the anchor gene pairs are the best BLAST hits among the genomes, they are plotted as red dots; otherwise, they are shown in blue dots.

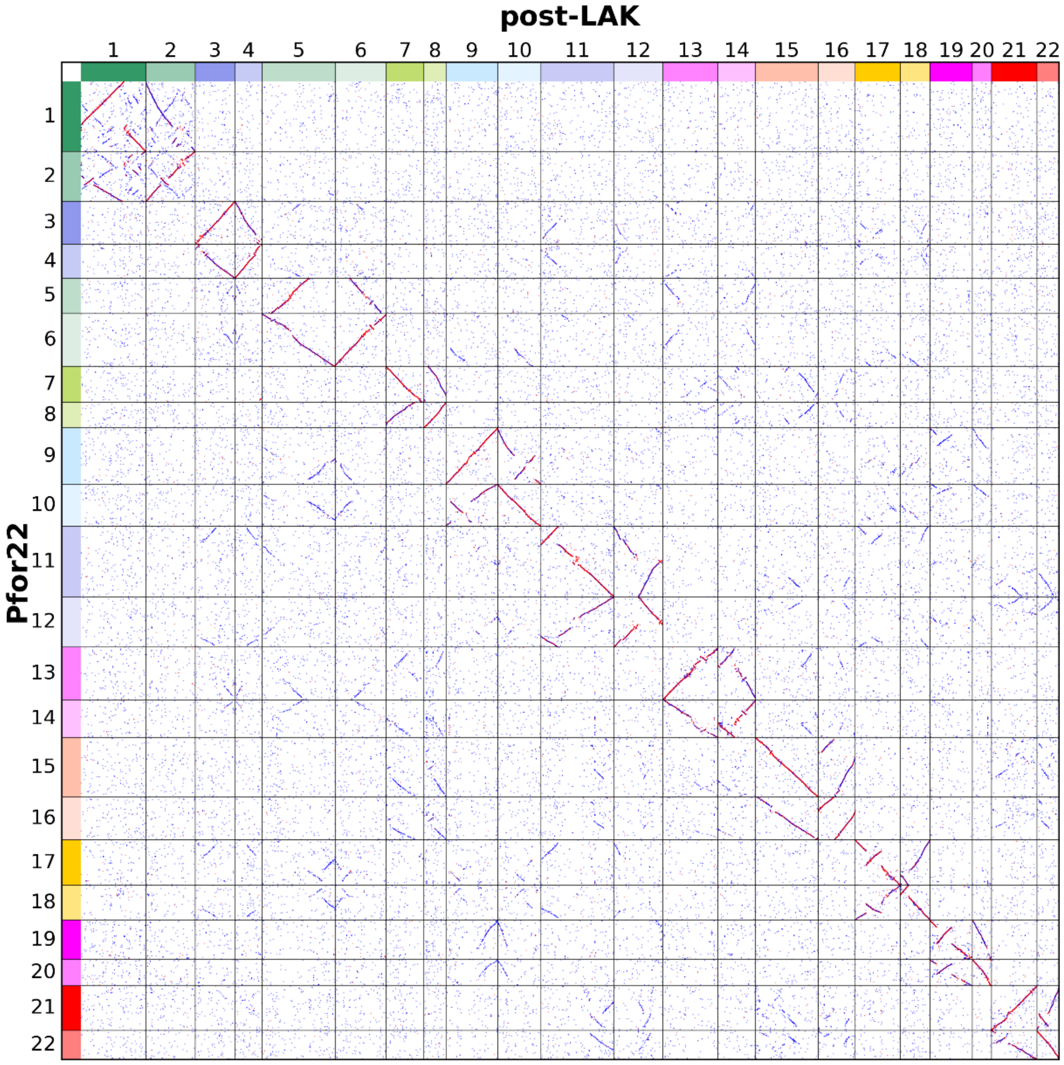

Fig. S50. Syntenic dot plot between the subgenomes of *P. fortunei* (Pfor22) and post-LAK genome. If the anchor gene pairs are the best BLAST hits among the genomes, they are plotted as red dots; otherwise, they are shown in blue dots.

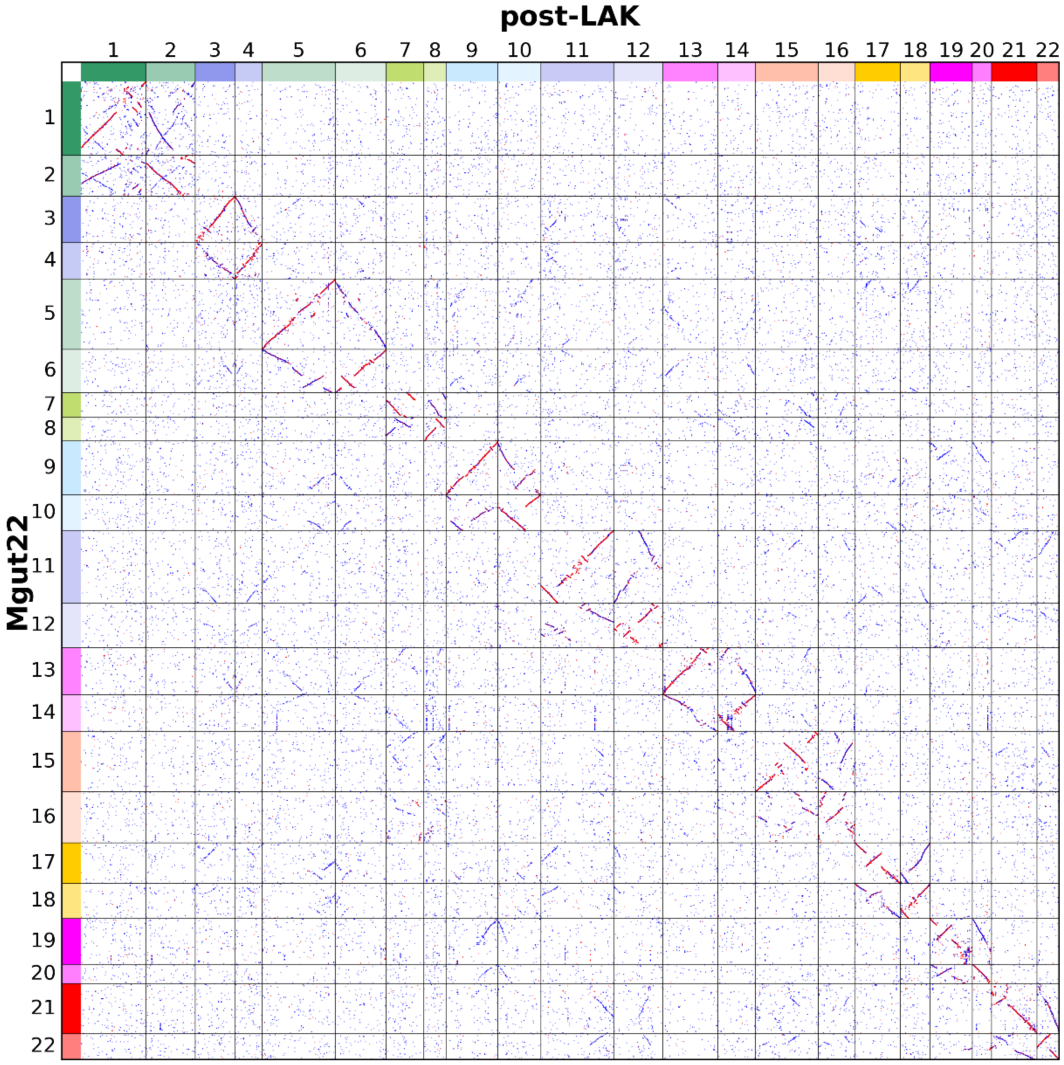

Fig. S51. Syntenic dot plot between the subgenomes of *M. guttatus* (Mgut22) and post-LAK genome. If the anchor gene pairs are the best BLAST hits among the genomes, they are plotted as red dots; otherwise, they are shown in blue dots.

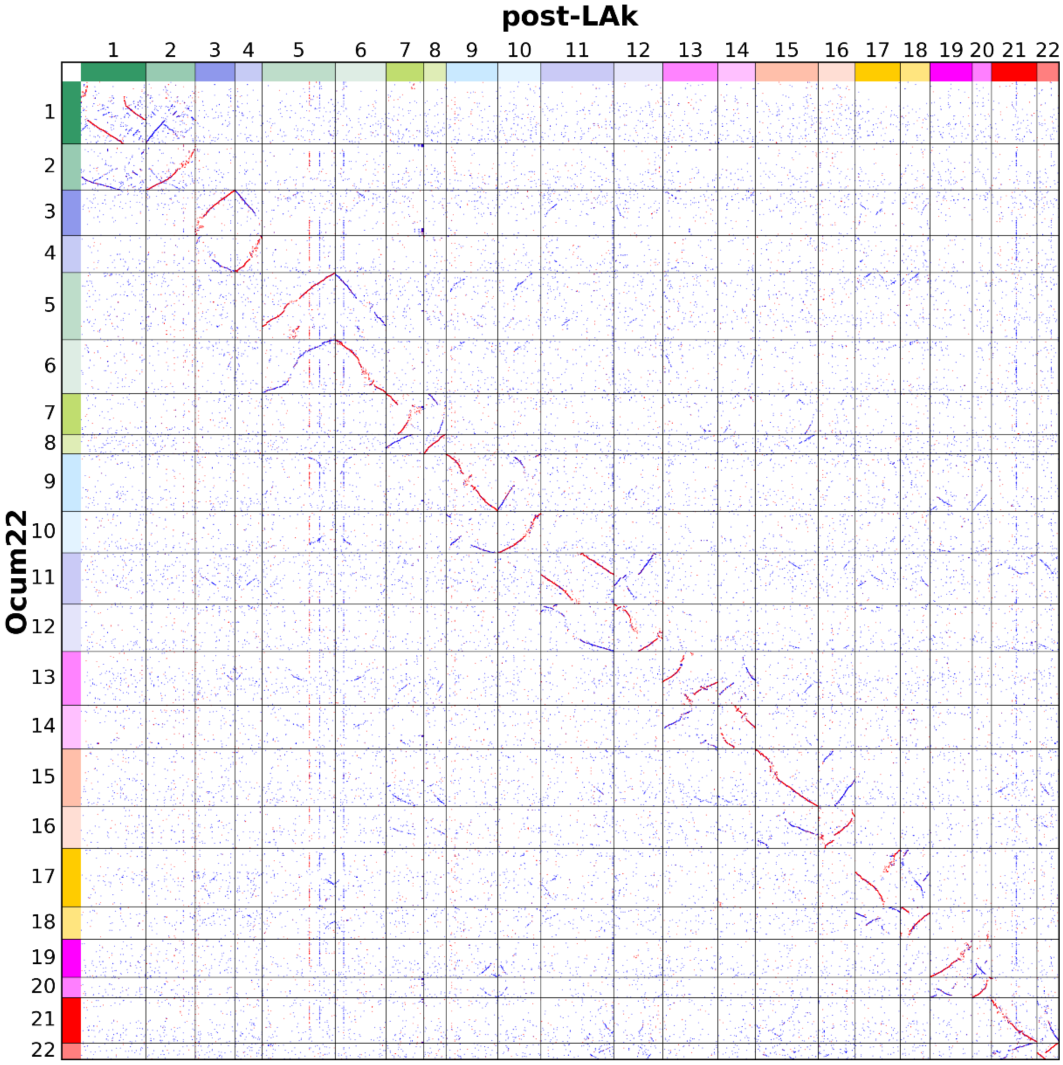

Fig. S52. Syntenic dot plot between the subgenomes of *O. cumana* (Ocum22) and post-LAK genome. If the anchor gene pairs are the best BLAST hits among the genomes, they are plotted as red dots; otherwise, they are shown in blue dots.

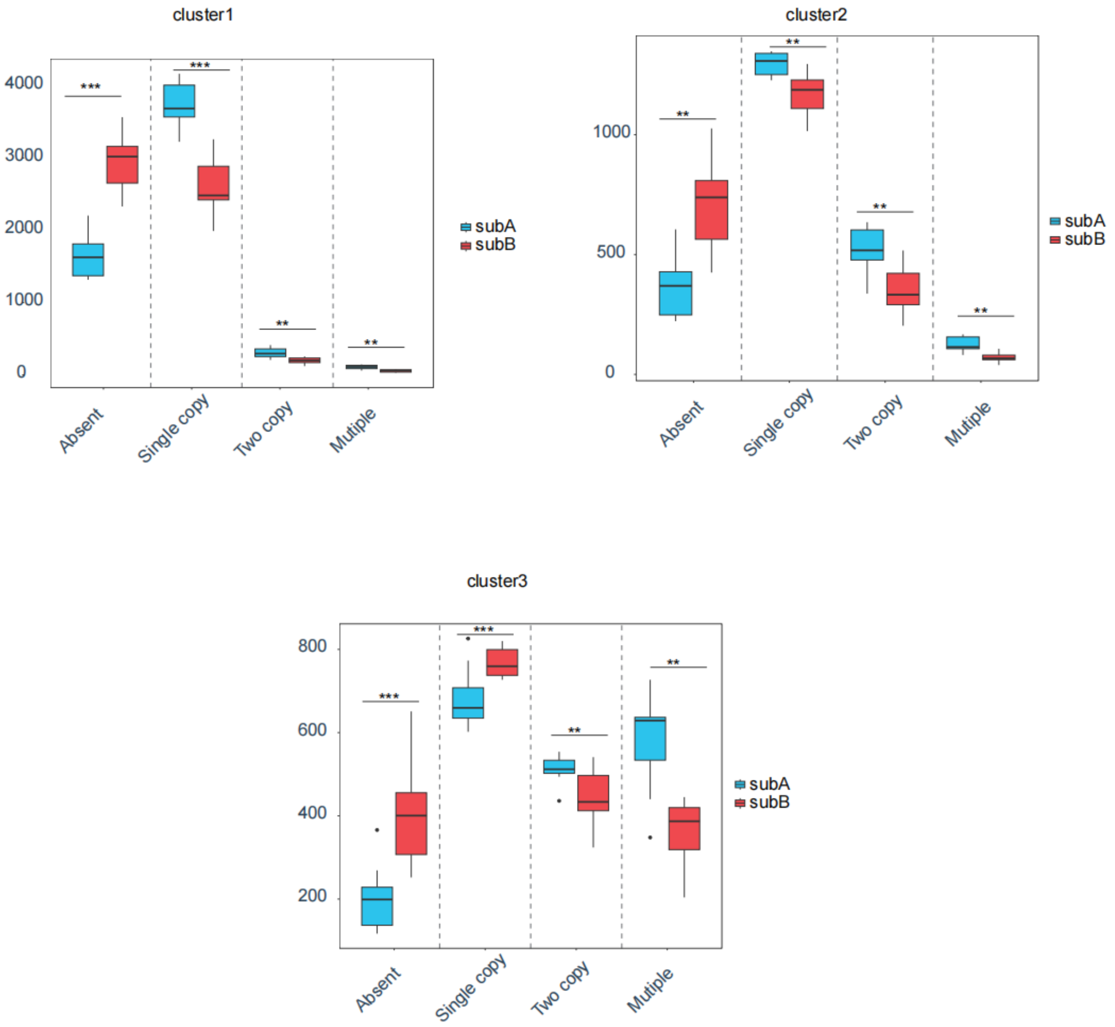

Fig. S53. Comparison and analysis of the core set of orthogroups (CSOs) of subgenomes within three clusters. Asterisk indicates a significant difference (One-sided *t*-test). If a *P* value is less than 0.05, it is flagged with one star (*). If a *P* value is less than 0.01, it is flagged with 2 stars (**). If a *P* value is less than 0.001, it is flagged with three stars (***)
