# Supplementary material for: Chromosome-level assembly of Lindenbergia philippensis and comparative genomic analyses shed light on genome evolution in Lamiales
Source: Front Plant Sci. 2024 Aug 2;15:1444234. doi: 10.3389/fpls.2024.1444234 (PMC11327160; doi:10.3389/fpls.2024.1444234)
Supplement: Supplementary file 3 [file DataSheet_2.pdf]

Note1 Inference of Lamiales ancestral karyotype (LAK)

The construction of LAK followed the method proposed by Sun, et al. (2022), and further details can be found in the gihub: https://github.com/SunPengChuan/wgdi-example/blob/main/Karyotype_Evolution.md. The process contains mainly three steps as follows:

Step1: WGD detect

The construction of LAK is based on the *L. philippensis* genome. We first used WGDI with the "-d" parameter to plot the dot plot within the *L. philippensis* genome. According to the mentioned result, *L. philippensis* experienced one WGD (L-WGD) after γ-WGT event, which means that the inter-sytenic depth is $6 = 2_{(\text{L-WGD})} \times 3_{(\gamma\text{-WGT})}$ (Figure 1). We focused solely on the best homologous gene produced from the L-WGD to reconstruct the LAK. In theory, there should be one paired superior homologous chromosome within *L. philippensis*. Following this assumption, the intact homologous chromosomes with the "Telomere-to-Telomere" trait could be considered as the potential LAK.

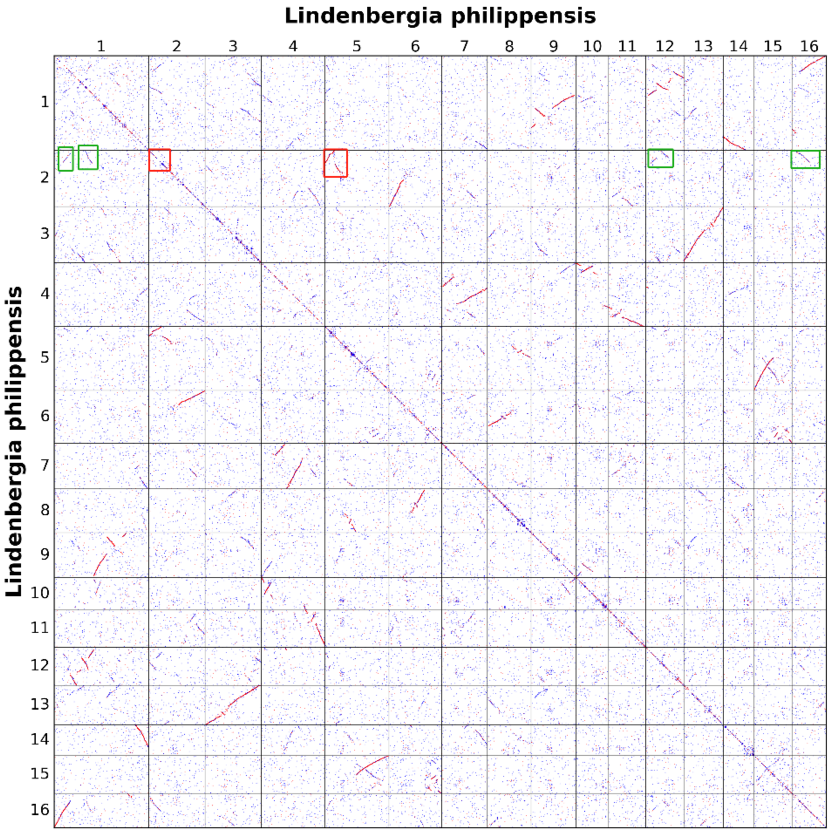

Figure 1. Inter-syntenic dot plot of *L. philippensis* genome. The red box represents the best homologous blocks, while the green box represents the next best homologous blocks. If the anchor gene pairs are the best BLAST hits among the genomes, they are plotted as red dots; otherwise, they are shown in blue dots.

Then, we adjusted the order of chromosomes in the lens file (required by WGDI) several times and plotted the homologous dot plot to keep the collinear blocks together as much as possible (Figure 2).

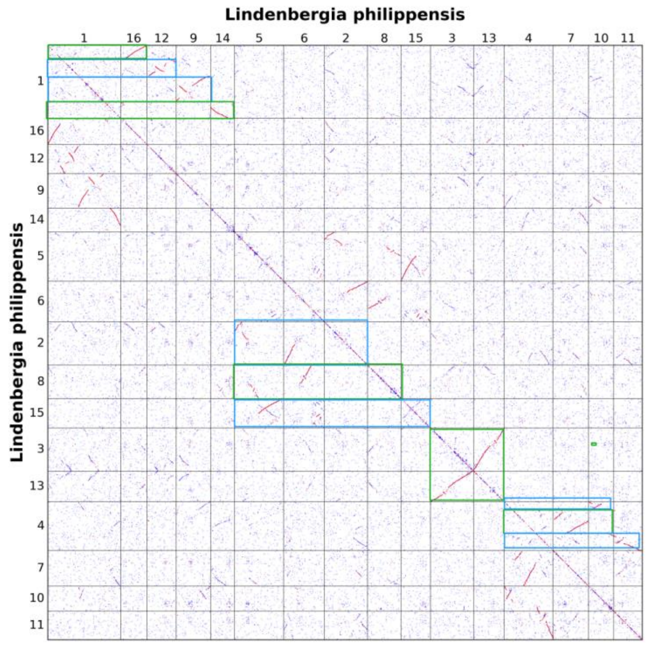

Figure 2. The adjusted inter-syntenic dot plot of *L. philippensis* genome. The green and blue boxes represent different clusters of the best homologous blocks

We separately extracted haplotypes with whole chromosomes as potential protochromosomes from different clusters. Totally, 11 protochromosome clusters were detected. Such as Chrs 1 and 16 in the green cluster, Chrs 1 and 12 in the blue cluster, Chrs 1 and 9 in the blue cluster, Chrs 1 and 14 in the green cluster, Chrs 2 and 5, 6 in the blue cluster, Chrs 8 and 5, 6 in the green cluster, Chrs 15 and 5, 6 in the blue cluster, Chrs 3 and 13 in the green cluster, Chrs 4 and 10 in the blue cluster, Chrs 4 and 7 in the green cluster and Chrs 4 and 11 in the blue cluster.

Step2: reconstructed of ancestral karyotype

We selected an intact protochromosome from each cluster and marked them with different colors and groups. Here the result file is as follows.

15	20584726	1222	#339966
16	18019699	1108	#8f98ec
3	27774833	1823	#beddca
14	20739130	1000	#c0dd6f
9	23302763	1446	#c9e9ff
2	27899668	1819	#cacaf6
8	23511984	1414	#ff83ff
7	24149717	1486	#ffbfaa
12	21313475	1227	#FFCC00
10	22821227	1059	fuchsia
11	22019011	1197	red

Then, we used WGDI with the "-ak" parameter to construct the LAK.

The ancestor file of LAK is as follows:

1	1	1222	#339966
2	1	1108	#8f98ec

3	1	1823	#beddca
4	1	1000	#c0dd6f
5	1	1446	#c9e9ff
6	1	1819	#cacaf6
7	1	1414	#ff83ff
8	1	1486	#ffbfaa
9	1	1227	#FFCC00
10	1	1059	fuchsia
11	1	1197	red

Most of the genes of *L. philippensis* should be derived from LAK, we further mapped LAK to *L. philippensis* to avoid other proto-chromosomes from being ignored. We first used WGDI with the "-d" parameter to plot a homologous dot plot between *L. philippensis* with LAK as follows (Figure 3).

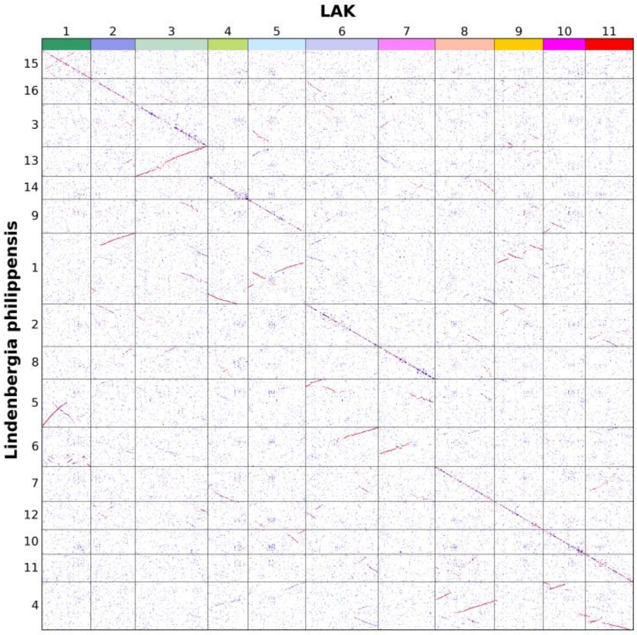

Figure 3. The syntenic dot plot of *L. philippensis* and LAK genome.

Step3: demonstrate the correctness of ancestral karyotype

Then, we also used WGDI with the "-km" parameter to obtain the mapping of LAK. Finally, we again used WGDI with the "-d" parameter and added the ancestor_left to plot the homologous dot plot.

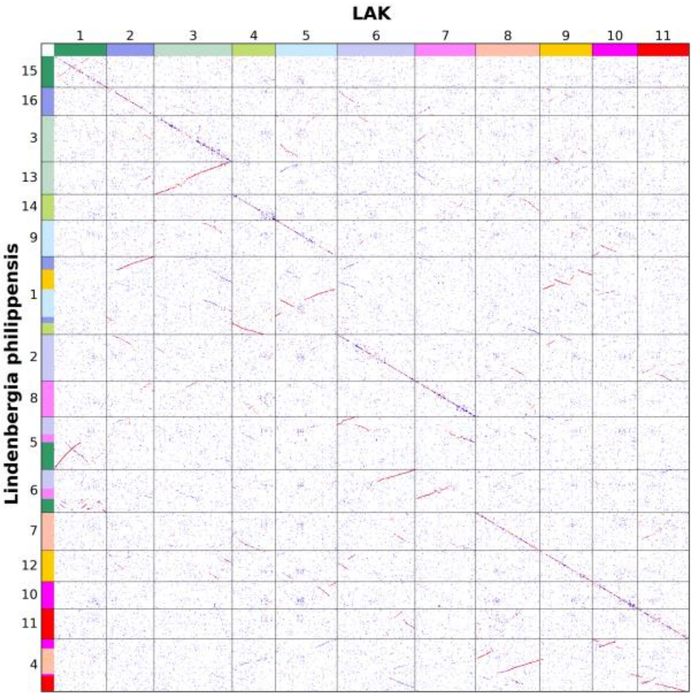

Figure 4. The syntenic dot plot of *L. philippensis* and LAK genome.

We can see that most of the genes of *L. philippensis* are in the collinear block with LAK (Figure 4). At the same time, Chr1 of *L. philippensis* can be formed by the fusion of LAK5 and LAK9 through the EEJ model and then insert into LAK2 through the NCF model, and then fused with another LAK4 again through the EEJ model, and this process reduces three ancestral chromosomes. Chr5 and Chr6 of *L. philippensis* can be formed by the fusion of LAK1 and LAK7 or LAK1 and LAK6 and or LAK6 and LAK7 through the EEJ model and then translocate chromosome arms (RTA) with another LAK6 or LAK7 or LAK1, and this process reduces one ancestral chromosome. Chr4 of *L. philippensis* can be formed by the fusion of LAK10 and LAK11 and then fused with another LAK8 again through the NCF model, and this process reduces two ancestral chromosomes. *L. Philippensis* should have 2×11=22 chromosomes in theory after two WGDs from LAK, It currently has 16 chromosomes after reducing 3+1+2=6 chromosomes. The karyotype evolution of *L. Philippensis* can be explained by the model proposed by Sun, et al. (2022), which again demonstrates the reliability of LAK (Figure 4).

This LAK result does not represent the ancestral karyotype for all Lamiales species that experienced the L-WGD event, and it requires further validation in other species. *P. fortunei* has been shown to have experienced the L-WGD event and contains relatively more chromosomes. Therefore, we used *Paulownia fortunei* to validate the LAK. As in the previous process, we used WGDI with the "-d, -icl, -bi,-c, -km, -d" parameters for comparison (Figure 5). For each protochromosome of LAK, intact homologous chromosomes can be found in *P. fortunei*, and no chromosome is the proper subset of protochromosomes. Meanwhile, *P. fortunei* retains more intact LAK except for the Chr7 (formed by the fusion of LAK2 and LAK4 through the module of EEJ) and Chr11 (formed by the fusion of LAK10 and LAK11 through the module of EEJ) (Figure 5). So, this LAK with 11 protochromosomes could be representing the ancestral karyotype for other

Lamiales species, which shared the L-WGD event.

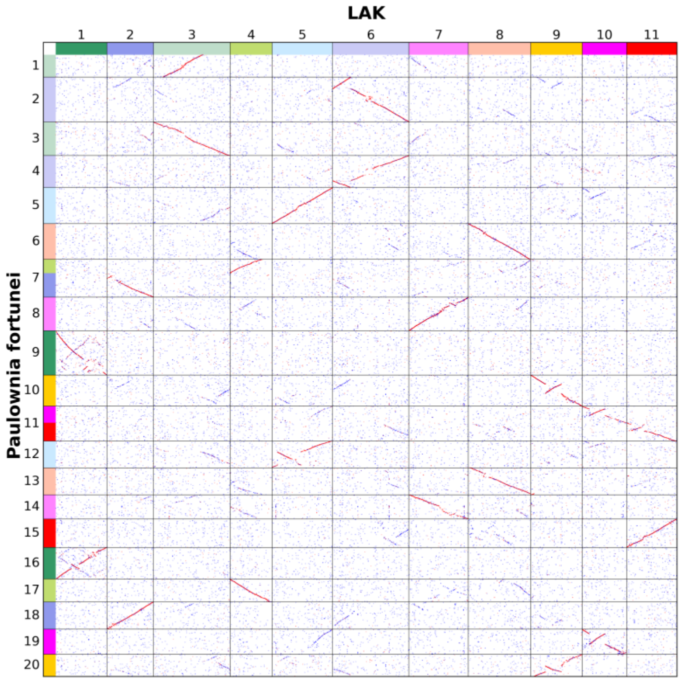

Figure 5. The syntenic dot plot of *P. fortunei* and LAK genome.

To further validate all species of Lamiales weather shared the LAK, we used a closely related species *Ophiorrhiza pumila* (Gentianales) located in the position of the sister order of Lamiales, to validate this LAK result. In other words, Lamiales and Gentianales evolved from a common ancestor in Lamiids and they share a common ancestral karyotype. As in the previous process, we used WGDI with the "-d, -icl, -bi,-c, -km, -d" parameters for comparison. Polyploidy analysis showed that *O. pumila* (Opum) has only experienced γ-WGT events, which means that *O. pumila* has one-to-one homology chromosome with LAK. As expected, most of homology chromosomes (9/11) have one-to-one correspondence, except LAK1 and LAK9 protochromosomes. The only difference is in LAK1, where LAK1-1 and LAK1-2 evolved into intact protochromosomes in LAK. In *O. pumila*, LAK1-1 and LAK9 formed the Opumchr9. These differences may be derived from the various karyotype evolutionary trajectories within the Lamiids lineage (Figure 6).

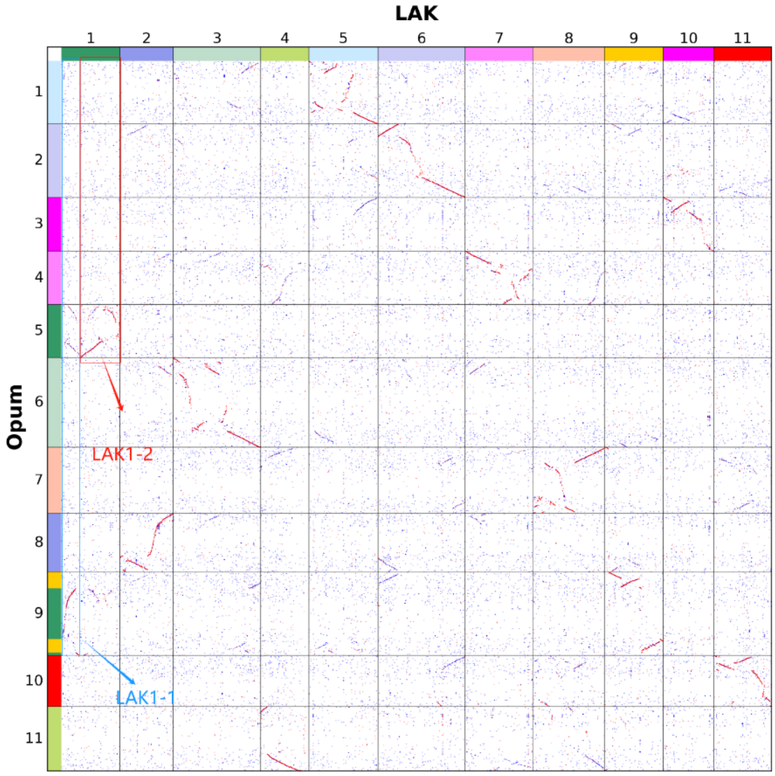

Figure 6. The syntenic dot plot of *O. pumila* and LAK genome.

To further validate the LAK1 weather evolved into an intact protochromosomes in all Lamiales species, we further select *Forsythia suspense* (Oleaceae), which located in the root of Lamiales phylogeny, except the family Plocospermataceae and Carlemanniaceae, to prove the structure of LAK1. As in the previous process, we used WGDI with the "-d, -icl, -bi,-c, -km, -d" parameters for comparison. Polyploidy analysis showed that *F. suspensa* has experienced a WGT event after γ-WGT, which means that *F. suspensa* has three copies of LAK. The dot plots between *F. suspensa* and LAK shown LAK1 showed an intact karyotype structure, indicating LAK1 had evolved an intact protochromosomes in Lamiales species (Figure 7).

From the above, LAK with 11 protochromosomes, except for the family Plocospermataceae and Carlemanniaceae, can represent the ancestral karyotype for all species of Lamiales, and can be used in subsequent analyses.

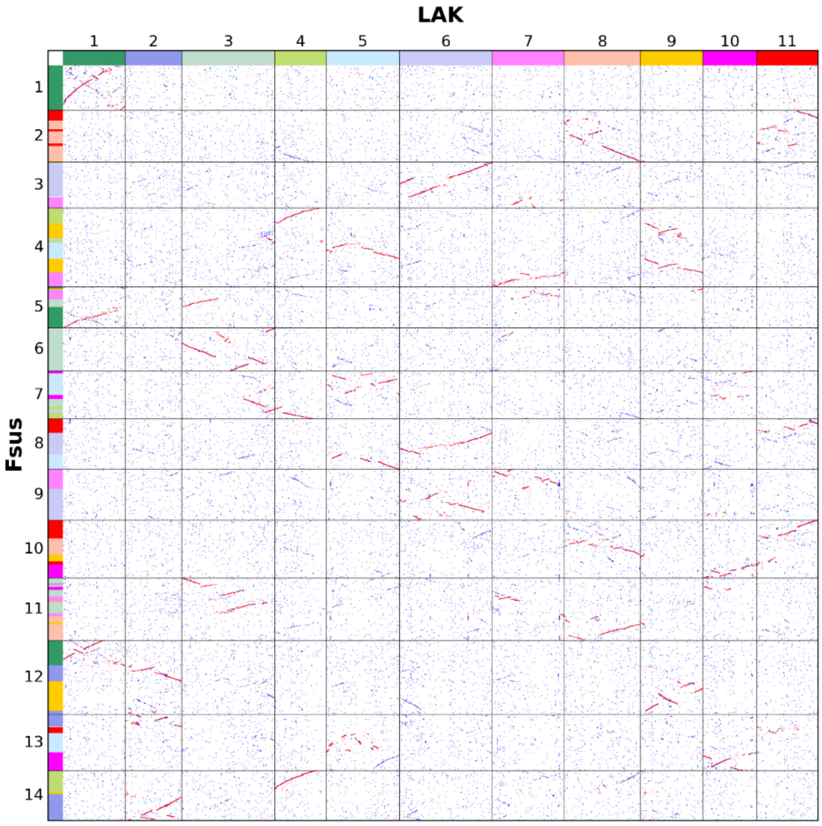

Figure 7. The syntenic dot plot of *F. suspensa* and LAK genome.

Sun P, et al. 2022. WGDI: A user-friendly toolkit for evolutionary analyses of whole-genome duplications and ancestral karyotypes. *Mol Plant* 15: 1841-1851. doi: 10.1016/j.molp.2022.10.018
